# Mortality of 196,826 Men and Women Working in U.S.-Based Petrochemical and Refinery Operations: Update 1979 to 2010

**DOI:** 10.1097/JOM.0000000000002416

**Published:** 2021-10-20

**Authors:** Nancy C. Wojcik, Elizabeth M. Gallagher, Melannie S. Alexander, R. Jeffrey Lewis

**Affiliations:** ExxonMobil Biomedical Sciences, Inc., Annandale, New Jersey (Ms Wojcik, Ms Gallagher, Dr Alexander, and Dr Lewis).

**Keywords:** asbestosis, leukemia, melanoma, mesothelioma, mortality, motor neuron disease, petrochemical, refinery, SMR, surveillance

## Abstract

To describe mortality trends of men and women working in various petrochemical and refinery operations of a U.S.-based company.

**Methods:**

The cohort consists of full-time employees with at least 1 day of service during 1979 through 2010. Standardized mortality ratios (SMRs) and 95% confidence intervals (CIs) were calculated for 111 possible causes of death studied.

**Results:**

SMRs for malignant mesothelioma and asbestosis were highest for the 1940s decade of hire. Increased SMRs were observed for malignant melanoma and motor neuron disease with no obvious work patterns. Decreasing mortality patterns were observed for aplastic anemia and acute nonlymphocytic leukemia.

**Conclusions:**

Mortality surveillance of this large established cohort aids in assessing the chronic health status of the workforce. Identifying methods for incorporating job-exposure matrices and nonoccupational risk factors could further enhance interpretations for some findings such as motor neuron disease.

## Abstract 2

The results of this mortality study provide information regarding the potential chronic health patterns observed among a cohort of U.S.-based petrochemical and refinery workers compared to the U.S. general population. This information can be used to inform those responsible for managing exposure controls, employee health programs, and regulations/policy.

Occupational exposure in the petroleum refining industry is classified as *probably carcinogenic to humans (Group 2A)* by the International Agency for Research on Cancer (IARC).^1^ IARC's decision considered evidence of skin cancer and leukemia reported in epidemiological studies as well as evidence from their evaluation of the carcinogenic effects of benzene and other polycyclic aromatic hydrocarbons. For benzene, the IARC classification is Group 1, carcinogenic to humans for lymphatic and hematopoietic (LH) cancers^2^ and also suspected for lung cancer and possibly other sites.^3^

Epidemiological research of petrochemical and refining workers, by our company^4–7^ and others,^8–24^ has provided additional health information regarding these and other potential cancers. Of note are asbestos-related malignant mesothelioma and benzene-related cancers and diseases of the lymphatic and hematopoietic tissues, generally associated with higher exposures during earlier work eras. Identification of these relationships enabled reductions in potential exposures through industrial hygiene controls, lower occupational exposure limits and enhanced employee evaluation monitoring programs.

Conducting mortality surveillance of the workforce is one method to monitor for changing patterns in these cancers and other chronic health effects which would not otherwise be captured via standard safety programs which focus on acute illness and injury. These studies can also inform us about health patterns which may be influenced by the change in workforce demographics, such as more women working in industrial/manufacturing jobs and workers staying employed beyond typical retirement age. Overall, mortality surveillance results provide science-based risk estimates which tend to inform those responsible for managing exposure controls, employee health programs and regulations/policy. Our company utilizes cohort mortality surveillance studies as part of their corporate health policy.

For these reasons, we previously established a large cohort of U.S.-based ExxonMobil petrochemical refinery workers following the merger of the two companies. This cohort was more diverse compared to the earlier heritage company cohorts, with a slightly higher proportion of younger recently hired employees (hired since 1979) and more women. Yet it still contained a sizable portion of employees hired in earlier periods to enable meaningful mortality analysis.

The original mortality experience of the cohort was investigated through December 31, 2000 using a traditional retrospective epidemiologic design. Results were reported separately for men and women.^4,5^ Standardized mortality ratios (SMRs), for most of the 95 causes of death studied, were below the mortality rates of the U.S. general population. The main exception was an increased mortality risk for malignant mesothelioma among some groups of men working in manufacturing sites who were hired in the 1940s and 1950s.

The study also found significantly increased SMRs for acute nonlymphocytic leukemia (ANLL), which combined acute myelocytic with acute monocytic, megakaryocytic, and acute erythremia/erythroleukemia, among men in the chemicals segment (SMR 1.81, 95% confidence interval (CI) 1.06–2.90), based on 17 observed deaths. Risks were also slightly elevated for motor vehicle accidents (MVA) among some groups of younger and short-term operators. A nonstatistically significant elevation of aplastic anemia (SMR 2.19, 95% CI 0.95 to 4.32) was noted for men in downstream operations, based on eight observed deaths.

Among female office/clerical workers, an elevation in ovarian cancer was found (SMR 1.40, 95% CI 1.02 to 1.87), based on 46 deaths, with no work-related patterns. In addition, MVA and other external causes of death were increased for some subgroups of blue-collar females who were younger and who had worked for shorter periods. Elevations were also seen in some of the same subgroups for chronic diseases such as cerebrovascular and respiratory disease. None of these findings showed work-related patterns and were based on 15 or fewer deaths.

Nonstatistically significant increased SMRs for amyotrophic lateral sclerosis (ALS), a degenerative motor neuron disease with uncertain occupational etiology, was also reported. The SMR for ALS in upstream men was 1.47 (95% CI 0.78–2.51) and in women, mainly in office/clerical jobs, the SMR was 1.35 (95% CI 0.65 to 2.48). Risks were associated with 1940 to 1960 hires.

In this paper, we describe updated mortality results for this U.S.-based ExxonMobil cohort. The cohort was expanded to include new employees hired since 2000 and followed mortality of the entire cohort through December 31, 2010. We note that the gap between the study end period (2010) and publication (2021) is a result of the manual processing of death certificates (see Methods) and delays due to the Covid-19 pandemic. We employed the same analytical design used in the original study to enable direct comparisons.

## METHODS

### Design

Details on the design of this retrospective occupational cohort study have been described in previous publications.^4,5^ In brief, mortality rates in the cohort were compared to expected mortality rates in a comparable portion of the U.S. general population. Eligible employees are men and women employed full-time in ExxonMobil U.S. operations for at least one day during the period of January 1, 1979 through December 31, 2010.

### Demographic and Work History

Demographic data and work history data (employment dates, operating segments and job titles) are extracted from the company's human resources (HR) database. These data are updated regularly and securely stored in ExxonMobil Health Status Registry (EHSR) which is approved by the Texas Department of Health Institutional Review Board.

We selected the first operating segment and job title for each worker to represent the potential of earlier exposure being in manufacturing or field-based jobs. Workers who are not identified as belonging to a single operating segment are placed in a “mixed” category. Workers without a record of operating segment and/or job title are placed in a “missing” category.

The operating segments are classified as: upstream, downstream, chemicals, coal and minerals, and corporate global services. Upstream is generally associated with exploration, development and production of oil and gas or other resources. Downstream includes refining operations (ie, transform crude oil and natural gas into fuels, lubricants and other finished products), and pipeline and distribution operations. The chemicals operation manufactures specialty and commodity chemicals derived from upstream and downstream products. The coal and minerals segment involves processes which extract and develop coal for fuels and minerals for various building materials and consumer products. Corporate global services include departments such as human resources, law and public affairs, security, safety and medical and occupational health.

Job titles are based on the major categories used by the Equal Employment Opportunity (EEO) Commission.^25^ We use the titles: managers/supervisors, professionals, technicians, sales, office/clericals, skilled craftsmen, operators, laborers, and service. For the EEO job titles considered to represent manufacturing and field-based titles, examples of work activities are as follows: technicians (eg, laboratory, research, engineering technicians), skilled craftsmen (eg, carpenters, machinists, mechanics, electricians, pipefitters, boilermakers, and welders), operators (eg, plant/maintenance drivers) and laborers (eg, packers, material handlers). For some managers/supervisors and professionals, their work assignments may have included manufacturing and field-based operations in the beginning of their careers.

### Vital Status and Follow-Up

Determinations of vital status are made from company records, the Social Security Administration's (SSA) service, and death certificates received from the National Death Index (NDI).^26^ Employees with an unknown vital status are classified as lost to follow-up (LTF). LTF cases are presumed alive and contribute person-time up to their last day of employment. The cohort is followed from the first day of employment until death, separation from the company or the end of the study, whichever is earlier.

### Death Certificate Coding

Underlying cause of death was coded to the International Classification of Diseases (ICD), revision in effect at time of death (ICD-9 for 1979 to 1998, ICD-10 for 1999 to 2010). The list of ICD codes is provided as a supplement (SDC Table 1). Coding was performed by a nosologist trained by the National Center for Health Statistics. When death certificates were not attainable from state office's we used the NDI Plus coded cause of death. For malignant mesothelioma deaths occurring in 1979 to 1998 (ICD-9), we followed our standardized process of manual review to flag malignant mesothelioma deaths in the EHSR.^27^

#### Analysis

This surveillance study examined 111 possible causes of death which are part of the following broader disease categories: infectious diseases, circulatory diseases, endocrine diseases, digestive diseases, respiratory diseases, blood and blood-forming organ diseases, malignant and benign cancers, and accidents / poisonings / violence.

We calculated expected deaths, person-years, SMRs and their corresponding 95% confidence intervals (CIs) using the Occupational Cohort Mortality Analysis Program (OCMAP) software package developed by the University of Pittsburgh (UPITT).^28^ U.S. population mortality rates were also obtained from UPITT. The SMR analysis is controlled for gender, race (white/non-white) and 5-year categories of age and calendar time. SMRs are only calculated when there are at least five observed deaths or five expected deaths to preserve the stability of the calculations.

SMRs for malignant mesothelioma deaths, hereafter referred to as “mesothelioma,” were calculated separately. To generate an SMR for ICD-9 mesothelioma deaths we followed our standard ascertainment procedure^27^ and used mesothelioma incidence rates obtained from the Surveillance, Epidemiology, and End Results (SEER).^29^ SMRs based on ICD-10 mesothelioma deaths used population mortality rates from UPITT. OCMAP was used to generate the expected numbers of mesothelioma deaths that occurred during the ICD-9 and ICD-10 revision periods. For this paper, we combined the ICD-9 and ICD-10 mesothelioma observed deaths and expected deaths, respectively. The sum of the observed deaths (9th + 10th) was divided by the sum of the expected deaths (9th + 10th). The SMR and 95% CI was calculated using the Mid-P exact test.^30^

The analyses focused on the mortality patterns for the total observation period (1979 to 2010) for all causes of death studied. For the a priori causes of death, we also conducted stratified analyses by study period to compare mortality results from the total observation period, the update period (2001 to 2010), and the original study (1979 to 2000) to assess if risks are increasing or decreasing. These data are provided as supplemental digital content (SDC) and are described in the Discussion. SMR results for select sub-group analyses are also provided as SDC (see Results and Discussion). All SMRs are considered statistically significant if their corresponding 95% CIs did not include 1.00. Interpretation of increased mortality risk is given more weight for a priori causes of death or if there is greater statistical stability (ie, a narrower CI).

#### Ethical Review and Data Privacy

The cohort study protocol was reviewed and approved externally by the Committee for the Protection of Human Subjects at the University of Texas, Health Science Center at Houston. It was also reviewed by an internal research ethics committee that includes two external academic advisors/ethicists. Research staff receive training in human subject research through the Collaborative Institutional Training Initiative (CITI Program). Systems, processes and confidentiality agreements protect data from unauthorized access and use, as described in the protocol, company guidelines, and agreements with national and state health agencies and in keeping with the confidentiality requirements of the Good Epidemiology Practices.^31^

## RESULTS

### Cohort Characteristics

The cohort consists of 196,826 employees who contributed 4,473,909 person-years of observation for the total observation period (1979 to 2010). Compared to the original study, this 10-year update adds 19,855 workers (11%), 27% of which are women, and 1,534,578 person-years (52%). The number of observed cohort deaths has nearly doubled since the original study (31,091 observed deaths vs. 15,025 observed deaths) and 52% of observed deaths (*n* = 16,052) occurred in the update period of 2001 through 2010.

Table 1 displays the characteristics of the cohort by gender for the total observation period. Men make up 72% of the cohort. The largest proportion of cohort members were observed in downstream operations (32%) and in the categories of manager/supervisors and professionals combined (42%). Manufacturing job titles (ie, technicians, skilled craftsmen, operators and laborers) accounted for 39% of the cohort. Less than 1% of the cohort were classified with a job title of “service”. We note that service workers performed non-industrial tasks including culinary, janitorial and security work (SMR results are not presented in this report). About 1% of the cohort work history records required manual review to assign an operating segment (not shown in Table 1).

**TABLE 1:** Characteristics of U.S. Petroleum Cohort by Gender (1979–2010)

Characteristics	Men	Women	Total	Percent
Total	141,742	55,084	196,826	Men 72%Women 28
Race				
White	124,698	45,225	169,923	86
Non-White	17,044	9,859	26,903	14
Year of birth				
<1920	4,528	610	5,138	3
1920–29	18,103	2,961	21,064	11
1930–39	15,137	4,068	19,205	10
1940–49	28,919	10,010	38,929	20
1950–59	41,047	20,358	61,405	31
1960–69	20,569	11,480	32,049	16
1970–79	9,166	3,776	12,942	6
1980–2010	4,273	1,821	6,094	3
Year of hire				
<1940	701	24	725	1
1940–49	10,444	777	11221	6
1950–59	10,920	1,362	12282	6
1960–69	14,983	2,852	17835	9
1970–79	37,082	15,153	52235	27
1980–89	36,107	21,029	57136	29
1990–99	16,211	8,005	24216	12
2000–09	11,969	4,577	16546	8
2010	3,325	1,305	4630	2
Mean age at hire	28 years	29 years	–	–
Age at last observation (death or end of study)				
<20	109	98	207	1
20–29	5,383	4,123	9,506	5
30–39	11,052	5,438	16,490	8
40–49	20,219	9,891	30,110	15
50–59	39,683	17,918	57,601	29
60–69	32,787	10,598	43,385	22
70–79	18,840	4,399	23,239	12
80+	13,669	2,619	16,288	8
*Mean age last observation*	*58 years*	*53 years*		
Duration of employment				
<5	38,722	22,558	61,280	31
5–9	18,852	10,425	29,277	15
10–19	26,687	11,827	38,514	19
20–29	24,670	6,451	31,121	16
30+	32,811	3,823	36,634	19
*Mean duration*	*16 years*	*10 years*		
Latency: time from hire to death or end of study				
<10	18,316	9,691	28,007	14
10–19	17,006	7,760	24,766	13
20+	106,420	37,633	144,053	73
*Mean latency*	*29 years*	*24 years*		
Status at end of study (12/31/2010)				
Active	25,509	8,809	34,318	17
Separated or retired	89,855	41,562	131,417	67
Deceased	26,378	4,713	31,091	16
Year of death				
1979	106	12	118	1
1980–89	3,795	489	4284	14
1990–99	8,100	1,260	9360	30
2000–09	12,747	2,609	15356	49
2010	1,630	343	1973	6
Mean age at death	68 years	64 years		
Operating segment				
Downstream	49,830	13,697	63,527	32
Chemicals	27,106	10,570	37,676	19
Upstream	26,794	9,925	36,719	19
Corporate global Services	8,929	7,318	16,247	8
Coal and minerals	3,974	947	4,921	3
Mixed	20,001	9,401	29,402	15
Missing	5,108	3,226	8,334	4
Job title: EEO				
Managers/supervisors	18,167	1,241	19,408	10
Professionals	48,284	15,472	63,756	32
Technicians	9,972	2,982	12,954	6
Sales	2,531	637	3,168	2
Office/clericals	4,806	26,309	31,115	16
Skilled craftsmen	25,838	1,114	26,952	14
Operators	22,738	3,035	25,773	13
Laborers	7,705	3,850	11,555	6
Service	1,571	394	1965	0.9
Missing	130	50	180	0.1

### Vital Status and Mortality Ascertainment

**FIGURE 1:**
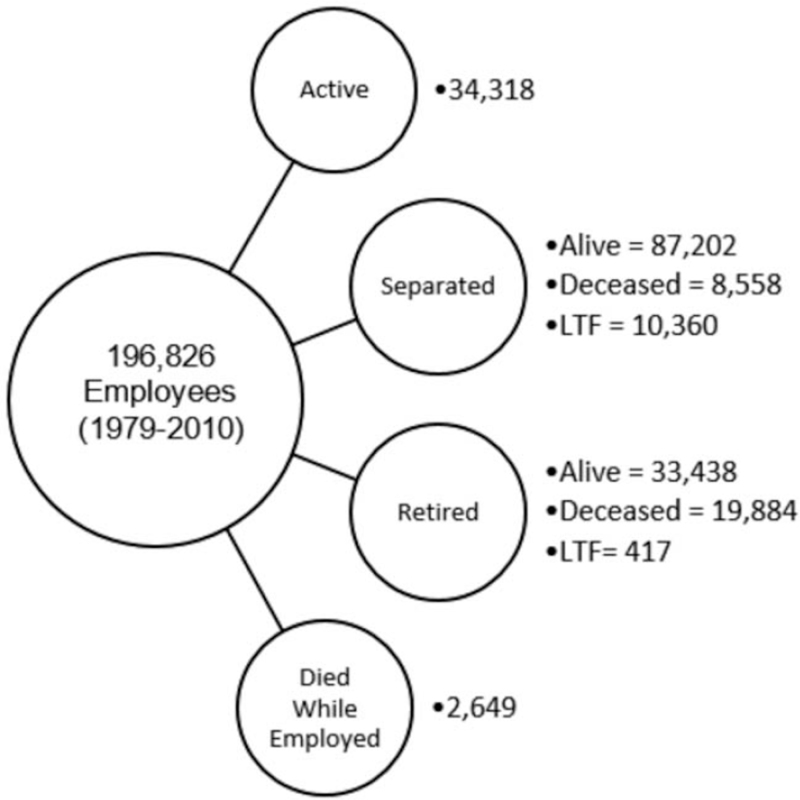
Distribution of the U.S.-based petroleum cohort by vital status and work status at end of study.

Vital status was known for 95% of the cohort. Figure 1 displays the cohorts’ distribution by vital status and work status at the end of the study. The results show that 17% of the cohort were actively employed in the company, 61% were alive and left the company (separated or retired), 16% were deceased and 5% were LTF.

For those actively employed, 17% were 55 to 59 years of age and 6% were at or beyond typical retirement age (ie, 60 to 85 years of age) (not shown in Fig. 1). Among cohort decedents, 64% were retirees and approximately 9% died while employed; of the latter, 13% worked for less than 5 years. For those who were LTF, about 74% were employed less than 10 years. Approximately 60% were classified as professionals and office/clericals, for both the first and last job. Slightly more than half of the LTF were women.

The majority of the mortality data were ascertained though company benefits (60%), followed by tracing through the NDI (39%) and the SSA (1%). We obtained a specific cause of death code for 98% of the cohort deaths. The remaining deaths were coded as unknown cause of death. While deaths occurred in all states, larger proportions were observed in states where the company has operations: Texas (31%), New Jersey (10%), and Louisiana (8%).

### All-Cause and Cause-Specific Mortality Results

There were a total of 31,091 observed deaths in the cohort (men = 26,378 and women = 4713). The SMRs, 95% CIs and observed and expected numbers of death are presented for the overall cohort and by gender in Table 2 (operating segments and job titles combined). Main findings for men and women are described separately below.

**TABLE 2:** Mortality Results of U.S. Petroleum Cohort by Gender (1979–2010)

	Overall Cohort			MEN Only			WOMEN Only		
Cause of Death	Observed	Expected^•^	SMR (95% CI)	Observed	Expected^•^	SMR (95% CI)	Observed	Expected^•^	SMR (95% CI)
All Causes	31091	43701.7	0.71 (0.70–0.72)^∗∗^	26378	37681.6	0.70 (0.69–0.71)^∗∗^	4713	6020.1	0.78 (0.76–0.81)^∗∗^
Infectious and parasitic diseases	808	1357.6	0.60 (0.56–0.64)^∗∗^	684	1179.9	0.58 (0.54–0.62)^∗∗^	124	177.7	0.70 (0.58–0.83)^∗∗^
Tuberculosis	5	26.4	0.19 (0.06–0.44)^∗∗^	5	23.6	0.21 (0.07–0.50)^∗∗^	0	2.8	–
Human Immunodeficiency Virus (HIV) disease (incl. AIDS)	297	560.1	0.53 (0.47–0.59)^∗∗^	274	512.9	0.53 (0.47–0.60)^∗∗^	23	47.2	0.49 (0.31–0.73)^∗∗^
Malignant Neoplasms (MNs)	9680	11998.8	0.81 (0.79–0.82)^∗∗^	7920	10108.7	0.78 (0.77–0.80)^∗∗^	1760	1890.1	0.93 (0.89–0.98)^∗∗^
MN of Buccal cavity and Pharynx	148	232.4	0.64 (0.54–0.75)^∗∗^	134	213.1	0.63 (0.53–0.74)^∗∗^	14	19.2	0.73 (0.40–1.22)
MN of Pharynx	73	119.4	0.61 (0.48–0.77)^∗∗^	65	110.7	0.59 (0.45–0.75)^∗∗^	8	8.8	0.91 (0.39–1.80)
MN of digestive organs and Peritoneum	2293	2867.1	0.80 (0.77–0.83)^∗∗^	1966	2498.7	0.79 (0.75–0.82)^∗∗^	327	368.5	0.89 (0.79–0.99)^∗^
MN of Esophagus	259	362.6	0.71 (0.63–0.81)^∗∗^	238	344.0	0.69 (0.61–0.79)^∗∗^	21	18.5	1.13 (0.70–1.73)
MN of stomach	229	291.8	0.78 (0.69–0.89)^∗∗^	201	260.2	0.77 (0.67–0.89)^∗∗^	28	31.6	0.89 (0.59–1.28)
MN of large intestine (Colon)	750	933.1	0.80 (0.75–0.86)^∗∗^	614	794.4	0.77 (0.71–0.84)^∗∗^	136	138.7	0.98 (0.82–1.16)
MN of rectum	142	172.5	0.82 (0.69–0.97)^∗^	117	149.7	0.78 (0.65–0.94)^∗∗^	25	22.8	1.10 (0.71–1.62)
MN of biliary passages (including gallbladder)/liver	291	388.9	0.75 (0.66–0.84)^∗∗^	258	342.9	0.75 (0.66–0.85)^∗∗^	33	46.1	0.72 (0.49–1.10)
MN of liver (specified primary or unspecified)	195	271.4	0.72 (0.62–0.83)^∗∗^	179	249.1	0.72 (0.62–0.83)^∗∗^	16	22.3	0.72 (0.41–1.16)
MN of pancreas	549	626.6	0.88 (0.80–0.95)^∗∗^	479	531.7	0.90 (0.82–0.98)^∗^	70	94.9	0.74 (0.58–0.93)^∗∗^
MN of respiratory system	2918	4070.9	0.72 (0.69–0.74)^∗∗^	2512	3591.1	0.70 (0.67–0.73)^∗∗^	406	479.8	0.85 (0.77–0.93)^∗∗^
MN of nasal cavity/mid ear/accessory sinuses	6	11.9	0.50 (0.18–1.10)	4	10.5	0.38 (0.10–0.98)^∗^	2	1.4	–
MN of larynx	51	121.6	0.42 (0.31–0.55)^∗∗^	48	115.2	0.42 (0.31–0.55)^∗∗^	3	6.4	0.47 (0.10–1.38)
MN of bronchus, trachea, lung	2846	3919.5	0.73 (0.70–0.75)^∗∗^	2448	3449.5	0.71 (0.68–0.74)^∗∗^	398	470.0	0.85 (0.77–0.93)^∗∗^
MN of bone	9	23.3	0.39 (0.18–0.74)^∗∗^	8	19.7	0.41 (0.18–0.80)^∗∗^	1	3.5	–
MN of connective tissue	90	77.8	1.16 (0.93–1.42)	73	62.5	1.17 (0.92–1.47)	17	15.3	1.11 (0.65–1.78)
MN of skin	296	276.5	1.07 (0.95–1.20)	257	247.5	1.04 (0.92–1.17)	39	29.1	1.34 (0.95–1.83)
Malignant melanoma	255	212.7	1.20 (1.06–1.36)^∗∗^	219	187.6	1.17 (1.02–1.33)^∗^	36	25.1	1.44 (1.01–1.99)^∗^
Malignant mesothelioma	123	78.5	1.57 (1.31–1.86)^∗∗^	120	74.6	1.61 (1.34–1.92)^∗∗^	3	3.9	–
MN of breast	393	382.6	1.03 (0.93–1.13)	5	12.7	0.40 (0.13–0.92)^∗^	388	369.9	1.05 (0.95–1.16)
MN of cervix uteri	39	47.1	0.83 (0.59–1.13)	0	0	–	39	47.1	0.83 (0.59–1.13)
MN of body of uterus (including corpus Uteri)	23	22.4	1.03 (0.65–1.54)	0	0	–	23	22.4	1.03 (0.65–1.54)
MN of ovary	120	110.5	1.09 (0.90–1.30)	0	0	–	120	110.5	1.09 (0.90–1.30)
MN of prostate	706	836.7	0.84 (0.78–0.91)^∗∗^	706	836.7	0.84 (0.78–0.91)^∗∗^	0	0	–
MN of testicular	5	12.7	0.39 (0.13–0.92)^∗^	5	12.7	0.39 (0.13–0.92)^∗∗^	0	0	–
MN of bladder and other urinary	228	295.1	0.77 (0.68–0.88)^∗∗^	198	275.6	0.72 (0.62–0.83)^∗∗^	30	19.5	1.54 (1.04–2.20)^∗^
MN of bladder (Monson)	224	289.3	0.77 (0.68–0.88)^∗∗^	195	270.7	0.72 (0.62–0.83)^∗∗^	29	18.7	1.55 (1.04–2.23)^∗^
MN of kidney	250	305.4	0.82 (0.72–0.93)^∗∗^	217	274.2	0.79 (0.69–0.90)^∗∗^	33	31.2	1.06 (0.73–1.49)
MN of central nervous system (CNS) including brain	299	315.7	0.95 (0.84–1.06)	258	266.3	0.97 (0.85–1.09)	41	49.4	0.83 (0.60–1.14)
MN of brain	293	310.0	0.94 (0.84–1.06)	254	261.6	0.97 (0.86–1.10)	39	48.4	0.81 (0.57–1.10)
MN of other/ill-defined sites/secondary neoplasms	664	832.8	0.80 (0.74–0.86)^∗∗^	548	711.0	0.77 (0.71–0.84)^∗∗^	116	121.7	0.95 (0.79–1.14)
MN of lymphatic and hematopoietic tissue	1056	1162.5	0.91 (0.85–0.96)^∗∗^	929	1004.2	0.92 (0.87–0.99)^∗^	127	158.3	0.80 (0.67–0.95)^∗^
Hodgkin lymphoma	42	38.0	1.11 (0.80–1.49)	34	32.0	1.06 (0.74–1.48)	8	6.0	1.34 (0.58–2.64)
Non-Hodgkin lymphoma	388	441.5	0.88 (0.79–0.97)^∗^	345	381.0	0.91 (0.81–1.01)	43	60.5	0.71 (0.52–0.96)^∗^
Nodular/follicular lymphoma	6	5.1	1.17 (0.43–2.54)	5	4.3	1.16 (0.38–2.70)	1	0.8	–
Reticulosarcoma	21	24.3	0.86 (0.54–1.32)	20	20.9	0.96 (0.58–1.48)	1	3.3	–
T-cell lymphoid variety	2	3.1	–	1	2.7	–	1	0.4	–
Lymphosarcoma	10	11.4	0.88 (0.42–1.61)	9	10.1	0.89 (0.41–1.69)	1	1.3	–
Other lymphomas	321	365.2	0.88 (0.78–98.0)^∗^	284	314.7	0.90 (0.80–1.01)	37	50.5	0.73 (0.52–1.10)
Multiple myeloma	184	209.9	0.88 (0.76–1.01)	159	180.1	0.88 (0.75–1.03)	25	29.7	0.84 (0.54–1.24)
Leukemia and aleukemia	420	445.8	0.94 (0.85–1.04)	372	387.1	0.96 (0.87–1.06)	48	58.7	0.82 (0.60–1.08)
Acute lymphocytic leukemia (ALL)	16	23.8	0.67 (0.38–1.09)	12	19.5	0.62 (0.32–1.08)	4	4.3	–
Chronic lymphocytic leukemia (CLL)	81	89.0	0.91 (0.72–1.13)	74	81.2	0.91 (0.72–1.14)	7	7.8	0.90 (0.36–1.85)
Hairy cell leukemia	1	3.3	–	1	3.1	–	0	0.2	–
Acute myelocytic leukemia (AML)	171	157.3	1.09 (0.93–1.26)	148	133.7	1.11 (0.94–1.30)	23	23.6	0.97 (0.62–1.46)
Chronic myelocytic leukemia (CML)	38	42.5	0.89 (0.63–1.23)	34	36.5	0.93 (0.64–1.30)	4	6.0	0.66 (0.18–1.70)
Acute monocytic leukemia	3	3.3	–	3	2.9	–	0	0.4	–
Chronic monocytic leukemia	0	0.3	–	0	0.3	–	0	0	–
Acute erythremia and erythroleukemia	0	1.5	–	0	1.3	–	0	0.1	–
Megakaryocytic leukemia	0	0.4	–	0	0.4	–	0	0.1	–
Acute non-lymphocytic leukemia (ANLL)	174	162.5	1.07 (0.92–1.24.)	151	138.3	1.09 (0.92–1.28)	23	24.2	0.95 (0.60–1.42)
Other/unspecified leukemia (besides ANLL, CML, ALL, CLL)	111	127.9	0.87 (0.71–1.04)	101	111.6	0.91 (0.74–1.10)	10	16.2	0.62 (0.30–1.13)
Benign/in situ/uncertain behavior/unspecified neoplasms	190	205.2	0.93 (0.80–1.07)	164	174.1	0.94 (0.80–1.10)	26	31.1	0.84 (0.55–1.22)
Benign CNS (including brain)	8	7.3	1.09 (0.47–2.15)	5	5.7	0.88 (0.29–2.05)	3	1.6	–
Benign brain	2	1.6	–	1	1.3	–	1	0.4	–
Uncertain behavior/unspecified—brain/spinal cord	56	53.2	1.05 (0.80–1.37)	45	44.1	1.02 (0.74–1.37)	11	9.1	1.21 (0.60–2.17)
All diseases of blood and blood-forming organs	128	151.1	0.85 (0.71–1.01)	102	122.0	0.84 (0.68–1.02)	26	29.1	0.89 (0.58–1.31)
Aplastic anemia	18	18.0	1. 00 (0.59–1.58)	15	15.0	1.00 (0.56–1.65)	3	3.0	–
All other anemias	29	41.0	0.71 (0.47–1.02)	21	32.0	0.66 (0.41–1.00)	8	9.0	0.89 (0.38–1.74)
All other diseases of blood-forming organs	50	47.8	1.05 (0.78–1.38)	42	39.1	1.07 (0.77–1.45)	8	8.7	0.92 (0.40–1.82)
Other specified diseases of blood/blood-form Org (including MDS)	96	94.0	1.02 (0.83–1.25)	91	84.1	1.08 (0.87–1.33)	5	9.9	0.50 (0.16–1.17)
Endocrine/nutritional/metabolic diseases	884	1539.9	0.57 (0.54–0.61)^∗∗^	749	1281.3	0.58 (0.54–0.63)^∗∗^	135	258.6	0.52 (0.44–0.62)^∗∗^
Diabetes mellitus	638	1165.7	0.55 (0.51–0.60)^∗∗^	547	973.5	0.56 (0.52–0.61)^∗∗^	91	192.2	0.47 (0.38–0.58)^∗∗^
Mental disorders	522	803.9	0.65 (0.60–0.71)^∗∗^	426	676.1	0.63 (0.57–0.69)^∗∗^	96	127.8	0.75 (0.61–0.92)^∗∗^
Alcoholism	65	202.8	0.32 (0.25–0.41)^∗∗^	50	186.1	0.27 (0.20–0.35)^∗∗^	15	16.7	0.90 (0.50–1.48)
Drug psychosis, dependence, poisoning	167	371.7	0.45 (0.38–0.52)^∗∗^	125	310.6	0.40 (0.34–0.48)^∗∗^	42	61.1	0.69 (0.50–0.93)^∗^
Nervous system/sense organ disease	1146	1266.8	0.91 (0.85–0.96)^∗∗^	953	1047.0	0.91 (0.85–0.97)^∗∗^	193	219.8	0.88 (0.76–1.01)
Parkinson's disease	285	266.3	1.07 (0.95–1.20)	258	243.3	1.06 (0.94–1.20)	27	23.0	1.17 (0.77–1.71)
Motor neuron disease including amyotrophic lateral sclerosis	152	119.4	1.27 (1.08–1.49)^∗∗^	122	101.1	1.21 (1.00–1.44)^∗^	30	18.3	1.64 (1.11–2.34)^∗^
Multiple sclerosis	32	58.8	0.54 (0.37–0.77)^∗∗^	17	38.9	0.44 (0.26–0.70)^∗∗^	15	19.9	0.75 (0.42–1.24)
Circulatory disease	10779	15659.9	0.69 (0.68–0.70)^∗∗^	9545	13832.8	0.69 (0.68–0.70)^∗∗^	1234	1827.1	0.68 (0.64–0.71)^∗∗^
All heart disease	8703	12667.6	0.69 (0.67–0.70)^∗∗^	7788	11312.8	0.69 (0.67–0.70)^∗∗^	915	1354.8	0.68 (0.63–0.72)^∗∗^
Hypertension with heart disease	317	478.7	0.66 (0.59–0.74)^∗∗^	265	404.9	0.65 (0.58–0.74)^∗∗^	52	73.8	0.71 (0.53–0.92)^∗∗^
Ischemic heart disease	6145	9000.4	0.68 (0.67–0.70)^∗∗^	5574	8143.9	0.68 (0.67–0.70)^∗∗^	571	856.5	0.67 (0.61–0.72)^∗∗^
Acute myocardial infarction	2793	4092.1	0.68 (0.66–0.71)^∗∗^	2574	3718.5	0.69 (0.67–0.72)^∗∗^	219	373.5	0.59 (0.51–0.67)^∗∗^
Hypertension without heart disease	134	245.7	0.55 (0.46–0.65)^∗∗^	110	201.8	0.54 (0.45–0.66)^∗∗^	24	43.8	0.55 (0.35–0.82)^∗∗^
Cerebrovascular disease	1408	2032.7	0.69 (0.66–0.73)^∗∗^	1167	1687.6	0.69 (0.65–0.73)^∗∗^	241	345.1	0.70 (0.61–0.79)^∗∗^
Diseases of arteries/veins/other circulatory	534	713.6	0.75 (0.69–0.82)^∗∗^	480	630.2	0.76 (0.70–0.83)^∗∗^	54	83.4	0.65 (0.49–0.84)^∗∗^
Aortic aneurysm	260	330.9	0.79 (0.69–0.89)^∗∗^	243	304.7	0.80 (0.70–0.90)^∗∗^	17	26.2	0.65 (0.38–1.04)
Non-malignant respiratory disease	2305	3737.9	0.62 (0.59–0.64)^∗∗^	1932	3220.0	0.60 (0.57–0.63)^∗∗^	373	517.9	0.72 (0.65–0.80)^∗∗^
Acute respiratory infections except influenza/pneumonia	6	7.5	0.80 (0.30–1.75)	5	6.2	0.81 (0.26–1.90)	1	1.3	–
Pneumonia	493	878.2	0.56 (0.51–0.61)^∗∗^	429	763.1	0.56 (0.51–0.62)^∗∗^	64	115.0	0.56 (0.43–0.71)^∗∗^
Influenza	14	14.3	0.98 (0.54–1.64)	12	11.6	1.03 (0.53–1.81)	2	2.7	–
Bronchitis, Emphysema, and asthma	286	424.7	0.67 (0.60–0.76)^∗∗^	221	359.1	0.62 (0.54–0.70)^∗∗^	65	65.7	0.99 (0.76–1.26)
Bronchitis	18	34.1	0.53 (0.31–0.84)^∗∗^	13	29.6	0.44 (0.23–0.75)^∗∗^	5	4.4	1.13 (0.36–2.63)
Emphysema	228	321.1	0.71 (0.62–0.81)^∗∗^	182	281.3	0.65 (0.56–0.75)^∗∗^	46	39.8	1.16 (0.85–1.54)
Asthma	40	69.5	0.58 (0.41–0.78)^∗∗^	26	48.1	0.54 (0.35–0.79)^∗∗^	14	21.4	0.65 (0.36–1.10)
Pneumoconiosis and other respiratory diseases	1506	2413.3	0.62 (0.59–0.66)^∗∗^	1265	2080.1	0.61 (0.58–0.64)^∗∗^	241	333.2	0.72 (0.64–0.82)^∗∗^
Chronic obstructive pulmonary disease	985	1731.2	0.57 (0.53–0.61)^∗∗^	807	1487.1	0.54 (0.51–0.58)^∗∗^	178	244.1	0.73 (0.63–0.84)^∗∗^
Pneumoconiosis/other lung diseases, external agents	153	238.2	0.64 (0.54–0.75)^∗∗^	135	215.1	0.63 (0.53–0.74)^∗∗^	18	23.1	0.78 (0.46–1.23)
Asbestosis	27	13.8	1.96 (1.29–2.85)^∗∗^	27	13.7	1.97 (1.30–2.87)^∗∗^	0	0.1	–
Silicosis and anthracosilicosis	7	15.8	0.44 (0.18–0.92)^∗^	7	15.7	0.44 (0.18–0.92)^∗^	0	0.1	–
Digestive disease	994	1809.5	0.55 (0.52–0.59)^∗∗^	818	1555.6	0.53 (0.49–0.56)^∗∗^	176	253.8	0.69 (0.60–0.80)^∗∗^
Ulcer of stomach and duodenum	34	81.5	0.42 (0.29–0.58)^∗∗^	28	71.4	0.39 (0.26–0.57)^∗∗^	6	10.1	0.59 (0.22–1.29)
Cirrhosis of liver	460	869.8	0.53 (0.48–0.58)^∗∗^	386	769.5	0.50 (0.45–0.55)^∗∗^	74	100.3	0.74 (0.58–0.93)^∗∗^
Genitourinary Disease	539	778.3	0.69 (0.64–0.75)^∗∗^	445	654.9	0.68 (0.62–0.75)^∗∗^	94	123.5	0.76 (0.62–0.93)^∗∗^
Nephritis and nephrosis	426	589.2	0.72 (0.66–0.80)^∗∗^	355	501.2	0.71 (0.64–0.79)^∗∗^	71	87.9	0.81 (0.63–1.03)
Skin/subcutaneous tissue disease	30	47.2	0.64 (0.43–0.91)^∗^	22	37.6	0.59 (0.37–0.89)^∗∗^	8	9.6	0.83 (0.36–1.64)
Musculoskeletal disease and connective tissue	87	157.6	0.55 (0.44–0.68)^∗∗^	58	107.4	0.54 (0.41–0.70)^∗∗^	29	50.2	0.58 (0.39–0.83)^∗∗^
All external causes of death	2463	3648.1	0.68 (0.65–0.70)^∗∗^	2114	3238.4	0.65 (0.62–0.68)^∗∗^	349	409.7	0.85 (0.76–0.95)^∗∗^
Accidents	1473	2190.3	0.67 (0.64–0.71)^∗∗^	1271	1928.8	0.66 (0.62–0.70)^∗∗^	202	261.5	0.77 (0.67–0.89)^∗∗^
Transportation accidents	728	1028.5	0.71 (0.66–0.76)^∗∗^	622	905.1	0.69 (0.63–0.74)^∗∗^	106	123.4	0.86 (0.70–1.04)
Motor vehicle accidents (MVA)	613	819.4	0.75 (0.69–0.81)^∗∗^	524	719.0	0.73 (0.67–0.79)^∗∗^	89	100.3	0.89 (0.71–1.09)
All other accidents besides MVA	856	1351.3	0.63 (0.59–0.68)^∗∗^	743	1192.2	0.62 (0.58–0.67)^∗∗^	113	159.1	0.71 (0.58–0.85)^∗∗^
Suicides	725	907.7	0.80 (0.74–0.86)^∗∗^	643	828.4	0.78 (0.72–0.84)^∗∗^	82	79.2	1.04 (0.82–1.28)
Homicides and legal intervention	200	423.2	0.47 (0.41–0.54)^∗∗^	148	375.3	0.39 (0.33–0.46)^∗∗^	52	47.9	1.09 (0.81–1.42)
Congenital anomalies	39	79.9	0.49 (0.35–0.67)^∗∗^	31	62.6	0.50 (0.34–0.70)^∗∗^	8	17.3	0.46 (0.20–0.91)^∗^

SMR (95% CI), standardized mortality ratio (95% confidence interval).

• Expected deaths based on U.S. general population mortality rates.

∗ Statistically significant at *P* < 0.05.

∗∗ Statistically significant at *P* < 0.01. MDS, Myelodysplastic Syndrome.

### MEN

The mortality risk for all-causes combined was 30% lower in men in the cohort compared to the mortality rate for men in the U.S. general population for the total observation period of 1979 through 2010 (Table 2). Risks were also 20% to 47% lower than background (unity) for most major categories of death including circulatory, digestive. and cerebrovascular diseases.

#### Malignant Mesothelioma (aka “mesothelioma”)

With this update, the number of observed mesothelioma deaths has doubled (120 vs. 64 observed deaths) for men, resulting in an increased SMR of 1.61 (95% CI 1.34 to 1.92) (Table 2). Most mesothelioma cohort deaths occurred in white men (SMR 1.58, 95% CI 1.31 to 1.89) (not shown in Table 2). Among nonwhite men, the SMR (2.61) just reached statistical significance (95% CI 1.065.42), based on 6 observed deaths vs. 2.3 expected deaths. We note that two male decedents, hired in their 40s, had shorter latencies of 3 and 5 years while the average latency was about 40 years.

Mesothelioma mortality risks for men are summarized by selected work factors in Table 3. By period of hire, the SMR was highest among men hired in the 1940s (SMR 2.36, 95% CI 1.78 to 3.08). For the 1950s period of hire, the SMR was 1.57 and just reached significance (95% CI 1.07 to 2.20). For the 1960s hire period, the SMR of 1.46 did not reach significance (95% CI 0.91-2.24). Overall, 70% of the observed mesothelioma deaths were among men hired before 1960 resulting in an SMR of 1.98 (95% CI 1.59 to 2.44) (not shown in Table 3).

**TABLE 3:** Summary of Malignant Mesothelioma Mortality by Selected Work Factors (1979–2010)—MEN (All Operating Segments and Job Titles Combined)

	SMR (95% CI), Observed/Expected^•^ Deaths
Period of hire	
<1940	−3/1.6
1940–1949	2.36 (1.78–3.08) 51/21.6^∗∗^
1950–1959	1.57 (1.07–2.20) 30/19.2^∗^
1960–1969	1.46 (0.91–2.24) 19/13.0
1970–1979	1.04 (0.58–1.73) 13/12.5
1980–1989	0.53 (0.13–1.43) 3/5.7
1990–1999	-- 1/0.8
2000–2010	-- 0/0.1
Age of hire: years	
<25	2.15 (1.64–2.78) 56/26.0^∗∗^
25–29	1.42 (0.97–2.00) 30/21.2
30+	1.24 (0.88–1.72) 34/27.3
Duration (years of employment)	
0–9	0.78 (0.32–1.62) 6/7.7
10–19	1.26 (0.70–2.10) 13/10.3
20+	1.78 (1.46–2.16) 101/56.6^∗∗^
Latency (years from first hire to death)	
0–9	−2/0.2
10–19	−3/1.1
20+	1.57 (1.30–1.88) 115/73.3^∗∗^
Operating segment	
Downstream	2.62 (2.11–3.23) 84/32.0^∗∗^
Upstream	0.81 (0.42–1.41) 11/13.6
Chemicals	0.97 (0.49–1.73) 10/10.3
Coal and minerals	−0/1.6
Corporate global services	−2/4.8
Mixed	0.86 (0.35–1.78) 6/7.0
Missing	1.32 (0.58–2.61) 7/5.3
Job title	
Managers/supervisors	1.88 (1.33–2.57) 36/19.2^∗∗^
Professionals	1.12 (0.75–1.60) 27.24.2
Skilled craftsmen	2.90 (2.10–3.91) 40/13.8^∗∗^
Operators	0.79 (0.32–1.64) 6/7.6
Technicians	1.92 (0.94–3.51) 9/4.7^∗^
All other (sales, office/clericals, laborers, service, missing)	0.4 (0.07–1.32) 2/5.0

SMR (95% CI), standardized mortality ratio (95% confidence interval).

• Expected deaths based on U.S. general population mortality rates.

∗ Statistically significant at *P* < 0.05.

∗∗ Statistically significant at *P* < 0.01.

Table 3 also shows risk was increased among men employed for 20+ years (SMR 1.78, 95% CI 1.462.16) and those with 20+ years of latency (SMR 1.57, 95% CI 1.30 to 1.88). Risk was concentrated in downstream operations (SMR 2.62, 95% CI 2.11 to 3.23), among skilled craftsmen (SMR 2.90, 95% CI 2.10 to 3.91) and managers/supervisors (SMR 1.88, 95% CI 1.33 to 2.57).

#### Asbestosis

A 2-fold statistically significant increase for asbestosis was noted among men based on 27 observed deaths (SMR 1.97, 95% CI 1.30 to 2.87) (Table 2). Nearly all decedents were white, hired before 1960 (SMR 2.67, 95% CI 1.71 to 4.00, 24 observed deaths), employed for 30 or more years (SMR 2.73, 95% CI 1.73 to 4.09, 23 observed deaths), and had 30 or more years of latency (SMR 2.26, 95% CI 1.48 to 3.31, 26 observed deaths) (data not shown in Table 2).

In downstream operations, a 3-fold statistically significant asbestosis risk was observed (SMR 2.97, 95% CI 1.76 to 4.70) (SDC Table 2). Risk was concentrated among those hired before 1960 (SMR 3.81, 95% CI 2.22 to -6.10, 17 observed deaths), employed for 30 plus years (SMR 3.84, 2.19, 6.23, 16 observed deaths) and among those with 30 plus years of latency (SMR 3.33, 95% CI 1.97 to 5.26, 18 observed deaths) (not shown in SDC Table 2). Risk was also concentrated among skilled craftsmen (all operating segments combined) (SMR 5.27, 95% CI 2.81 to 9.02) (SDC Table 3).

#### Malignant Melanoma

The SMR for malignant melanoma (SMR 1.17) just reached statistical significance (95% CI 1.02 to 1.33) in men (Table 2). Most decedents were white. When stratified by time period, we found elevated SMRs for men hired before 1960 (SMR 1.39, 95% CI 1.13 to 1.69, 101 observed deaths) and for those with less than 20 years of employment (SMR 1.37, 95% CI 1.11 to 1.68, 89 observed deaths). For men with 30 or more years of latency, the SMR (1.30) just reached significance (95% CI 1.09 to 1.54, 135 observed deaths) (not shown in Table 2).

Malignant melanoma mortality risk was also increased among men working in corporate global services (SMR 1.93, 95% CI 1.22 to 2.89, 23 observed deaths) (SDC Table 4) and male professionals (operating segments combined) (SMR 1.37, 95% CI 1.10 to 1.70) (SDC Table 5).

#### Motor Neuron Disease (MND) Including ALS and Other MNDs

The SMR for MND among men was 1.21 and just reached statistical significance (95% CI 1.00 to 1.44) (Table 2). By time period, mortality risk increased for those hired before 1960, with the SMR of 1.39 just reaching significance (95% CI 1.06 to 1.79, 60 observed deaths) and for those with 30 or more years of latency (SMR 1.44, 95% CI 1.16 to 1.76, 92 observed deaths) (not shown in Table 2). About 75% of decedents were 60 years or older at death.

Examination of the death certificates showed that 81% (99/122) of the causes of death contained the terms ALS, bulbar ALS, progressive ALS or Lou Gehrig's disease. Non-ALS disorders made-up 13% of the death certificate causes including progressive supranuclear palsy (*n* = 13), which is not considered a subtype of MND,^32,33^ bulgar palsy (*n* = 1), progressive muscular atrophy (*n* = 1) and primary lateral sclerosis (*n* = 1). The remaining seven certificates indicated the nonspecific terms of motor neuron disease, of which two specified familial MND and Kennedy's syndrome MND, which is a hereditary disorder. Military service was indicated on the death certificates for 67 of the 122 male decedents (55%).

#### Parkinson's Disease

Among non-white men, the SMR for Parkinson's disease (SMR 2.06) just reached statistical significance (95% CI 1.03 to 3.69), based on 11 observed deaths vs. 5.3 expected deaths (not shown in Table 2).

#### Brain Cancer

For men working in upstream operations, the SMR for brain cancer (SMR 1.36) just reached statistical significance (95% CI 1.05 to 1.75), based on 63 observed deaths (SDC Table 2). Decedents are mainly white and half were hired between the ages of 20 and 24 years (SMR 2.14, 95% CI 1.45 to 3.04, 31 observed deaths) (not shown in SDC Table 2). Among this age group, the SMR increased for those hired before 1960 (SMR 2.01, 95% CI 1.17 to 3.21) based on 17 observed versus 8.5 expected deaths. In other time period analyses, SMRs are elevated and just reached statistical significance for those employed for 30 plus years (SMR 1.51, 95% CI 1.00 to 2.18, 28 observed deaths) and among those with 20 plus years of latency (SMR 1.77, 95% CI 1.07 to 2.77, 19 observed deaths) (not shown in SDC Table 2).

### WOMEN

As shown in Table 2, all-cause mortality risk was 22% lower in women in the cohort compared to the mortality rate for women in the U.S. general population for the total observation period. Risks were about 30% lower than background for most major categories of death including circulatory, digestive and cerebrovascular diseases.

#### Malignant Melanoma

For malignant melanoma, the SMR (1.44) just reached significance (95% CI 1.01 to 1.99) for women (Table 2). All decedents are white. Analysis by time period showed SMRs significantly increased among white women employed less than 10 years (SMR 1.99, 95% CI 1.22 to 3.08, 20 observed deaths) and among those with 20 to 29 years of latency (SMR 2.19, 95% CI 1.30 to 3.48, 16 observed vs. 7.3 expected deaths) (not shown in Table 2). Risk was increased among female office/clericals (SMR 1.68, 95% CI 1.08 to 2.51) (SDC Table 6).

#### Bladder Cancer

The SMR for bladder cancer (1.55) also just reached significance (95% CI 1.04 to 2.23), based on 29 observed deaths in mainly white women. By time period, risk increased for women employed for 20 to 29 years (SMR 2.48, 95% CI 1.19 to 4.56) based on 10 observed and 4.0 expected deaths (not shown in Table 2). Risk was increased among women working in corporate global services (SMR 2.97, 95% CI 1.36 to 5.64), based on 9 observed and 3.0 expected deaths, mainly white women (SDC Table 7).

#### Motor Neuron Disease Including ALS and Other MNDs

The risk of MND mortality in women was increased (SMR 1.64, 95% CI 1.11 to 2.34), based on 30 observed deaths (Table 2), mainly white women. Among those employed for 10 to 19 years, the SMR (2.16) just reached statistical significance (95% CI 1.08 to 3.87) based on 11 observed vs. 5.1 expected deaths. Increased risk was also noted for those with 30 plus years of latency (SMR 2.33, 95% CI 1.36 to 3.73) based on 17 observed deaths (not shown in Table 2).

Examination of the death certificates showed that 26 of the 30 certificates (87%) indicated ALS as the cause of death and the remaining four stated MND of progressive supranuclear palsy, which is not ALS and is not considered a subtype of MND.^32,33^ Military service was indicated on one of the thirty death certificates.

For women working in downstream operations, MND mortality risk was increased (SMR 2.32 95% CI 1.16 to 4.16) based on 11 observed deaths (SDC Table 8). Of these, seven were classified as office/clericals but the SMR (2.14) did not reach statistical significance (95% CI 0.86 to 4.41) (not shown in SDC Table 8). By time period, mortality risk increased among women in downstream operations with 30 plus years of latency (SMR 3.10, 95% CI 1.24 to 6.38), based on 7 observed and 2.3 expected deaths) (not shown in SDC Table 8). Examination of the death certificates showed that seven of the eleven causes of death indicated ALS and four indicated progressive supranuclear palsy which is not a subtype of MND.^32,33^

A 5-fold MND mortality risk was observed for female laborers based on 5 observed vs. 1.0 expected deaths (SDC Table 9). Four of the five decedents worked in chemical operations (not shown in SDC Table 9). All five death certificate diagnoses stated ALS and one certificate indicated military service.

### Breast Cancer

Analysis by race (white/non-white) showed an SMR of 1.29 for breast cancer which just reached statistical significance (95% CI 1.02 to 1.61) among non-white women, based on 76 observed deaths (not shown in Table 2). Analysis by time period showed similar statistical findings for those employed for 10 to 19 years (SMR 1.55, 95% CI 1.01 to 2.27, 26 observed deaths) and those with a 10 to 19 year latency (SMR 1.53, 95% CI 1.04 to 2.16, 29 observed deaths) (not shown in Table 2). This risk was concentrated in corporate global services operations (SMR 2.12, 95% CI 1.30 to 3.27) based on 20 observed deaths (not shown in SDC Table 7).

#### Malignant Mesothelioma

For malignant mesothelioma, women had 3 observed vs. 3.9 expected deaths (Table 2). Hire periods included the 1940s, 50s and 70s. All decedents were employed for 20 or more years and had latency periods of greater than 30 years. Only one decedent was classified with a potential manufacturing job title (operator).

#### Other Findings

In the chemicals operation, mortality risk was increased for emphysema (SMR 2.07, 95% CI 1.13 to 3.48) based on 14 observed vs. 6.8 expected deaths (SDC Table 8), mainly white women. Risk was increased for female technicians (SMR 3.74, 95% CI 1.37 to 8.14) based on 6 observed deaths.

In the coal and minerals operation, the category of LH cancers was significant (SMR 2.89, 95% CI 1.25 to 5.69, 8 observed vs. 2.8 expected deaths) (SDC Table 7). Multiple myeloma accounted for 50% of the observed LH deaths.

Female skilled craftsmen had a significant increased risk for all external causes of death (SMR 2.06, 95% CI 1.13 to 3.46) (SDC Table 9). Five of these 14 observed deaths were MVA. MVA risk was increased for female operators (SMR 2.13, 95% CI 1.10 to 3.73) (SDC Table 9).

## DISCUSSION

### Study Strengths and Limitations

This study updates the mortality experience of the original U.S.-based ExxonMobil, petrochemical refining cohort, studied through 2000.^4,5^ This 10-year update provides nearly 4.5 million person-years of observation. The number of deaths observed in the cohort (*n* = 31,091) has nearly doubled. These larger numbers aid in assessing the mortality trends of this cohort. Among the decedents, 64% are retirees, which is consistent with occupational cohorts.^34^ This study also documents changes in workforce demographics. This includes the increase in women working in manufacturing-type jobs and the increase in workers staying employed beyond retirement age (23% observed in this study).

We achieved 95% vital status ascertainment which is favorable for a large occupational cohort.^34^ For the 5% LTF, about 75% were employed less than 9 years. About 60% were classified as professionals and office/clericals, for both the first and last job. Slightly more than half of the LTF are women.

We ascertained death certificates for nearly all decedents. Less than 1% of certificates were unattainable from the state vital statistics offices. For these, we used NDI *Plus* cause of death codes. Since all of the NDI *Plus* coded causes of death were 10th revision deaths, we are reasonably confident we did not miss malignant mesothelioma deaths from the earlier period (ie, ICD-9th revision deaths), which would require manual review of the death certificate according to our standard practice.^27^ Coding was performed according to NCHS procedures to ensure consistency. Overall, we obtained specific causes of death for 98% of cohort decedents.

Traditional SMRs (observed/expected deaths) were calculated using a modified life table approach, as was done in the original study. This included using national mortality rates to compare to mortality rates in the cohort for the variety of mortality outcomes we studied (SDC Table 11). We believe using national mortality rates provides stability in the expected numbers of death for this large surveillance study. To further ensure stability of the risk measure, we only calculated SMRs when there were at least five observed or expected deaths.

This cohort is comprised of younger and older workers. A strong healthy worker effect (HWE), especially among younger workers, likely explains the 20% to 47% lower SMRs for all causes of death combined, cardiovascular disease, diabetes and nonmalignant respiratory diseases. We also observed a diminishing HWE for some cancers and with longer durations of employment, which is also common in occupational cohorts, especially among older aged workers. Because of the HWE, we do not dismiss weaker SMR findings, that is, those that are near or above unity and lack statistical significance but also recognize that findings based on smaller sample sizes are more prone to chance error.

The SMR analyses controlled for age, gender, and time period which are important variables in disease mortality. However, the study lacks information on the other mortality risk factors such as, lifestyle behaviors including smoking, exposures from other employment or nonwork-related activities, and genetics. This limits our ability to interpret weak SMRs.

The cohort was constructed from an established and stable computerized employee health surveillance system. This ensures the completeness of demographic and work history records. However, our reliance on the use of surrogates of exposure (eg, duration of employment, operating segment and job titles) constrained our ability to quantify potential exposures to specific workplace agents. Currently, we do not have a systematic mechanism to link the cohort to exposure sampling data for specific chemical exposures. We conducted an initial investigation to reconstruct benzene exposure for U.S.-based ExxonMobil refinery workers.^35^ However, additional work is needed to improve and complete the categorization of work exposures for the entire cohort population and options are being considered.

We also note that we do not have statistics on the use of personal protective equipment (PPE) that would be regularly worn by cohort members performing potentially high exposure tasks. As such, we cannot quantify the contribution of PPE in this SMR analysis.

### Mortality Results

#### Malignant Mesothelioma (Aka “Mesothelioma”)

The SMR for mesothelioma among cohort men (SMR 1.61, 95% CI 1.34 to 1.92) is similar to that reported in the original study (SMR 1.49, 95% CI 1.15 to 1.90), based on a nearly doubling of both observed and expected deaths since the original study. Mortality risk continues to be concentrated in downstream operations (SMR 2.62) where higher asbestos exposures are more likely to have occurred and among craftsmen (SMR 2.90) and managers/supervisors (SMR 1.88), whose early careers may have included manufacturing or field-based assignments. This is also evident when we examine SMRs in the update period of 2001 through 2010: downstream (SMR 2.91), craftsmen (SMR 3.70), and managers/supervisors (SMR 2.00) (SDC Table 10). By period of hire, mesothelioma mortality risk is highest for the 1940s decade of hire.

For women, 3 observed vs. 3.9 expected mesothelioma deaths were found in the update period (2001 through 2010) compared to zero cases in the original study. Only one of the three decedents was classified with a potential manufacturing job. A recent case series of men and women, diagnosed with malignant mesothelioma, without occupational exposure to asbestos, suggested that asbestos-contaminated talcum powder can cause mesothelioma.^36^ We do not have data to evaluate talcum powder usage as a potential risk factor for mesothelioma in this study.

#### Asbestosis

Asbestosis mortality risk is statistically significantly increased in this study (SMR 1.97, 95% CI 1.30 to 2.87) compared to the original study (SMR 1.88, 95% CI 0.90 to 3.46) based on 27 observed deaths (SDC Figure 11). Similar to malignant mesothelioma, risks are highest in downstream operations (SMR 2.97, 95% CI 1.76 to 4.70) and are evident when we examine the deaths from the 2001 through 2010 update period (SDC Figure 11). Most deaths (24/27) occurred among men hired in the 1940s and 1950s.

#### Asbestos-related Disease Latency/Follow-Up

Based on a 40-year latency period,^37^ it is reasonable that most of the potential malignant mesothelioma and asbestosis deaths from the 1940, 1950, and 1960 decades of hire have been ascertained in this study update (ie, 40-year latency added to 1969 = 2009 mortality tracing period). The data show mortality risk continues to be highest in the 1940s decade of hire. This increases confidence regarding the earlier work era relationship for these asbestos-related disease risks.

#### Malignant Melanoma

Malignant melanoma mortality risk has significantly increased in both men and women in this cohort. The increased risk is evident in the 2001 through 2010 update period (SMR 1.25, 95% CI 1.03 to 1.50_men_ and SMR 1.95, 95% CI 1.272.88_women_) compared to the original study (SMR 1.10, 95% CI 0.90 to 1.32_men_ and SMR 0.98, 95% CI 0.52 to 1.67_women_) as shown in SDC Figure 11 and Figure 12. Decedents are mainly white, which is a risk factor for melanoma.^38^ Increased mortality risk tends to be concentrated among nonmanufacturing operations (corporate global services) and white-collar jobs (office/clericals and professionals). For men, the SMR was significantly increased among those hired before 1960 (SMR 1.39, 95% CI 1.13 to 1.68). Better access to medical care for employees compared to the general population, may be a factor in increased diagnosis, but we do not have the data to investigate this possibility.

#### Motor Neuron Disease (MND) Including ALS and Other MNDs

We found the HWE to be less evident for the category of nervous system disease in the total cohort (SMR 0.91, 95% CI 0.85 to 0.96), primarily attributed to MND. MND mortality risks were significantly increased for both men and women in this study compared to the original study and SMRs remain significantly increased for the update period (2001 to 2010) for both genders (SDC Figure 11, SDC Figure 12).

However, for men working in upstream operations, a non-significantly increased mortality risk for MND-ALS was reported in the original study (SMR 1.47, 95% CI 0.78 to 2.51) based on 13 observed deaths.^4^ In this update, the SMR (1.28) for MND has decreased for this operating segment and continues to be non-statistically significant (95% CI 0.81 to 1.92).

In the absence of a unique ICD code for ALS, we used ICD-9 code 335.2 and ICD-10 code G12.2, which also include other MNDs. We found 82% (*n* = 125) of the death certificates (DCs) included the terms ALS or Lou Gehrig's disease, which we considered predictive of ALS, albeit not neurologically confirmed. This proportion in the MND class of diseases is consistent with the literature.^32^ About 2% (*n* = 3) of DCs indicated other MNDs such as bulbar palsy, primary lateral sclerosis and progressive muscular atrophy. For 11% (*n* = 17) of the DCs, progressive supranuclear palsy (*n* = 17), also known as Steele-Richardson Oslzewski syndrome^39^ was indicated. This is not a MND sub-type^32,40^ and has a separate ICD-10 code of G23.1.^41^ The remaining 5% (*n* = 7) of certificates indicated the nonspecific term of motor neuron disease. While some physicians may use this term interchangeably with ALS,^42^ we found it may not always translate into ALS. For example, the MND term was next to “Kennedy's syndrome,” which is a lower motor neuron, X-linked recessive disease, not ALS.^43,44^ Accuracy of ALS coding is expected to improve with the implementation of ICD-11 codes, which provide unique codes for ALS and each of the other MNDs.^45^

To the best of our knowledge, ALS and the other MNDs have not been well-studied in other petroleum industry cohorts. A Canadian petroleum cohort found a non-significant moderate excess of ALS, based on 15 observed deaths and 8.8 expected deaths (SMR 1.70, 95% CI 0.95 to 2.80). Ten of the Canadian cohort deaths were observed in non-exposed workers which resulted in a statistically significant risk (SMR 2.48, 95% CI 1.19 to 4.56).^6^

Possible links between ALS and excessive physical work (involving occupations in farming, construction and professional sports), work with pesticides, and possibly work involving lead, electromagnetic fields and health care work have been reported.^46^ Another study reported higher proportionate mortality ratios for ALS mortality in occupations with higher SES such as occupational categories of computer and mathematics and architecture and engineering.^47^ An association between higher latitudes and increased ALS mortality rates has also been reported.^32^ We do not have sufficient data in our cohort to consider the potential influence of these reported exposures and associations.

The U.S. Department of Veteran Affairs considers ALS to be a service-connected disease.^48^ Data we collect are also insufficient to assess the potential influence from military service on the risk of ALS mortality in this cohort. Review of death certificates for mention of military service found 45% indicated “yes,” 44% indicated “no” and 12% indicated “unknown” or were blank.

Lastly, ALS is considered to be a disease of an aging population and as such prevalence is expected to increase over the next 20 years.^47,49^ In this study, 75% of the MND cohort cases died at or above age 60 years. We also note that 68% of the deaths occurred in retirees and a few died while employed. The cohort's access to health care benefits and higher SES,^47^ compared to the general population, may have contributed to the MND diagnosis, but we do not have the data to illustrate this possibility. Newer ICD coding and possible linkage to the National ALS Registry^50,51^ can potentially improve ALS surveillance.

#### Brain Cancer

Brain cancer mortality risk is significantly increased among men in the upstream operation (SMR 1.36, 95% CI 1.05 to 1.75) based on 63 observed deaths. Mortality risk is concentrated among male manager/supervisors in this operating segment (SMR 1.82, 95% CI 1.11 to 2.81, 20 observed deaths). These analyses were not reported in the original study for direct comparison.

For the overall subgroup of male manager/supervisors, the SMRs for brain cancer are slightly increased but not statistically significant when we examine the three study periods: SMR 1.11, 95% CI 0.80 to 1.50, based on 39 observed deaths (1979 to 2000), SMR 1.35, 95% CI 0.92 to 1.93, based on 28 observed deaths (2001 to 2010) and SMR 1.20, 95% CI 0.93 to 1.52 based on 67 observed deaths (1979 to 2010).

Conversely, we found decreasing mortality trends observed for brain cancer among male office/clericals. In the original study, a non-significant increased SMR for brain cancer was reported for male office/clericals (SMR 1.39, 95% CI 95% CI 0.61 to 1.75, 7 observed deaths). The SMR for this subgroup has decreased to 0.86 (95% CI 0.35 to 1.78) with no additional brain cancer deaths observed in this group for the 2001 through 2010 update period.

To date, the etiology of brain cancer is largely unknown. BP Amoco detected an excess of brain cancer incidence among white men employed 10 or more years, in one building of a petrochemical research facility. The findings were based on small numbers, 6 observed cases vs. 2.0 expected cases (standardized incidence ratio or SIR 3.02, 95% CI 1.11 to 6.57). No specific chemical exposure was identified with this reported risk.^52^

In this study 75% of the upstream brain cancer decedents are retirees. It is possible that health care benefits for these decedents may have influenced a diagnosis compared to the general population.

#### Bladder Cancer: Women

Bladder cancer in women was significantly increased in this study (SMR 1.55, 95% CI 1.04 to 2.23, 29 observed deaths) compared to the original study (SMR 1.74, 95% CI 0.90 to 3.04, 12 observed deaths). Most decedents worked as office/clericals and professionals and there was no notable change from first to last EEO category for these decedents. However, when we examined mortality risk of the 2001 through 2010 update period, the SMR is slightly lower and no longer significant (SMR 1.44, 95% CI 0.87 to 2.26), based on 17 observed deaths, suggesting a downward mortality trend.

Smoking is the main risk factor for bladder cancer.^53^ Occupational exposures associated with aluminum production, rubber industry, leather industry and exposure to 4-aminobiphenyl, and benzidine have been linked to bladder cancer.^54^ For this cohort, we do not have data on smoking or specific chemical exposures including exposures that may have occurred during other employment outside of this company. Review of the death certificate data for tobacco use was uninformative.

#### Breast Cancer: Women, Non-White Office/Clericals

Mortality from breast cancer was significantly increased among non-white cohort women (SMR 1.29, 95% CI 1.02 to 1.61). Nearly 62% of the decedents worked as office/clericals (SMR 1.38, 95% CI 1.02 to 1.84).

Breast cancer is considered the most common cancer in women in the U.S. following skin cancer.^55^ Risk factors include genetics, aging, reproductive and family histories, hormones and possibly changes in hormones due to night shift working.^55,56^ Since most decedents worked in office/clerical jobs, the shift work hypothesis seems less plausible for this cohort finding. We do not have medical or genetic data for the cohort. Access to health care benefits may have contributed to the breast cancer diagnosis of women in this cohort.

#### Ovarian Cancer: Women Office/Clericals

For ovarian cancer, significant increased risk was observed among white office/clerical workers (SMR 1.37, 95% CI 1.09 to 1.70, 82 observed deaths), who worked in corporate global services (SMR 1.78, 95% CI 1.04 to 2.85, 17 observed deaths). In the original study, women working as office/clericals had a similar SMR finding (SMR 1.40, 95% CI 1.02 to 1.87) but results by each operating segment were not reported. For the update period (2001 to 2010), the SMR for ovarian cancer in office/clericals decreased and is no longer statistically significant (SMR 1.18, 95% CI 0.85 to 1.60) based on 39 observed deaths.

Ovarian cancer is often associated with family history, genetic mutations and hormonal factors.^57^ Asbestosis is classified by IARC as having sufficient evidence to cause ovarian cancer in humans^58^ and limited evidence for associations with silica dust, diesel exhaust, and organic solvents.^59^ We did not find significantly increased SMRs for ovarian cancer among women in potentially higher asbestos jobs such as downstream operations or as skilled craftsmen. Better access to medical care for employees compared to the general population, may be a factor, but we do not have the data to investigate this possibility.

#### Motor Vehicle Accidents: Male Operators and Female Laborers

Significant mortality risk from MVA is observed among cohort members working as operators, both men (SMR 1.28, 95% CI 1.08 to 1.50) and women (SMR 2.13, 95% CI 1.10 to 3.73, 12 observed deaths). When we examine the update period (2001 to 2010), the risk for cohort men significantly increases (SMR 1.56, 95% CI 1.15 to 2.08) but is no longer evident for female operators based on 1 observed vs. 1.3 expected deaths, as compared to the original study (SMR 1.19, 95% CI 0.98 to 1.44_men_ and SMR 2.56, 95% CI 1.34 to 4.45_women_).

#### Decreasing Mortality Findings

##### MEN

We found decreasing mortality trends for a few causes of death among men in the cohort (see SDC Figure 11).

###### Aplastic Anemia

The mortality risk for aplastic anemia among downstream men (SMR 1.56, 95% CI 0.75 to 2.86) appears to be decreasing compared to the original study (SMR 2.19, 95% CI 0.95 to 4.32). For the 2001 through 2010 update period, there are only 2 observed and 2.7 expected deaths from aplastic anemia.

We acknowledge the potential for misclassification of disease with death certificate cause of death diagnoses. This is known to occur for aplastic anemia with pancytopenia^60^ and with distinguishing between specific leukemia subtypes and those classified as unspecified. This may or may not affect the SMRs, depending on possible differences in death certificate diagnostic accuracy for cohort members versus the general population. We expect a minimal effect due to consistency in death certificate coding.

###### Other Specified Diseases of Blood/Blood-Forming Organs Including MDS

Mortality risk is no longer statistically significantly increased in men in upstream operations (SMR 1.02, 95% CI 0.58 to 1.66) based on 16 observed deaths compared to the original study (SMR 2.28, 95% CI 1.27 to 2.28, 13 observed deaths). Only three observed and 10.0 expected deaths were identified in the update period (2001 to 2010) resulting in an SMR of 0.30 (95% CI 0.08 to 0.82). For men in downstream operations, the SMR (1.23, 95% CI 0.90 to 1.65) is also decreased compared to the original study (SMR 2.18, 95% CI 0.80 to 4.75) and is decreased in the update period (SMR 1.15, 95% CI 0.83 to 1.56).

###### ANLL

Mortality risk among men in the chemicals operation appears to be decreasing. In the original study, a significant increase was observed (SMR 1.81, 95% CI 1.06 to 2.90) based on 17 observed deaths. The SMR has decreased to 1.25 and is no longer statistically significant (95% CI 0.82 to 1.84) based on 26 observed deaths. Risk decreased 36% when we evaluated the 2001 through 2010 period (SMR 0.80, 95% CI 0.39 to 1.46) (see SDC Figure 13).

###### Multiple Myeloma

In the original study, there were 5 observed vs. 2.1 expected multiple myeloma deaths among men in the coal and minerals operation (SMR 2.38, 95% CI 0.87 to 5.28). In this study, mortality risk has decreased (SMR 1.81, 95% CI 0.78 to 3.56) based on 8 observed and 4.4 expected deaths in this group of men. There are only 3 observed deaths vs. 2.3 expected deaths observed in the update period (2002–2010).

##### WOMEN

For women, decreasing mortality trends are noted for several causes of death compared to the original study (1979 to 2000) (see SDC Figure 12).

###### Emphysema (Professionals)

The emphysema mortality risk for female professionals, suspected in the original study (SMR 1.87, 95% CI 0.75 to 3.85, 7 observed) has not significantly increased in the total observation period (1979 to 2010) (SMR 1.19, 95% CI 0.54 to 2.26, 9 observed deaths) and has decreased in the 2001 through 2010 update period, with only two observed deaths.

###### Lung Cancer (Operators)

Lung cancer mortality risk among female operators appears to be decreasing with 16 of the 30 observed deaths resulting in a non-significantly increased SMR of 1.30 (95% CI 0.77 to 2.07) in the update period (2001–2010).

###### Cerebrovascular Disease (Laborers)

In the original study (1979–2000) the SMR for cerebrovascular disease among female laborers was significantly increased (SMR 2.04, 95% CI 1.14 to 3.36). The SMRs have decreased and are no longer significant in both the total observation period (SMR 1.33, 05% CI 0.85 to 1.98, 24 observed deaths) and in the update period (2001 to 2010) (SMR 0.85, 95% CI 0.41 to 1.56, 9 observed deaths).

###### Motor Vehicle Accidents (Operators, Coal and Minerals Operation)

MVA mortality risk was significantly increased in the original study for women working as operators (SMR 2.56, 95% CI 1.34 to 4.44) and among women working in coal and minerals (SMR 3.13, 1.14 to 6.93, 5 observed deaths). Risk has decreased when we examine the update period (2001 to 2010) in female operators (1 observed vs. 1.3 expected deaths) and women in coal and minerals operations (0 observed vs. 0.4 expected deaths).

## CONCLUSION

This study found mortality risk for malignant mesothelioma and asbestosis to be highest among men hired in the 1940s. In both men and women, statistically significant increases in mortality were observed for malignant melanoma and motor neuron disease with no clear work-related patterns. For melanoma, excess risk was associated with non-manufacturing operations and white-collar jobs. For MND, deaths occurred across operating and job titles studied.

Subgroup analyses (by operating segment and job title) found a 1.4-fold increase in brain cancer among upstream men with a higher risk among managers/supervisors (1.8-fold). Conversely, we found no evidence of increasing brain cancer risk among office/clericals (SMR 0.86, 95% CI 0.35 to 1.78) which was suggested in the original study (SMR 1.39, 95% CI 0.61 to 1.75). Significantly increased mortality risk from MVA were concentrated among male operators and laborers. Female laborers also had increased risk of MVA but based on smaller numbers.

Overall, women generally had higher mortality risk than men. The all-cause SMRs were above unity for female skilled craftsmen (SMR 1.08, 95% CI 0.86 to 1.33), operators (SMR 1.11, 95% CI 0.98 to 1.25) and laborers (SMR 1.22, 95% CI 1.11 to 1.35). This may be suggestive of possible differences in lifestyle between genders.

The study also showed mortality risks are decreasing for aplastic anemia, other diseases of blood/blood-forming organs including MDS, ANLL, multiple myeloma and bladder cancer.

### Clinical Significance

The results of this mortality study provide information regarding the potential chronic health patterns observed among a cohort of U.S.-based petrochemical and refinery workers compared to the U.S. general population. This information can be used to inform those responsible for managing exposure controls, employee health programs and regulations/policy.

## Supplementary Material

Supplemental Digital Content

## Supplementary Material

Supplemental Digital Content

## Supplementary Material

Supplemental Digital Content

## Supplementary Material

Supplemental Digital Content

## Supplementary Material

Supplemental Digital Content

## Supplementary Material

Supplemental Digital Content

## Supplementary Material

Supplemental Digital Content

## Supplementary Material

Supplemental Digital Content

## Supplementary Material

Supplemental Digital Content

## Supplementary Material

Supplemental Digital Content

## Supplementary Material

Supplemental Digital Content

## Supplementary Material

Supplemental Digital Content

## Supplementary Material

Supplemental Digital Content
