# Supplementary material for: Mortality of 196,826 Men and Women Working in U.S.-Based Petrochemical and Refinery Operations: Update 1979 to 2010
Source: J Occup Environ Med. 2021 Oct 20;64(3):250–62. doi: 10.1097/JOM.0000000000002416 (PMC8887844; doi:10.1097/JOM.0000000000002416)
Supplement: Supplemental Digital Content [file joem-64-0250-s001.docx]

Supplemental Digital Content 1, Table Listing Study Causes of Death and Respective ICD-9 and ICD-10 Codes

| **CAUSE(S) OF DEATH** | **REVISION 9 ICD CODES** | **REVISION 10 ICD CODES** |
| --- | --- | --- |
| All Causes | 001-999 | A00-Y89 |
| Infectious and Parasitic Diseases | 001-139, 279.1 | A00-B99 |
| Tuberculosis | 010-018 | A15-A19 |
| Human Immunodeficiency Virus (HIV) Disease (incl. AIDS) | 042-044, 279.1 | B20-B24 |
| Malignant Neoplasms (MNs) | 140-208 | C00-C97 |
| MN of Buccal Cavity and Pharynx | 140-149 | C00-C14 |
| MN of Pharynx | 146-149 | C09-C14 |
| MN of Digestive Organs and Peritoneum | 150-159 | C15-C26, C48 |
| MN of Esophagus | 150 | C15 |
| MN of Stomach | 151 | C16 |
| MN of Large Intestine (Colon) | 153 | C18 |
| MN of Rectum | 154 | C20-C21 |
| MN of Biliary Passages (including Gallbladder)/Liver | 155, 156 | C22-C24 |
| MN of Liver (Specified Primary or Unspecified) | 155.0, 155.2 | C22.0, C22.2-C22.9 |
| MN of Pancreas | 157 | C25 |
| MN of Respiratory System | 160-165 | C30-C39 |
| MN of Nasal Cavity/Mid Ear/Accessory Sinuses | 160 | C30-C31 |
| MN of Larynx | 161 | C32 |
| MN of Bronchus, Trachea, Lung | 162 | C33-C34 |
| MN of Bone | 170 | C40-C41 |
| MN of Connective Tissue | 171 | C49 |
| MN of Skin | 172-173 | C43-C44 |
| Malignant Melanoma | 172 | C43 |
| Malignant Mesothelioma | See * | C45 |
| MN of Breast | 174-175 | C50 |
| MN of Cervix Uteri | 180 | C53 |
| MN of Body of Uterus (including Corpus Uteri) | 182 | C54 |
| MN of Ovary | 183.0 | C56 |
| MN of Prostate | 185 | C61 |
| MN of Testicular | 186 | C62 |
| MN of Bladder and Other Urinary | 188, 189.3-189.4, 189.8-189.9 | C67-C68 |
| MN of Bladder (Monson) | 188 | C67 |
| MN of Kidney | 189.0-189.2 | C64-C66 |
| MN of Central Nervous System (CNS) including Brain | 191-192 | C70-C72 |
| MN of Brain | 191 | C71 |
| MN of Other/Ill-Defined Sites/Secondary Neoplasms | 195-199 | C76-C80, C97 |
| MN of Lymphatic and Hematopoietic Tissue | 200-208 | C81-C96 |
| Hodgkin Lymphoma | 201 | C81 |
| Non Hodgkin Lymphoma | 200.0-200.2, 202.0, 202.8 | C82-C83, C85 |
| Nodular/Follicular Lymphoma | 202.0 | C82.9 |
| Reticulosarcoma | 200.0 | C83.3 |
| T-Cell Lymphoid Variety | 202.1-202.2 | C84.0-C84.1 |
| Lymphosarcoma | 200.1 | C85.0 |
| Other Lymphomas | 202.8 | C85.9 |
| Multiple Myeloma | 203.0 | C90.0 |
| Leukemia & Aleukemia | 204-208 | C91-C95 |
| Acute Lymphocytic Leukemia (ALL) | 204.0 | C91.0 |
| Chronic Lymphocytic Leukemia (CLL) | 204.1 | C91.1 |
| Hairy Cell Leukemia | 202.4 | C91.4 |
| Acute Myelocytic Leukemia (AML) | 205.0 | C92.0 |
| Chronic Myelocytic Leukemia (CML) | 205.1 | C92.1 |
| Acute Monocytic Leukemia | 206.0 | C93.0 |
| Chronic Monocytic Leukemia | 206.1 | C93.1 |
| Acute Erythremia and Erythroleukemia | 207.0 | C94.0 |
| Megakaryocytic Leukemia | 207.2 | C94.2 |
| Acute Non-Lymphocytic Leukemia (ANLL) | 205.0, 206.0, 207.0, 207.2 | C92.0, C93.0, C94.0, C94.2 |
| Other/Unspecified Leukemia (besides ANLL, CML, ALL, CLL) | 204.2-204.9, 205.2-205.9, 206.1-206.9, 207.1, 207.8, 208 | C91.2-C91.9, C92.2-C92.9, C93.1-C93.9, C94.1, C94.3-C94.7, C95 |
| Benign/CIS/Uncertain Behavior/Unspecified Neoplasms | 210-239 | D00-D48 |
| Benign CNS (including Brain) | 225 | D33 |
| Benign Brain | 225.0 | D33.0-D33.2 |
| Uncertain Behavior/Unspecified - Brain/Spinal Cord | 237.5, 239.6 | D43.0-D43.2, D43.4 |
| All Diseases of Blood and Blood-Forming Organs | 280-289 | D50-D89 |
| Aplastic Anemia | 284 | D60-D61 |
| All Other Anemias | 280-283, 285 | D50-D59, D62-D64 |
| All Other Diseases of Blood-Forming Organs | 288-289 | D70-D89 |
| Other Spec Diseases of Blood/Blood-Form Org (including MDS) | 289.8 | D46, D47.1, D75.8 |
| Endocrine/Nutritional/Metabolic Diseases | 240-279 | E00-E90 |
| Diabetes Mellitus | 250 | E10-E14 |
| Mental Disorders | 290-319 | F00-F99 |
| Alcoholism | 303 | F10 |
| Drug Psychosis, Dependence, Poisoning | 292, 304, 305.2-305.9, E850-E858 | F11-F19, X40-X44, X46-X49 |
| Nervous System/Sense Organ Disease | 320-389 | G00-H95 |
| Parkinson's Disease | 332 | G20 |
| Motor Neuron Disease including Amyotrophic Lateral Sclerosis | 335.2 | G12.2 |
| Multiple Sclerosis | 340 | G35 |
| Circulatory Disease | 390-459 | I00-I99 |
| All Heart Disease | 391-398, 402, 404, 410-429 | I01-I09, I11, I13, I20-I52 |
| Hypertension with Heart Disease | 402, 404 | I11, I13 |
| Ischemic Heart Disease | 410-414 | I20-I25 |
| Acute Myocardial Infarction | 410 | I21 |
| Hypertension without Heart Disease | 401, 403, 405 | I10, I12, I15 |
| Cerebrovascular Disease | 430-438 | I60-I69 |
| Diseases of Arteries/Veins/Other Circulatory | 440-459 | I70-I99 |
| Aortic Aneurysm | 441 | I71 |
| Non-Malignant Respiratory Disease | 460-519 | J00-J99 |
| Acute Respiratory Infections except Influenza/ Pneumonia | 460-466 | J00-J06, J20-J22 |
| Pneumonia | 480-486 | J12-J18 |
| Influenza | 487 | J09-J11 |
| Bronchitis, Emphysema, and Asthma | 490-493 | J40-J43, J45-J46 |
| Bronchitis | 490, 491 | J40-J42 |
| Emphysema | 492 | J43 |
| Asthma | 493 | J45-J46 |
| Pneumoconiosis and Other Respiratory Diseases | 470-478, 494-519 | J30-J39, J44, J47-J99 |
| Chronic Obstructive Pulmonary Disease | 496 | J44 |
| Pneumoconiosis/Other Lung Diseases, External Agents | 500-508 | J60-J70 |
| Asbestosis | 501 | J61 |
| Silicosis and Anthracosilicosis | 500, 502 | J60, J62 |
| Digestive Disease | 520-579 | K00-K93 |
| Ulcer of Stomach and Duodenum | 531-533 | K25-K27 |
| Cirrhosis of Liver | 571 | K70, K74 |
| Genitourinary Disease | 580-629 | N00-N99 |
| Nephritis and Nephrosis | 580-589 | N00-N29 |
| Skin/Subcutaneous Tissue Disease | 680-709 | L00-L99 |
| Musculoskeletal Disease & Connective Tissue | 710-739 | M00-M99 |
| All External Causes of Death | E800-E999 | V01-Y89 |
| Accidents | E800-E949 | V01-X59, Y85-Y86 |
| Transportation Accidents | E800-E848, E929.0-E929.1 | V01-V99, Y85 |
| Motor Vehicle Accidents (MVA) | E810-E825 | V02-V05, V09, V12-V15, V18-V79 |
| All Other Accidents besides MVA | E800-E807, E826-E949 | V80-V99, W00-X59 |
| Suicides | E950-E959 | X60-X84 |
| Homicides and Legal Intervention | E960-E978 | X85-Y09, Y35 |
| Congenital Anomalies | 740-759 | Q00-Q99 |

ICD, International Statistical Classification of Diseases

MDS, Myelodysplastic Syndrome

* Wojcik N, Schnatter A, Huebner W. Mesothelioma in occupational cohort studies. Methodological considerations. J Occup Environ Med. 2014;56:47-51.
