# Supplementary material for: Mortality of 196,826 Men and Women Working in U.S.-Based Petrochemical and Refinery Operations: Update 1979 to 2010
Source: J Occup Environ Med. 2021 Oct 20;64(3):250–62. doi: 10.1097/JOM.0000000000002416 (PMC8887844; doi:10.1097/JOM.0000000000002416)
Supplement: Supplemental Digital Content [file joem-64-0250-s002.docx]

Supplemental Digital Content 10, Table Summarizing Malignant Mesothelioma Mortality in Men, SMR (95% CI), Observed/Expected Deaths, by Selected Work Factors and Study Period.

| **Work Factors** | **Original Study**  **1979-2000**  **(64 observed deaths)** | **Update Period**  **2001-2010**  **(56 observed deaths)** | **Total Observation Period**  **1979-2010**  **(120 observed deaths)** |
| --- | --- | --- | --- |
|  | **SMR (95% CI) Obs/Exp▪** | **SMR (95% CI) Obs/Exp▪** | **SMR (95% CI) Obs/Exp▪** |
| **Period of Hire** |  |  |  |
| <1940 | -- 2/1.2 | -- 1/0.4 | -- 3/1.6 |
| 1940-1949 | 1.90 (1.28-2.73)** 29/15.2 | 3.38 (2.20-5.00)** 23/6.8 | 2.36 (1.78-3.08)** 52/22.0 |
| 1950-1959 | 1.60 (0.95-2.53) 18/11.2 | 1.53 (0.85-2.55) 13/8.5 | 1.57 (1.09-2.21)* 31/19.7 |
| 1960-1969 | 1.12 (0.45-2.30) 7/6.3 | 1.64 (0.89-2.80) 12/7.3 | 1.40 (0.87-2.14) 19/13.6 |
| 1970-1979 | 1.10 (0.40-2.39) 6/5.5 | 0.96 (0.45-1.83) 8/8.3 | 1.01 (0.58-1.66) 14/13.8 |
| 1980-1989 | -- 2/3.6 | -- 1/3.2 | 0.44 (0.11-1.20) 3/6.8 |
| 1990-1999 | -- 0/0 | -- 1/0.9 | -- 1/0.9 |
| 2000-2010 | -- 0/0 | -- 0/0.1 | -- 0/0.1 |
| **Age of Hire** |  |  |  |
| <25 | 2.18 (1.53-3.01)** 34/15.6 | 2.12 (1.36-3.15)** 22/10.4 | 2.14 (1.64-2.78) 5/26.0 |
| 25-29 | 1.20 (0.67-1.98) 15/12.5 | 3.45 (2.37-4.86)** 30/8.7 | 1.42(0.97-2.00) 30/21.2 |
| 30+ | 1.00 (0.56-1.65) 15/15.0 | 1.54 (0.96-2.37) 19/12.3 | 1.24 (0.88-1.72) 34/27.3 |
| **Duration (Years of Employment)** |  |  |  |
| 0-9 | 0.60 (0.12-1.76) 3/5.0 | 3/4.0 | 0.78 (0.32-1.62) 6/7.7 |
| 10-19 | 1.38 (0.60-2.72) 8/5.8 | 1.11 (0.41-2.46) 5/4.5 | 1.26 (0.70-2.10) 13/10.3 |
| 20+ | 1.64 (1.23-2.15) 53/32.3** | 1.98 (1.47-2.60)** 48/24.2 | 1.78 (1.46-2.16)** 101/56.6 |
| **Latency (Years from First Hire to Death)** |  |  |  |
| 0-9 | -- 2/2.6 | -- 0/2.4 | -- 2/0.2 |
| 10-19 | -- 3/4.7 | -- 0/3.6 | -- 3/1.1 |
| 20+ | 1.65(1.26-2.13) 59/35.7** | 1.49(1.1-1.92)* 56/37.6 | 1.5 (1.30-1.88)** 115/73.3 |
| **Operating Segment** |  |  |  |
| Downstream | 2.44 (1.79-3.24) 47/19.3** | 2.91 (2.01-3.97)** 37/12.7 | 2.62 (2.11-3.23)**84/32.0 |
| Upstream | 0.64 (0.21-1.48) 5/7.9 | 1.05 (0.43-2.19) 6/5.7 | 0.81 (0.42-1.41) 11/13.6 |
| Chemicals | 0.70 (0.19-1.80) 4/5.7 | 1.30 (0.53-2.71) 6/4.6 | 0.97 (0.49-1.73) 10/10.3 |
| Coal and Minerals | -- 1/0.9 | -- 0/0.7 | --- 0/1.6 |
| Corporate Global Services | -- 0/2.6 | -- 2/2.2 | —2/4.8 |
| Mixed | -- 3/3.3 | -- 3/3.7 | 0.86 (0.35-1.78) 6/7.0 |
| Missing | -- 4/3.4 | -- 3/1.9 | 1.32 (0.58-2.61) 7/5.3 |
| **Job Title (EEO)** |  |  |  |
| Managers/Supervisors | 1.77 (1.07-2.77) 19/10.7* | 2.00 (1.20-3.14)* 17/8.5 | 1.88 (1.33-2.57)** 36/19.2 |
| Professionals | 1.10 (0.62-1.81) 15/13.6 | 1.13 (0.61-1.92) 12/0.6 | 1.12 (0.75-1.60) 27/24.2 |
| Skilled Craftsmen | 2.38 (1.45-3.67) 20/8.4** | 3.70 (2.33-5.62)** 20/5.4 | 2.90 (2.10-3.91)** 40/13.8** |
| Operators | -- 4/4.4 | -- 2/3.2 | 0.79 (0.32-1.64) 6/7.6 |
| Technicians | -- 4/2.6 | —5/2.1 | 1.92 (0.93-3.51) 9/4.7 |
| All Other (Sales, Office/Clericals, Laborers, Service, Missing) | -- 2/3.3 | -- 1/0.8 | —2/4.1 |

SMR (95% CI), standardized mortality ratio (95% confidence interval).

Obs/Exp, Observed deaths/Expected deaths

▪Expected deaths based on U.S. general population mortality rates.

*Statistically significant at *P* <0.05.

**Statistically significant at *P* <0.01.
