# Supplementary figures and images for: Mortality of 196,826 Men and Women Working in U.S.-Based Petrochemical and Refinery Operations: Update 1979 to 2010
Source: J Occup Environ Med. 2021 Oct 20;64(3):250–62. doi: 10.1097/JOM.0000000000002416 (PMC8887844; doi:10.1097/JOM.0000000000002416)

Supplemental Digital Content 11, Figure Comparing A Priori SMR Results by Study Period for Men


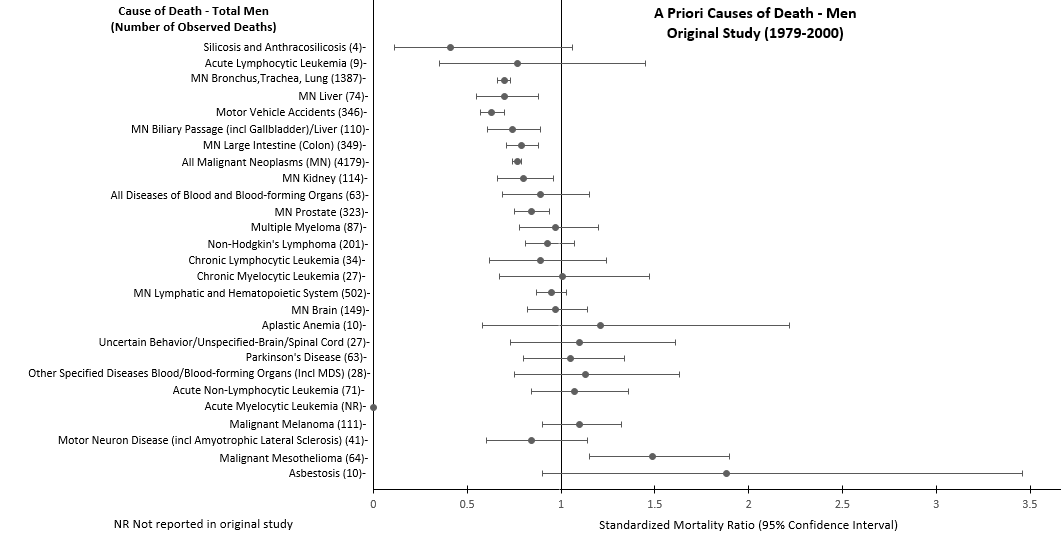


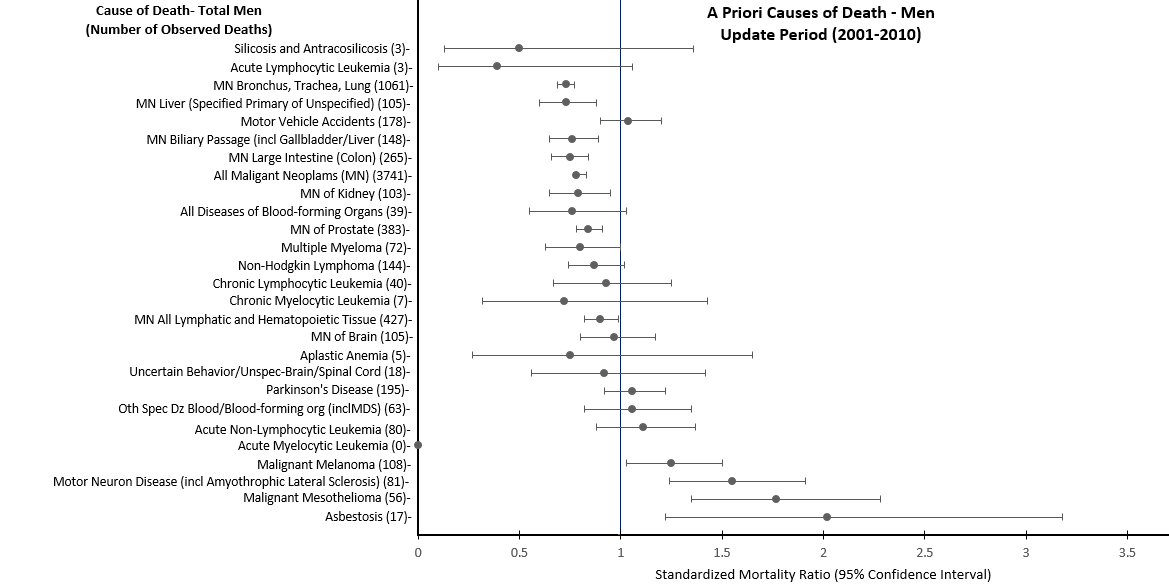


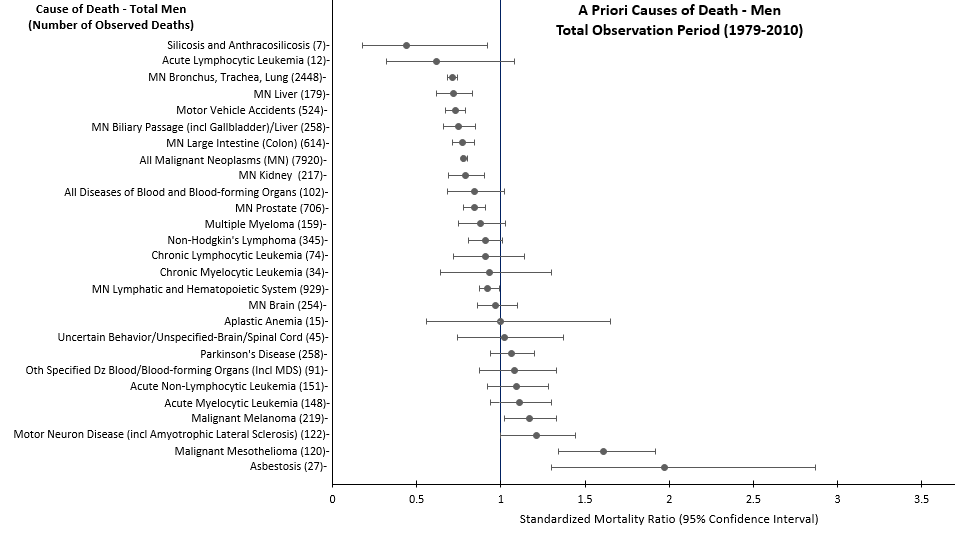

Supplement: Supplemental Digital Content [file joem-64-0250-s003.docx]

Supplemental Digital Content 12, Figure Comparing A Priori SMR Results by Study Period for Women


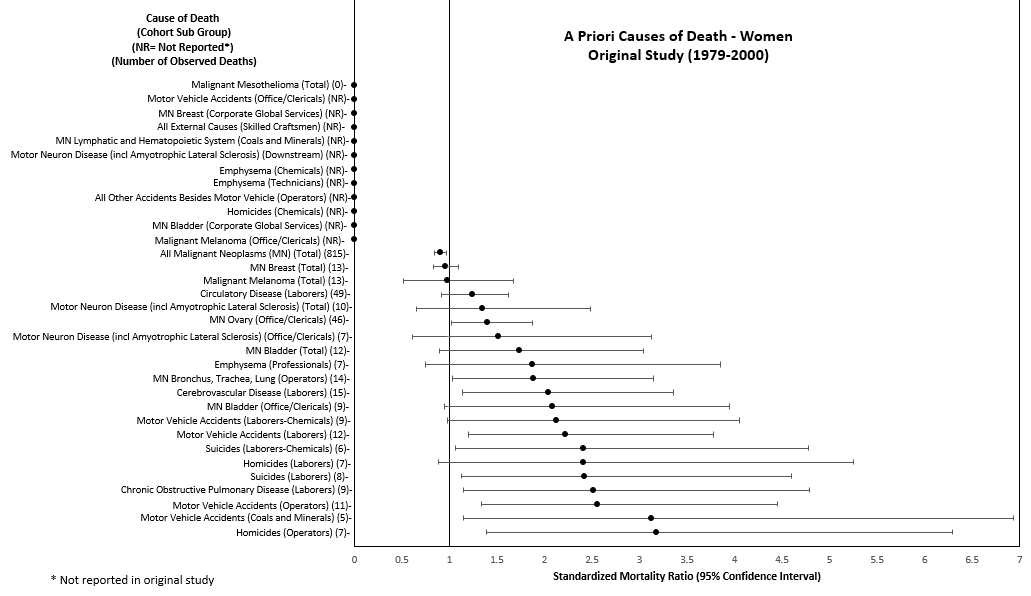


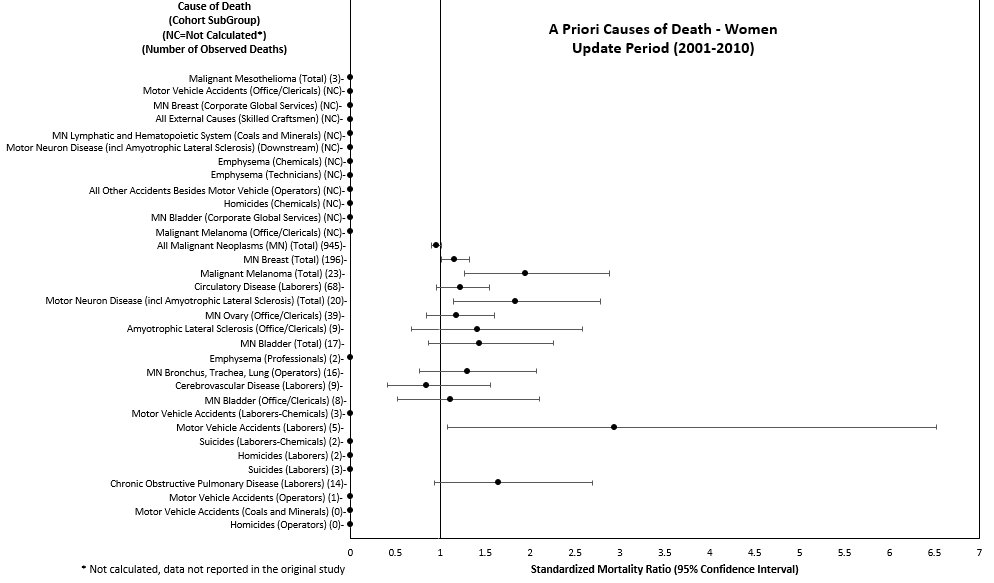


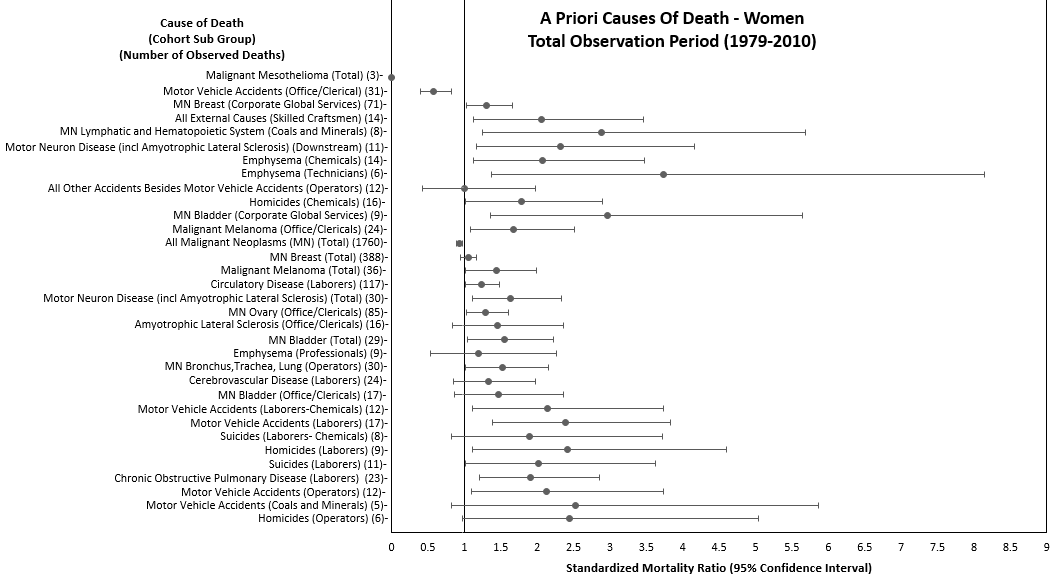

Supplement: Supplemental Digital Content [file joem-64-0250-s004.docx]
