# Supplementary material for: Mortality of 196,826 Men and Women Working in U.S.-Based Petrochemical and Refinery Operations: Update 1979 to 2010
Source: J Occup Environ Med. 2021 Oct 20;64(3):250–62. doi: 10.1097/JOM.0000000000002416 (PMC8887844; doi:10.1097/JOM.0000000000002416)
Supplement: Supplemental Digital Content [file joem-64-0250-s005.docx]

Supplemental Digital Content 13, Figure Comparing SMR Results for ANLL in Men by Operating Segment and Study Period


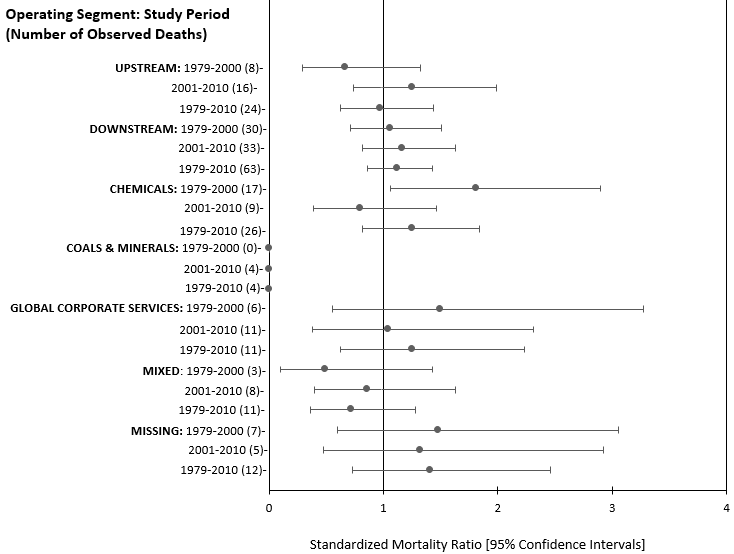


ANLL (acute non-lymphocytic leukemia), which combined acute myelocytic with acute monocytic, megakaryocytic, and acute erythremia/erythroleukemia
