# Supplementary material for: Mortality of 196,826 Men and Women Working in U.S.-Based Petrochemical and Refinery Operations: Update 1979 to 2010
Source: J Occup Environ Med. 2021 Oct 20;64(3):250–62. doi: 10.1097/JOM.0000000000002416 (PMC8887844; doi:10.1097/JOM.0000000000002416)
Supplement: Supplemental Digital Content [file joem-64-0250-s006.docx]

Supplemental Digital Content 2, Table Listing Mortality Results of U.S.-based Petroleum Cohort by Main Operating Segments (1979-2010) – MEN

| **Cause of Death** | **DOWNSTREAM** | | | **UPSTREAM** | | | **CHEMICALS** | | |
| --- | --- | --- | --- | --- | --- | --- | --- | --- | --- |
|  | **Observed** | **Expected▪** | **SMR (95% CI)** | **Observed** | **Expected▪** | **SMR (95% CI)** | **Observed** | **Expected▪** | **SMR (95% CI)** |
| All Causes | 11606 | 15732.4 | 0.74 (0.72-0.75)** | 4503 | 6717.2 | 0.67 (0.65-0.69)** | 4092 | 5589.9 | 0.73 (0.71-0.76)** |
| Infectious and Parasitic Diseases | 237 | 440.5 | 0.54 (0.47-0.61)** | 140 | 201.8 | 0.69 (0.58-0.82)** | 113 | 210.7 | 0.54 (0.44-0.64)** |
| Tuberculosis | 0 | 9.9 | - | 2 | 3.9 | - | 1 | 3.8 | - |
| Human Immunodeficiency Virus (HIV) Disease (incl. AIDS) | 65 | 170.8 | 0.38 (0.29-0.48)** | 74 | 84.5 | 0.88 (0.69-1.10) | 33 | 106.0 | 0.31 (0.21-0.44)** |
| Malignant Neoplasms (MNs) | 3521 | 4231.9 | 0.83 (0.81-0.86)** | 1357 | 1798.5 | 0.76 (0.72-0.80)** | 1178 | 1488.3 | 0.79 (0.75-0.84)** |
| MN of Buccal Cavity and Pharynx | 51 | 86.9 | 0.59 (0.44-0.77)** | 29 | 36.9 | 0.78 (0.53-1.13) | 25 | 33.4 | 0.75 (0.48-1.10) |
| MN of Pharynx | 21 | 44.9 | 0.47 (0.29-0.72)** | 17 | 19.0 | 0.89 (0.52-1.43) | 12 | 17.7 | 0.68 (0.35-1.19) |
| MN of Digestive Organs and Peritoneum | 855 | 1037.6 | 0.82 (0.77-0.88)** | 335 | 441.1 | 0.76 (0.68-0.84)** | 292 | 375.0 | 0.78 (0.69-0.87)** |
| MN of Esophagus | 103 | 139.9 | 0.74 (0.60-0.89)** | 46 | 60.0 | 0.77 (0.56-1.02) | 33 | 53.4 | 0.62 (0.42-0.87)** |
| MN of Stomach | 91 | 110.0 | 0.83 (0.67-1.02) | 34 | 45.4 | 0.75 (0.52-1.05) | 31 | 38.8 | 0.80 (0.54-1.13) |
| MN of Large Intestine (Colon) | 267 | 337.0 | 0.79 (0.70-0.89)** | 103 | 141.8 | 0.73 (0.59-0.88)** | 89 | 114.7 | 0.78 (0.62-0.95)* |
| MN of Rectum | 47 | 62.2 | 0.76 (0.56-1.01) | 13 | 26.5 | 0.49 (0.26-0.84)** | 27 | 22.4 | 1.21 (0.80-1.76) |
| MN of Biliary Passages (including Gallbladder)/Liver | 121 | 138.1 | 0.88 (0.73-1.05) | 41 | 59.8 | 0.69 (0.49-0.93)* | 37 | 54.3 | 0.68 (0.48-0.94)* |
| MN of Liver (Specified Primary or Unspecified) | 84 | 99.1 | 0.85 (0.68-1.05) | 31 | 43.1 | 0.72 (0.49-1.02) | 23 | 40.4 | 0.57 (0.36-0.85)** |
| MN of Pancreas | 198 | 219.5 | 0.90 (0.78-1.04) | 85 | 94.1 | 0.90 (0.72-1.12) | 69 | 79.8 | 0.86 (0.67-1.10) |
| MN of Respiratory System | 1119 | 1508.0 | 0.74 (0.70-0.79)** | 416 | 638.7 | 0.65 (0.59-0.72)** | 401 | 527.2 | 0.76 (0.69-0.84)** |
| MN of Nasal Cavity/Mid Ear/Accessory Sinuses | 2 | 4.2 | - | 2 | 1.8 | - | 0 | 1.6 | - |
| MN of Larynx | 20 | 47.9 | 0.42 (0.26-0.64)** | 9 | 20.1 | 0.45 (0.21-0.85)** | 6 | 17.5 | 0.34 (0.12-0.74)** |
| MN of Bronchus, Trachea, Lung | 1088 | 1449.2 | 0.75 (0.71-0.80)** | 403 | 613.9 | 0.66 (0.59-0.72)** | 395 | 505.6 | 0.78 (0.71-0.86)** |
| MN of Bone | 2 | 7.7 | 0.26 (0.03-0.94)* | 0 | 3.5 | - | 3 | 3.2 | - |
| MN of Connective Tissue | 28 | 24.7 | 1.14 (0.75-1.64) | 13 | 11.0 | 1.18 (0.63-2.02) | 12 | 10.0 | 1.20 (0.62-2.10) |
| MN of Skin | 103 | 97.6 | 1.06 (0.86-1.28) | 45 | 44.3 | 1.02 (0.74-1.36) | 33 | 38.3 | 0.86 (0.59-1.21) |
| Malignant Melanoma | 88 | 73.0 | 1.21 (0.97-1.49) | 39 | 33.6 | 1.16 (0.83-1.59) | 27 | 29.4 | 0.92 (0.61-1.34) |
| Malignant Mesothelioma | 84 | 32.0 | 2.62 (2.11-3.23)** | 11 | 13.6 | 0.81 (0.42-1.41) | 10 | 10.3 | 0.97 (0.49-1.73) |
| MN of Breast | 4 | 5.2 | 0.76 (0.21-1.96) | 0 | 2.2 | - | 1 | 1.9 | - |
| MN of Cervix Uteri | 0 | 0 | - | 0 | 0 | - | 0 | 0 | - |
| MN of Body of Uterus (including Corpus Uteri) | 0 | 0 | - | 0 | 0 | - | 0 | 0 | - |
| MN of Ovary | 0 | 0 | - | 0 | 0 | - | 0 | 0 | - |
| MN of Prostate | 337 | 379.1 | 0.89 (0.80-0.99)* | 119 | 151.2 | 0.79 (0.65-0.94)** | 87 | 108.6 | 0.80 (0.64-0.99)* |
| MN of Testicular | 1 | 4.3 | - | 1 | 2.2 | - | 2 | 2.4 | - |
| MN of Bladder and Other Urinary | 97 | 119.0 | 0.82 (0.66-0.99)* | 26 | 50.5 | 0.52 (0.34-0.76)** | 35 | 37.6 | 0.93 (0.65-1.30) |
| MN of Bladder (Monson) | 96 | 116.9 | 0.82 (0.66-1.00) | 26 | 49.6 | 0.52 (0.34-0.77)** | 34 | 36.9 | 0.92 (0.64-1.29) |
| MN of Kidney | 95 | 111.7 | 0.85 (0.69-1.04) | 39 | 48.6 | 0.80 (0.57-1.10) | 33 | 41.6 | 0.79 (0.55-1.12) |
| MN of Central Nervous System (CNS) including Brain | 104 | 103.5 | 1.01 (0.82-1.22) | 65 | 47.0 | 1.38 (1.07-1.76)* | 32 | 42.8 | 0.75 (0.51-1.06) |
| MN of Brain | 103 | 101.6 | 1.01 (0.83-1.23) | 63 | 46.1 | 1.36 (1.05-1.75)* | 32 | 42.0 | 0.76 (0.52-1.08) |
| MN of Other/Ill-Defined Sites/Secondary Neoplasms | 266 | 297.0 | 0.90 (0.79-1.01) | 101 | 126.3 | 0.80 (0.65-0.97)* | 71 | 105.3 | 0.68 (0.53-0.85)** |
| MN of Lymphatic and Hematopoietic Tissue | 401 | 416.1 | 0.96 (0.87-1.06) | 159 | 180.1 | 0.88 (0.75-1.03) | 139 | 148.5 | 0.94 (0.79-1.11) |
| Hodgkin Lymphoma | 10 | 12.2 | 0.82 (0.39-1.51) | 4 | 5.7 | 0.71 (0.19-1.81) | 7 | 5.3 | 1.32 (0.53-2.71) |
| Non-Hodgkin Lymphoma | 148 | 157.0 | 0.94 (0.80-1.11) | 76 | 68.5 | 1.11 (0.87-1.39) | 47 | 56.4 | 0.83 (0.61-1.11) |
| Nodular/Follicular Lymphoma | 4 | 1.8 | - | 1 | 0.8 | - | 0 | 0.6 | - |
| Reticulosarcoma | 9 | 8.7 | 1.03 (0.47-1.96) | 5 | 3.8 | 1.32 (0.43-3.08) | 4 | 3.0 | - |
| T-Cell Lymphoid Variety | 1 | 1.1 | - | 0 | 0.5 | - | 0 | 0.4 | - |
| Lymphosarcoma | 2 | 4.4 | - | 3 | 1.8 | - | 1 | 1.4 | - |
| Other Lymphomas | 119 | 130.0 | 0.92 (0.76-1.10) | 62 | 56.6 | 1.10 (0.84-1.41) | 39 | 46.5 | 0.84 (0.60-1.15) |
| Multiple Myeloma | 62 | 76.2 | 0.81 (0.62-1.04) | 25 | 31.9 | 0.78 (0.51-1.16) | 23 | 26.3 | 0.87 (0.55-1.31) |
| Leukemia & Aleukemia | 172 | 161.0 | 1.07 (0.92-1.24) | 52 | 69.7 | 0.75 (0.56-0.98)* | 58 | 56.7 | 1.02 (0.78-1.32) |
| Acute Lymphocytic Leukemia (ALL) | 5 | 7.4 | 0.68 (0.22-1.58) | 3 | 3.4 | - | 1 | 3.3 | - |
| Chronic Lymphocytic Leukemia (CLL) | 33 | 35.1 | 0.94 (0.65-1.32) | 13 | 14.8 | 0.88 (0.47-1.50) | 11 | 11.2 | 0.98 (0.49-1.76) |
| Hairy Cell Leukemia | 0 | 1.3 | - | 0 | 0.6 | - | 1 | 0.4 | - |
| Acute Myelocytic Leukemia (AML) | 62 | 54.5 | 1.14 (0.87-1.46) | 23 | 24.0 | 0.96 (0.61-1.44) | 26 | 20.1 | 1.29 (0.84-1.89) |
| Chronic Myelocytic Leukemia (CML) | 14 | 14.9 | 0.94 (0.51-1.58) | 4 | 6.5 | 0.62 (0.17-1.58) | 5 | 5.6 | 0.90 (0.29-2.10) |
| Acute Monocytic Leukemia | 1 | 1.2 | - | 1 | 0.5 | - | 0 | 0.4 | - |
| Chronic Monocytic Leukemia | 0 | 0.1 | - | 0 | 0.1 | - | 0 | 0 | - |
| Acute Erythremia and Erythroleukemia | 0 | 0.6 | - | 0 | 0.2 | - | 0 | 0.2 | - |
| Megakaryocytic Leukemia | 0 | 0.2 | - | 0 | 0.1 | - | 0 | 0.1 | - |
| Acute Non-Lymphocytic Leukemia (ANLL) | 63 | 56.5 | 1.12 (0.86-1.43) | 24 | 24.8 | 0.97 (0.62-1.44) | 26 | 20.7 | 1.25 (0.82-1.84) |
| Other/Unspecified Leukemia (besides ANLL, CML, ALL, CLL) | 57 | 47.2 | 1.21 (0.92-1.57) | 8 | 20.2 | 0.40 (0.17-0.78)** | 15 | 15.9 | 0.94 (0.53-1.55) |
| Benign/In situ/Uncertain Behavior/Unspecified Neoplasms | 75 | 73.2 | 1.02 (0.81-1.28) | 28 | 31.6 | 0.88 (0.59-1.28) | 18 | 25.0 | 0.72 (0.43-1.14) |
| Benign CNS (including Brain) | 3 | 2.5 | - | 2 | 1.0 | - | 0 | 0.8 | - |
| Benign Brain | 1 | 0.5 | - | 0 | 0.2 | - | 0 | 0.2 | - |
| Uncertain Behavior/Unspecified - Brain/Spinal Cord | 15 | 17.8 | 0.84 (0.47-1.39) | 8 | 7.8 | 1.02 (0.44-2.01) | 5 | 6.8 | 0.74 (0.24-1.72) |
| All Diseases of Blood and Blood-Forming Organs | 43 | 51.4 | 0.84 (0.61-1.13) | 18 | 21.5 | 0.84 (0.50-1.32) | 16 | 18.4 | 0.87 (0.50-1.42) |
| Aplastic Anemia | 10 | 6.4 | 1.56 (0.75-2.86) | 1 | 2.7 | - | 1 | 2.1 | - |
| All Other Anemias | 9 | 13.8 | 0.65 (0.30-1.24) | 3 | 5.6 | 0.54 (0.11-1.57) | 2 | 4.8 | - |
| All Other Diseases of Blood-Forming Organs | 17 | 16.5 | 1.03 (0.60-1.65) | 9 | 6.9 | 1.31 (0.60-2.48) | 7 | 5.9 | 1.18 (0.48-2.44) |
| Other Specified Diseases of Blood/Blood-Form Org (including MDS) | 45 | 36.6 | 1.23 (0.90-1.64) | 16 | 15.7 | 1.02 (0.58-1.66) | 9 | 11.2 | 0.80 (0.37-1.53) |
| Endocrine/Nutritional/Metabolic Diseases | 342 | 525.6 | 0.65 (0.58-0.72)** | 102 | 225.6 | 0.45 (0.37-0.55)** | 111 | 196.4 | 0.56 (0.46-0.68)** |
| Diabetes Mellitus | 251 | 402.3 | 0.62 (0.55-0.71)** | 67 | 171.3 | 0.39 (0.30-0.50)** | 86 | 148.1 | 0.58 (0.46-0.72)** |
| Mental Disorders | 200 | 284.8 | 0.70 (0.61-0.81)** | 82 | 122.0 | 0.67 (0.54-0.84)** | 48 | 98.1 | 0.49 (0.36-0.65)** |
| Alcoholism | 20 | 68.3 | 0.29 (0.18-0.45)** | 10 | 31.4 | 0.32 (0.15-0.59)** | 5 | 33.6 | 0.15 (0.05-0.35)** |
| Drug Psychosis, Dependence, Poisoning | 48 | 102.0 | 0.47 (0.35-0.62)** | 20 | 53.2 | 0.38 (0.23-0.58)** | 32 | 64.6 | 0.50 (0.34-0.70)** |
| Nervous System/Sense Organ Disease | 412 | 452.1 | 0.91 (0.82-1.00) | 167 | 193.2 | 0.86 (0.74-1.01) | 132 | 142.7 | 0.92 (0.77-1.10) |
| Parkinson's Disease | 110 | 110.0 | 1.00 (0.82-1.21) | 45 | 46.3 | 0.97 (0.71-1.30) | 39 | 29.8 | 1.31 (0.93-1.79) |
| Motor Neuron Disease including Amyotrophic Lateral Sclerosis | 48 | 40.5 | 1.19 (0.88-1.57) | 23 | 18.0 | 1.28 (0.81-1.92) | 21 | 15.6 | 1.35 (0.83-2.06) |
| Multiple Sclerosis | 3 | 14.4 | 0.21 (0.04-0.61)** | 0 | 6.7 | - | 2 | 6.6 | 0.30 (0.04-1.10) |
| Circulatory Disease | 4366 | 5961.8 | 0.73 (0.71-0.75)** | 1586 | 2481.2 | 0.64 (0.61-0.67)** | 1440 | 1947.9 | 0.74 (0.70-0.78)** |
| All Heart Disease | 3564 | 4860.4 | 0.73 (0.71-0.76)** | 1308 | 2029.1 | 0.64 (0.61-0.68)** | 1148 | 1598.5 | 0.72 (0.68-0.76)** |
| Hypertension with Heart Disease | 127 | 167.4 | 0.76 (0.63-0.90)** | 40 | 68.9 | 0.58 (0.42-0.79)** | 45 | 64.7 | 0.70 (0.51-0.93)* |
| Ischemic Heart Disease | 2540 | 3500.7 | 0.73 (0.70-0.75)** | 936 | 1467.0 | 0.64 (0.60-0.68)** | 845 | 1141.5 | 0.74 (0.69-0.79)** |
| Acute Myocardial Infarction | 1120 | 1599.5 | 0.70 (0.66-0.74)** | 462 | 668.4 | 0.69 (0.63-0.76)** | 402 | 521.5 | 0.77 (0.70-0.85)** |
| Hypertension without Heart Disease | 57 | 86.5 | 0.66 (0.50-0.85)** | 11 | 35.4 | 0.31 (0.16-0.56)** | 20 | 29.7 | 0.67 (0.41-1.04) |
| Cerebrovascular Disease | 524 | 739.7 | 0.71 (0.65-0.77)** | 195 | 302.6 | 0.64 (0.56-0.74)** | 189 | 233.3 | 0.81 (0.70-0.93)** |
| Diseases of Arteries/Veins/Other Circulatory | 221 | 275.0 | 0.80 (0.70-0.92)** | 72 | 114.1 | 0.63 (0.49-0.80)** | 83 | 86.4 | 0.96 (0.76-1.19) |
| Aortic Aneurysm | 110 | 132.7 | 0.83 (0.68-1.00)* | 40 | 55.4 | 0.72 (0.52-0.98)* | 44 | 41.7 | 1.05 (0.77-1.42) |
| Non-Malignant Respiratory Disease | 846 | 1407.6 | 0.60 (0.56-0.64)** | 344 | 587.5 | 0.59 (0.52-0.65)** | 304 | 435.5 | 0.70 (0.62-0.78)** |
| Acute Respiratory Infections except Influenza/Pneumonia | 0 | 2.6 | - | 2 | 1.1 | - | 1 | 0.9 | - |
| Pneumonia | 199 | 338.3 | 0.59 (0.51-0.68)** | 77 | 137.8 | 0.56 (0.44-0.70)** | 57 | 102.3 | 0.56 (0.42-0.72)** |
| Influenza | 4 | 4.9 | - | 3 | 2.1 | - | 3 | 1.6 | - |
| Bronchitis, Emphysema, and Asthma | 96 | 156.8 | 0.61 (0.50-0.75)** | 38 | 65.1 | 0.58 (0.41-0.80)** | 35 | 49.3 | 0.71 (0.50-0.99)* |
| Bronchitis | 8 | 13.5 | 0.59 (0.26-1.17) | 2 | 5.4 | 0.37 (0.04-1.34) | 1 | 3.8 | - |
| Emphysema | 77 | 123.7 | 0.62 (0.49-0.78)** | 32 | 51.5 | 0.62 (0.42-0.88)** | 30 | 37.6 | 0.80 (0.54-1.14) |
| Asthma | 11 | 19.6 | 0.56 (0.28-1.01) | 4 | 8.2 | 0.49 (0.13-1.25) | 4 | 7.9 | 0.51 (0.14-1.30) |
| Pneumoconiosis and Other Respiratory Diseases | 547 | 904.9 | 0.60 (0.56-0.66)** | 224 | 381.4 | 0.59 (0.51-0.67)** | 208 | 281.3 | 0.74 (0.64-0.85)** |
| Chronic Obstructive Pulmonary Disease | 362 | 648.2 | 0.56 (0.50-0.62)** | 136 | 273.2 | 0.50 (0.42-0.59)** | 123 | 199.8 | 0.62 (0.51-0.73)** |
| Pneumoconiosis/Other Lung Diseases, External Agents | 62 | 96.1 | 0.64 (0.50-0.83)** | 24 | 39.7 | 0.61 (0.39-0.90)* | 26 | 27.8 | 0.94 (0.61-1.37) |
| Asbestosis | 18 | 6.1 | 2.97 (1.76-4.70)** | 4 | 2.6 | - | 2 | 1.8 | - |
| Silicosis and Anthracosilicosis | 0 | 7.2 | - | 1 | 2.9 | - | 3 | 1.9 | - |
| Digestive Disease | 369 | 619.3 | 0.60 (0.54-0.66)** | 117 | 273.2 | 0.43 (0.35-0.51)** | 130 | 246.7 | 0.53 (0.44-0.63)** |
| Ulcer of Stomach and Duodenum | 11 | 30.7 | 0.36 (0.18-0.64)** | 3 | 12.7 | 0.24 (0.05-0.69)** | 6 | 10.2 | 0.59 (0.22-1.28)* |
| Cirrhosis of Liver | 171 | 292.1 | 0.59 (0.50-0.68)** | 55 | 133.4 | 0.41 (0.31-0.54)** | 60 | 129.6 | 0.46 (0.35-0.60)** |
| Genitourinary Disease | 200 | 285.5 | 0.70 (0.61-0.81)** | 71 | 117.4 | 0.61 (0.47-0.76)** | 76 | 91.4 | 0.83 (0.66-1.04) |
| Nephritis and Nephrosis | 155 | 216.4 | 0.72 (0.61-0.84)** | 54 | 89.8 | 0.60 (0.45-0.79)** | 64 | 71.1 | 0.90 (0.69-1.15) |
| Skin/Subcutaneous Tissue Disease | 14 | 16.0 | 0.88 (0.48-1.47) | 3 | 6.6 | 0.46 (0.09-1.33) | 1 | 5.6 | 0.18 (0.01-1.00)* |
| Musculoskeletal Disease & Connective Tissue | 21 | 44.8 | 0.47 (0.29-0.72)** | 16 | 19.1 | 0.84 (0.48-1.36) | 8 | 16.0 | 0.50 (0.22-0.98)* |
| All External Causes of Death | 790 | 1160.2 | 0.68 (0.63-0.73)** | 354 | 560.0 | 0.63 (0.57-0.70)** | 457 | 595.3 | 0.77 (0.70-0.84)** |
| Accidents | 470 | 699.3 | 0.67 (0.61-0.74)** | 222 | 335.7 | 0.66 (0.58-0.75)** | 287 | 347.9 | 0.82 (0.73-0.93)** |
| Transportation Accidents | 216 | 316.2 | 0.68 (0.60-0.78)** | 107 | 156.3 | 0.68 (0.56-0.83)** | 169 | 169.1 | 1.00 (0.85-1.16) |
| Motor Vehicle Accidents (MVA) | 178 | 250.9 | 0.71 (0.61-0.82)** | 90 | 124.2 | 0.72 (0.58-0.89)** | 148 | 133.8 | 1.11 (0.94-1.30) |
| All Other Accidents besides MVA | 290 | 442.1 | 0.66 (0.58-0.74)** | 131 | 208.5 | 0.63 (0.52-0.75)** | 138 | 210.8 | 0.66 (0.55-0.77)** |
| Suicides | 239 | 292.4 | 0.82 (0.72-0.93)** | 104 | 144.7 | 0.72 (0.59-0.87)** | 136 | 151.0 | 0.90 (0.76-1.07) |
| Homicides and Legal Intervention | 59 | 131.3 | 0.45 (0.34-0.58)** | 21 | 61.4 | 0.34 (0.21-0.52)** | 24 | 76.3 | 0.31 (0.20-0.47)** |
| Congenital Anomalies | 12 | 23.3 | 0.52 (0.27-0.90)* | 10 | 10.9 | 0.92 (0.44-1.69) | 3 | 10.9 | 0.28 (0.06-0.80)* |

SMR (95% CI), standardized mortality ratio (95% confidence interval).

▪Expected deaths based on U.S. general population mortality rates.

*Statistically significant at *P* <0.05.

**Statistically significant at *P* <0.01.

MDS, Myelodysplastic Syndrome
