# Supplementary material for: Mortality of 196,826 Men and Women Working in U.S.-Based Petrochemical and Refinery Operations: Update 1979 to 2010
Source: J Occup Environ Med. 2021 Oct 20;64(3):250–62. doi: 10.1097/JOM.0000000000002416 (PMC8887844; doi:10.1097/JOM.0000000000002416)
Supplement: Supplemental Digital Content [file joem-64-0250-s007.docx]

Supplemental Digital Content 3, Table Listing Mortality Results of U.S.-based Petroleum Cohort by Manufacturing Job Titles (1979-2010) – MEN

| **Cause of Death** | **SKILLED CRAFTSMEN** | | | **OPERATORS** | | | **LABORERS** | | |
| --- | --- | --- | --- | --- | --- | --- | --- | --- | --- |
|  | **Observed** | **Expected*** | **SMR (95% CI)** | **Observed** | **Expected*** | **SMR (95% CI)** | **Observed** | **Expected*** | **SMR (95% CI)** |
| All Causes | 5873 | 7071.8 | 0.83 (0.81-0.85)** | 3864 | 4447.0 | 0.87 (0.84-0.90)** | 738 | 827.7 | 0.89 (0.83-0.96)** |
| Infectious and Parasitic Diseases | 101 | 215.8 | 0.47 (0.38-0.57)** | 94 | 185.5 | 0.51 (0.41-0.62)** | 35 | 56.3 | 0.62 (0.43-0.86)** |
| Tuberculosis | 1 | 5.0 | 0.20 (0.01-11.12) | 0 | 3.6 | - | 0 | 0.8 | - |
| Human Immunodeficiency Virus (HIV) Disease (incl. AIDS) | 20 | 90.1 | 0.22 (0.14-0.34)** | 25 | 98.4 | 0.25 (0.16-0.38)** | 19 | 37.8 | 0.50 (0.30-0.78)** |
| Malignant Neoplasms (MNs) | 1705 | 1910.2 | 0.89 (0.85-0.94)** | 1153 | 1163.2 | 0.99 (0.94-1.05) | 167 | 182.8 | 0.91 (0.78-1.06) |
| MN of Buccal Cavity and Pharynx | 27 | 41.3 | 0.65 (0.43-0.95)* | 22 | 27.4 | 0.80 (0.50-1.21) | 6 | 5.0 | 1.21 (0.44-2.63) |
| MN of Pharynx | 17 | 21.6 | 0.79 (0.46-1.26) | 8 | 14.7 | 0.54 (0.24-1.07) | 3 | 2.8 | - |
| MN of Digestive Organs and Peritoneum | 386 | 475.7 | 0.81 (0.73-0.90)** | 300 | 297.8 | 1.01 (0.90-1.13) | 52 | 49.6 | 1.05 (0.78-1.37) |
| MN of Esophagus | 46 | 65.5 | 0.70 (0.51-0.94)* | 34 | 42.8 | 0.80 (0.55-1.11) | 4 | 7.2 | 0.55 (0.15-1.41) |
| MN of Stomach | 48 | 51.0 | 0.94 (0.69-1.25) | 41 | 31.9 | 1.29 (0.92-1.75) | 7 | 5.3 | 1.32 (0.53-2.72) |
| MN of Large Intestine (Colon) | 123 | 151.0 | 0.81 (0.68-0.97)* | 89 | 89.5 | 0.99 (0.80-1.22) | 12 | 13.7 | 0.88 (0.45-1.54) |
| MN of Rectum | 19 | 28.4 | 0.67 (0.40-1.05) | 22 | 17.6 | 1.25 (0.78-1.89) | 0 | 3.0 | - |
| MN of Biliary Passages (including Gallbladder)/Liver | 43 | 65.4 | 0.66 (0.48-0.88)** | 45 | 44.6 | 1.01 (0.74-1.35) | 15 | 8.7 | 1.72 (0.96-2.84) |
| MN of Liver (Specified Primary or Unspecified) | 34 | 47.9 | 0.71 (0.49-0.99)* | 36 | 33.8 | 1.06 (0.75-1.47) | 11 | 7.0 | 1.57 (0.79-2.82) |
| MN of Pancreas | 93 | 100.2 | 0.93 (0.75-1.14) | 63 | 62.3 | 1.01 (0.78-1.29) | 13 | 10.1 | 1.29 (0.69-2.21) |
| MN of Respiratory System | 639 | 680.0 | 0.94 (0.87-1.02) | 407 | 409.1 | 1.00 (0.90-1.10) | 48 | 59.5 | 0.81 (0.59-1.07) |
| MN of Nasal Cavity/Mid Ear/Accessory Sinuses | 1 | 2.0 | - | 1 | 1.3 | - | 0 | 0.3 | - |
| MN of Larynx | 12 | 22.4 | 0.54 (0.28-0.94)* | 8 | 14.2 | 0.56 (0.24-1.11) | 2 | 2.3 | - |
| MN of Bronchus, Trachea, Lung | 623 | 652.6 | 0.96 (0.88-1.03) | 397 | 391.7 | 1.01 (0.92-1.12) | 46 | 56.6 | 0.81 (0.60-1.08) |
| MN of Bone | 1 | 3.6 | - | 1 | 2.6 | - | 0 | 0.6 | - |
| MN of Connective Tissue | 12 | 11.4 | 1.05 (0.54-1.83) | 8 | 8.0 | 1.00 (0.43-1.97) | 3 | 1.8 | - |
| MN of Skin | 33 | 44.7 | 0.74 (0.51-1.04) | 28 | 29.4 | 0.95 (0.63-1.38) | 6 | 5.6 | 1.08 (0.40-2.34) |
| Malignant Melanoma | 27 | 33.7 | 0.80 (0.53-1.17) | 26 | 22.5 | 1.16 (0.76-1.69) | 4 | 4.4 | - |
| Malignant Mesothelioma | 40 | 13.8 | 2.90 (2.10-3.91)** | 6 | 7.6 | 0.79 (0.32-1.64) | 0 | 0.9 | - |
| MN of Breast | 1 | 2.4 | - | 1 | 1.5 | - | 0 | 0.3 | - |
| MN of Cervix Uteri | 0 | 0 | - | 0 | 0 | - | 0 | 0 | - |
| MN of Body of Uterus (including Corpus Uteri) | 0 | 0 | - | 0 | 0 | - | 0 | 0 | - |
| MN of Ovary | 0 | 0 | - | 0 | 0 | - | 0 | 0 | - |
| MN of Prostate | 130 | 163.2 | 0.80 (0.66-0.95)** | 88 | 84.9 | 1.04 (0.83-1.28) | 14 | 9.3 | 1.51 (0.82-2.52) |
| MN of Testicular | 0 | 2.2 | - | 2 | 2.1 | - | 0 | 0.7 | - |
| MN of Bladder and Other Urinary | 44 | 50.8 | 0.87 (0.63-1.16) | 31 | 27.8 | 1.12 (0.76-1.58) | 1 | 3.5 | - |
| MN of Bladder (Monson) | 43 | 49.9 | 0.86 (0.62-1.16) | 31 | 27.2 | 1.14 (0.77-1.62) | 1 | 3.5 | - |
| MN of Kidney | 53 | 51.1 | 1.04 (0.78-1.36) | 31 | 32.1 | 0.96 (0.66-1.37) | 5 | 5.4 | 0.93 (0.30-2.18) |
| MN of Central Nervous System (CNS) including Brain | 42 | 48.6 | 0.86 (0.62-1.17) | 32 | 33.7 | 0.95 (0.65-1.34) | 6 | 7.0 | 0.85 (0.31-1.86) |
| MN of Brain | 42 | 47.7 | 0.88 (0.63-1.19) | 31 | 33.1 | 0.94 (0.64-1.33) | 6 | 6.9 | 0.87 (0.32-1.89) |
| MN of Other/Ill-Defined Sites/Secondary Neoplasms | 118 | 134.6 | 0.88 (0.73-1.05) | 71 | 82.8 | 0.86 (0.67-1.08) | 6 | 13.3 | 0.45 (0.16-0.98)* |
| MN of Lymphatic and Hematopoietic Tissue | 193 | 185.8 | 1.04 (0.90-1.20) | 118 | 114.7 | 1.03 (0.85-1.23) | 20 | 19.6 | 1.02 (0.62-1.58) |
| Hodgkin Lymphoma | 4 | 5.8 | 0.69 (0.19-1.76) | 7 | 4.4 | 1.60 (0.64-3.30) | 2 | 1.1 | - |
| Non-Hodgkin Lymphoma | 71 | 70.0 | 1.01 (0.79-1.28) | 41 | 43.2 | 0.95 (0.68-1.29) | 9 | 7.4 | 1.21 (0.55-2.30) |
| Nodular/Follicular Lymphoma | 1 | 0.8 | - | 0 | 0.5 | - | 0 | 0.1 | - |
| Reticulosarcoma | 4 | 3.9 | - | 1 | 2.3 | - | 0 | 0.4 | - |
| T-Cell Lymphoid Variety | 0 | 0.5 | - | 1 | 0.3 | - | 0 | 0.1 | - |
| Lymphosarcoma | 1 | 2.0 | - | 1 | 1.1 | - | 0 | 0.2 | - |
| Other Lymphomas | 59 | 57.9 | 1.02 (0.78-1.32) | 37 | 35.6 | 1.04 (0.73-1.43) | 9 | 6.1 | 1.48 (0.68-2.81) |
| Multiple Myeloma | 29 | 34.4 | 0.84 (0.56-1.21) | 17 | 20.8 | 0.82 (0.48-1.31) | 3 | 3.1 | - |
| Leukemia & Aleukemia | 85 | 71.2 | 1.19 (0.95-1.48) | 48 | 43.4 | 1.11 (0.82-1.46) | 4 | 7.4 | 0.54 (0.15-1.39) |
| Acute Lymphocytic Leukemia (ALL) | 1 | 3.5 | - | 1 | 2.7 | - | 0 | 0.8 | - |
| Chronic Lymphocytic Leukemia (CLL) | 16 | 15.1 | 1.06 (0.61-1.72) | 9 | 8.3 | 1.08 (0.49-2.05) | 1 | 1.0 | - |
| Hairy Cell Leukemia | 0 | 0.6 | - | 0 | 0.3 | - | 1 | 0 | - |
| Acute Myelocytic Leukemia (AML) | 31 | 24.3 | 1.27 (0.87-1.81) | 22 | 15.4 | 1.43 (0.90-2.17) | 1 | 2.7 | - |
| Chronic Myelocytic Leukemia (CML) | 6 | 6.9 | 0.87 (0.32-1.90) | 2 | 4.4 | - | 0 | 0.9 | - |
| Acute Monocytic Leukemia | 0 | 0.5 | - | 1 | 0.3 | - | 0 | 0 | - |
| Chronic Monocytic Leukemia | 0 | 0.1 | - | 0 | 0 | - | 0 | 0 | - |
| Acute Erythremia and Erythroleukemia | 0 | 0.3 | - | 0 | 0.1 | - | 0 | 0 | - |
| Megakaryocytic Leukemia | 0 | 0.1 | - | 0 | 0 | - | 0 | 0 | - |
| Acute Non-Lymphocytic Leukemia (ANLL) | 31 | 25.2 | 1.23 (0.84-1.74) | 23 | 15.8 | 1.45 (0.92-2.18) | 1 | 2.8 | - |
| Other/Unspecified Leukemia (besides ANLL, CML, ALL, CLL) | 31 | 20.6 | 1.51 (1.02-2.14)* | 13 | 12.1 | 1.07 (0.57-1.83) | 2 | 1.9 | - |
| Benign/In situ/Uncertain Behavior/Unspecified Neoplasms | 31 | 31.7 | 0.98 (0.66-1.39) | 14 | 19.0 | 0.74 (0.40-1.24) | 0 | 3.1 | - |
| Benign CNS (including Brain) | 0 | 1.1 | - | 1 | 0.7 | - | 0 | 0.1 | - |
| Benign Brain | 0 | 0.2 | - | 0 | 0.2 | - | 0 | 0 | - |
| Uncertain Behavior/Unspecified - Brain/Spinal Cord | 10 | 8.1 | 1.23 (0.59-2.26) | 2 | 5.3 | 0.37 (0.04-1.35) | 0 | 1.1 | - |
| All Diseases of Blood and Blood-Forming Organs | 23 | 23.2 | 0.99 (0.63-1.49) | 11 | 15.0 | 0.74 (0.37-1.32) | 6 | 3.2 | 1.89 (0.69-4.12) |
| Aplastic Anemia | 4 | 2.8 | - | 4 | 1.6 | - | 0 | 0.3 | - |
| All Other Anemias | 9 | 6.1 | 1.48 (0.68-2.80) | 2 | 4.0 | - | 0 | 0.9 | - |
| All Other Diseases of Blood-Forming Organs | 8 | 7.5 | 1.07 (0.46-2.11) | 3 | 4.8 | - | 2 | 1.0 | - |
| Other Specified Diseases of Blood/Blood-Form Org (including MDS) | 15 | 15.1 | 1.00 (0.56-1.64) | 11 | 8.1 | 1.36 (0.68-2.44) | 1 | 1.0 | - |
| Endocrine/Nutritional/Metabolic Diseases | 190 | 241.0 | 0.79 (0.68-0.91)** | 117 | 157.8 | 0.74 (0.61-0.89)** | 18 | 29.8 | 0.61 (0.36-0.96)* |
| Diabetes Mellitus | 148 | 184.1 | 0.80 (0.68-0.94)** | 88 | 118.8 | 0.74 (0.59-0.91)** | 16 | 21.4 | 0.75 (0.43-1.22) |
| Mental Disorders | 73 | 124.1 | 0.59 (0.46-0.74)** | 42 | 78.9 | 0.53 (0.38-0.72)** | 9 | 15.8 | 0.57 (0.26-1.08) |
| Alcoholism | 9 | 35.3 | 0.26 (0.12-0.48)** | 7 | 29.5 | 0.24 (0.10-0.49)** | 7 | 7.7 | 0.90 (0.36-1.86) |
| Drug Psychosis, Dependence, Poisoning | 31 | 51.9 | 0.60 (0.41-0.85)** | 40 | 58.5 | 0.68 (0.49-0.93)* | 19 | 23.0 | 0.83 (0.50-1.29) |
| Nervous System/Sense Organ Disease | 165 | 189.0 | 0.87 (0.75-1.02) | 91 | 107.0 | 0.85 (0.68-1.04) | 13 | 16.6 | 0.78 (0.42-1.34) |
| Parkinson's Disease | 36 | 43.5 | 0.83 (0.58-1.15) | 21 | 20.8 | 1.01 (0.62-1.54) | 1 | 1.8 | - |
| Motor Neuron Disease including Amyotrophic Lateral Sclerosis | 20 | 18.4 | 1.09 (0.66-1.68) | 15 | 11.9 | 1.26 (0.70-2.08) | 1 | 2.1 | - |
| Multiple Sclerosis | 2 | 7.1 | 0.28 (0.03-1.01) | 3 | 5.3 | 0.57 (0.12-1.66) | 0 | 1.2 | - |
| Circulatory Disease | 2303 | 2640.3 | 0.87 (0.84-0.91)** | 1335 | 1525.3 | 0.88 (0.83-0.92)** | 218 | 232.3 | 0.94 (0.82-1.07) |
| All Heart Disease | 1876 | 2156.0 | 0.87 (0.83-0.91)** | 1106 | 1249.1 | 0.88 (0.83-0.94)** | 182 | 190.7 | 0.96 (0.82-1.10) |
| Hypertension with Heart Disease | 70 | 80.1 | 0.87 (0.68-1.10) | 43 | 56.1 | 0.77 (0.56-1.03) | 12 | 11.9 | 1.01 (0.52-1.77) |
| Ischemic Heart Disease | 1342 | 1545.7 | 0.87 (0.82-0.92)** | 778 | 880.9 | 0.88 (0.82-0.95)** | 129 | 128.8 | 1.00 (0.84-1.19) |
| Acute Myocardial Infarction | 637 | 713.0 | 0.89 (0.82-0.97)** | 365 | 403.2 | 0.91 (0.82-1.00) | 55 | 57.3 | 0.96 (0.72-1.25) |
| Hypertension without Heart Disease | 25 | 39.0 | 0.64 (0.42-0.95)* | 21 | 24.5 | 0.86 (0.53-1.31) | 1 | 4.4 | - |
| Cerebrovascular Disease | 281 | 325.4 | 0.86 (0.77-0.97)* | 150 | 185.1 | 0.81 (0.69-0.95)** | 26 | 27.6 | 0.94 (0.61-1.38) |
| Diseases of Arteries/Veins/Other Circulatory | 121 | 119.9 | 1.01 (0.84-1.21) | 58 | 66.6 | 0.87 (0.66-1.13) | 9 | 9.6 | 0.94 (0.43-1.78) |
| Aortic Aneurysm | 68 | 57.5 | 1.18 (0.92-1.50) | 28 | 31.7 | 0.88 (0.59-1.28) | 2 | 4.5 | - |
| Non-Malignant Respiratory Disease | 455 | 601.7 | 0.76 (0.69-0.83)** | 253 | 328.4 | 0.77 (0.68-0.87)** | 43 | 43.6 | 0.99 (0.71-1.33) |
| Acute Respiratory Infections except Influenza/Pneumonia | 1 | 1.2 | - | 1 | 0.7 | - | 1 | 0.1 | - |
| Pneumonia | 94 | 146.6 | 0.64 (0.52-0.78)** | 44 | 80.2 | 0.55 (0.40-0.74)** | 8 | 11.8 | 0.68 (0.29-1.34) |
| Influenza | 4 | 2.1 | - | 1 | 1.3 | - | 0 | 0.2 | - |
| Bronchitis, Emphysema, and Asthma | 50 | 68.3 | 0.73 (0.54-0.96)* | 26 | 37.7 | 0.69 (0.45-1.01) | 3 | 5.3 | 0.57 (0.12-1.66) |
| Bronchitis | 5 | 5.9 | 0.85 (0.28-1.99) | 1 | 2.9 | - | 0 | 0.3 | - |
| Emphysema | 38 | 53.1 | 0.72 (0.51-0.98)* | 21 | 28 | 0.75 (0.46-1.15) | 3 | 3.2 | - |
| Asthma | 7 | 9.3 | 0.75 (0.30-1.55) | 4 | 6.8 | 0.59 (0.16-1.50) | 0 | 1.7 | - |
| Pneumoconiosis and Other Respiratory Diseases | 306 | 383.5 | 0.80 (0.71-0.89)** | 181 | 208.4 | 0.87 (0.75-1.01) | 31 | 26.1 | 1.19 (0.81-1.68) |
| Chronic Obstructive Pulmonary Disease | 204 | 273.8 | 0.74 (0.65-0.86)** | 123 | 146.6 | 0.84 (0.70-1.00) | 23 | 17.2 | 1.34 (0.85-2.00) |
| Pneumoconiosis/Other Lung Diseases, External Agents | 35 | 39.9 | 0.88 (0.61-1.22) | 20 | 20.8 | 0.96 (0.59-1.48) | 1 | 2.6 | - |
| Asbestosis | 13 | 2.5 | 5.27 (2.81-9.02)** | 3 | 1.3 | - | 0 | 0.1 | - |
| Silicosis and Anthracosilicosis | 2 | 3.1 | - | 3 | 1.4 | - | 0 | 0.1 | - |
| Digestive Disease | 177 | 293.2 | 0.60 (0.52-0.70)** | 131 | 202.3 | 0.65 (0.54-0.77)** | 32 | 42.1 | 0.76 (0.52-1.07) |
| Ulcer of Stomach and Duodenum | 9 | 13.9 | 0.65 (0.30-1.23) | 2 | 8.1 | 0.25 (0.03-0.89)* | 1 | 1.3 | - |
| Cirrhosis of Liver | 81 | 144.8 | 0.56 (0.44-0.70)** | 61 | 108.4 | 0.56 (0.43-0.72)** | 22 | 24.8 | 0.89 (0.56-1.34) |
| Genitourinary Disease | 86 | 124.6 | 0.69 (0.55-0.85)** | 70 | 72.6 | 0.96 (0.75-1.22) | 13 | 11.3 | 1.15 (0.61-1.96) |
| Nephritis and Nephrosis | 68 | 94.6 | 0.72 (0.56-0.91)** | 50 | 56.4 | 0.89 (0.66-1.17) | 10 | 9.1 | 1.09 (0.52-2.01) |
| Skin/Subcutaneous Tissue Disease | 8 | 7.3 | 1.09 (0.47-2.16) | 1 | 4.6 | - | 0 | 0.8 | - |
| Musculoskeletal Disease & Connective Tissue | 14 | 20.0 | 0.70 (0.38-1.18) | 4 | 12.7 | 0.32 (0.09-0.81)** | 1 | 2.4 | - |
| All External Causes of Death | 463 | 567.3 | 0.82 (0.74-0.89)** | 491 | 514.6 | 0.95 (0.87-1.04) | 175 | 172.4 | 1.02 (0.87-1.18) |
| Accidents | 305 | 338.5 | 0.90 (0.80-1.01) | 291 | 299.3 | 0.97 (0.86-1.09) | 97 | 97.0 | 1.00 (0.81-1.22) |
| Transportation Accidents | 153 | 156.6 | 0.98 (0.83-1.14) | 171 | 146.7 | 1.17 (1.00-1.35) | 57 | 50.7 | 1.12 (0.85-1.46) |
| Motor Vehicle Accidents (MVA) | 123 | 124.8 | 0.98 (0.82-1.18) | 150 | 117.2 | 1.28 (1.08-1.50)** | 51 | 40.9 | 1.25 (0.93-1.64) |
| All Other Accidents besides MVA | 181 | 210.7 | 0.86 (0.74-0.99)* | 140 | 179.4 | 0.78 (0.66-0.92)** | 46 | 55.3 | 0.83 (0.61-1.11) |
| Suicides | 120 | 142.7 | 0.84 (0.70-1.01) | 141 | 127.2 | 1.11 (0.93-1.31) | 51 | 41.1 | 1.24 (0.92-1.63) |
| Homicides and Legal Intervention | 27 | 67.6 | 0.40 (0.26-0.58)** | 46 | 70.2 | 0.66 (0.48-0.87)** | 22 | 28.1 | 0.78 (0.49-1.19) |
| Congenital Anomalies | 5 | 11.3 | 0.44 (0.14-1.03) | 5 | 9.1 | 0.55 (0.18-1.28) | 0 | 2.6 | - |

SMR (95% CI), standardized mortality ratio (95% confidence interval).

▪Expected deaths based on U.S. general population mortality rates.

*Statistically significant at *P* <0.05.

**Statistically significant at *P* <0.01.

MDS, Myelodysplastic Syndrome
