# Supplementary material for: Mortality of 196,826 Men and Women Working in U.S.-Based Petrochemical and Refinery Operations: Update 1979 to 2010
Source: J Occup Environ Med. 2021 Oct 20;64(3):250–62. doi: 10.1097/JOM.0000000000002416 (PMC8887844; doi:10.1097/JOM.0000000000002416)
Supplement: Supplemental Digital Content [file joem-64-0250-s008.docx]

Supplemental Digital Content 4, Table Listing Mortality Results of U.S.-based Petroleum Cohort by Other Operating Segments (1979-2010) – MEN

| **Cause of Death** | **COAL AND MINERALS** | | | **CORPORATE GLOBAL SERVICES** | | |
| --- | --- | --- | --- | --- | --- | --- |
|  | **Observed** | **Expected▪** | **SMR (95% CI)** | **Observed** | **Expected▪** | **SMR (95% CI)** |
| All Causes | 730 | 927.1 | 0.79 (0.73-0.85)** | 1517 | 2357.8 | 0.64 (0.61-0.68)** |
| Infectious and Parasitic Diseases | 20 | 34.9 | 0.57 (0.35-0.88)** | 63 | 70.8 | 0.89 (0.68-1.14) |
| Tuberculosis | 1 | 0.7 | - | 1 | 1.4 | - |
| Human Immunodeficiency Virus (HIV) Disease (incl. AIDS) | 9 | 17.2 | 0.52 (0.24-0.99)* | 45 | 29.2 | 1.54 (1.12-2.06)** |
| Malignant Neoplasms (MNs) | 223 | 250.2 | 0.89 (0.78-1.02) | 428 | 638.9 | 0.67 (0.61-0.74)** |
| MN of Buccal Cavity and Pharynx | 4 | 5.7 | 0.71 (0.19-1.81) | 10 | 13.2 | 0.76 (0.36-1.39) |
| MN of Pharynx | 4 | 3.0 | - | 6 | 6.9 | 0.88 (0.32-1.91) |
| MN of Digestive Organs and Peritoneum | 45 | 63.2 | 0.71 (0.52-0.95)* | 116 | 157.2 | 0.74 (0.61-0.88)** |
| MN of Esophagus | 4 | 9.2 | 0.44 (0.12-1.11) | 14 | 21.8 | 0.64 (0.35-1.08) |
| MN of Stomach | 6 | 6.6 | 0.91 (0.33-1.97) | 9 | 16.2 | 0.56 (0.25-1.06) |
| MN of Large Intestine (Colon) | 16 | 19.1 | 0.84 (0.48-1.36) | 44 | 49.9 | 0.88 (0.64-1.18) |
| MN of Rectum | 3 | 3.7 | - | 2 | 9.4 | 0.21 (0.03-0.77)** |
| MN of Biliary Passages (including Gallbladder)/Liver | 5 | 9.1 | 0.55 (0.18-1.28) | 12 | 21.4 | 0.56 (0.29-0.98)* |
| MN of Liver (Specified Primary or Unspecified) | 4 | 6.8 | 0.59 (0.16-1.50) | 6 | 15.5 | 0.39 (0.14-0.84)* |
| MN of Pancreas | 10 | 13.5 | 0.74 (0.36-1.36) | 31 | 33.7 | 0.92 (0.62-1.31) |
| MN of Respiratory System | 82 | 88.5 | 0.93 (0.74-1.15) | 124 | 227.6 | 0.54 (0.45-0.65)** |
| MN of Nasal Cavity/Mid Ear/Accessory Sinuses | 0 | 0.3 | - | 0 | 0.7 | - |
| MN of Larynx | 0 | 3.0 | - | 3 | 7.2 | 0.42 (0.09-1.22) |
| MN of Bronchus, Trachea, Lung | 81 | 84.9 | 0.95 (0.76-1.19) | 121 | 218.7 | 0.55 (0.46-0.66)** |
| MN of Bone | 0 | 0.5 | - | 0 | 1.2 | - |
| MN of Connective Tissue | 4 | 1.7 | - | 3 | 3.9 | - |
| MN of Skin | 10 | 6.6 | 1.51 (0.73-2.78) | 27 | 15.7 | 1.72 (1.13-2.50)* |
| Malignant Melanoma | 9 | 5.1 | 1.76 (0.80-3.34) | 23 | 11.9 | 1.93 (1.22-2.89)** |
| Malignant Mesothelioma | 0 | 1.6 | - | 2 | 4.8 | - |
| MN of Breast | 0 | 0.3 | - | 0 | 0.8 | - |
| MN of Cervix Uteri | 0 | 0 | - | 0 | 0 | - |
| MN of Body of Uterus (including Corpus Uteri) | 0 | 0 | - | 0 | 0 | - |
| MN of Ovary | 0 | 0 | - | 0 | 0 | - |
| MN of Prostate | 18 | 18.2 | 0.99 (0.59-1.56) | 39 | 52.7 | 0.74 (0.53-1.01) |
| MN of Testicular | 0 | 0.4 | - | 1 | 0.7 | - |
| MN of Bladder and Other Urinary | 2 | 6.1 | 0.33 (0.04-1.18) | 9 | 17.7 | 0.51 (0.23-0.97)* |
| MN of Bladder (Monson) | 2 | 6.0 | 0.33 (0.04-1.21) | 9 | 17.3 | 0.52 (0.24-0.98)* |
| MN of Kidney | 8 | 7.0 | 1.14 (0.49-2.24) | 10 | 17.4 | 0.57 (0.28-1.06) |
| MN of Central Nervous System (CNS) including Brain | 8 | 7.4 | 1.08 (0.47-2.13) | 9 | 16.7 | 0.54 (0.25-1.02) |
| MN of Brain | 8 | 7.3 | 1.10 (0.48-2.17) | 9 | 16.5 | 0.55 (0.25-1.04) |
| MN of Other/Ill-Defined Sites/Secondary Neoplasms | 15 | 17.7 | 0.85 (0.48-1.40) | 22 | 44.9 | 0.49 (0.31-0.74)** |
| MN of Lymphatic and Hematopoietic Tissue | 26 | 24.7 | 1.05 (0.69-1.54) | 55 | 63.6 | 0.86 (0.65-1.12) |
| Hodgkin Lymphoma | 1 | 0.9 | - | 2 | 2.0 | - |
| Non-Hodgkin Lymphoma | 7 | 9.4 | 0.74 (0.30-1.54) | 20 | 24.2 | 0.83 (0.51-1.28) |
| Nodular/Follicular Lymphoma | 0 | 0.1 | - | 0 | 0.3 | - |
| Reticulosarcoma | 0 | 0.5 | - | 2 | 1.3 | - |
| T-Cell Lymphoid Variety | 0 | 0.1 | - | 0 | 0.2 | - |
| Lymphosarcoma | 0 | 0.2 | - | 1 | 0.6 | - |
| Other Lymphomas | 7 | 7.7 | 0.91 (0.36-1.87) | 15 | 19.9 | 0.75 (0.42-1.24) |
| Multiple Myeloma | 8 | 4.4 | 1.81 (0.78-3.56) | 9 | 11.4 | 0.79 (0.36-1.50) |
| Leukemia & Aleukemia | 10 | 9.4 | 1.06 (0.51-1.96) | 24 | 24.6 | 0.98 (0.63-1.45) |
| Acute Lymphocytic Leukemia (ALL) | 0 | 0.6 | - | 0 | 1.2 | - |
| Chronic Lymphocytic Leukemia (CLL) | 1 | 1.8 | - | 7 | 5.2 | 1.35 (0.54-2.78) |
| Hairy Cell Leukemia | 0 | 0.1 | - | 0 | 0.2 | - |
| Acute Non-Lymphocytic Leukemia (ANLL) | 4 | 3.5 | - | 11 | 8.8 | 1.25 (0.62-2.23) |
| Acute Myelocytic Leukemia (AML) | 4 | 3.4 | - | 11 | 8.5 | 1.29 (0.64-2.30) |
| Chronic Myelocytic Leukemia (CML) | 1 | 0.9 | - | 3 | 2.3 | - |
| Acute Monocytic Leukemia | 0 | 0.1 | - | 0 | 0.2 | - |
| Chronic Monocytic Leukemia | 0 | 0 | - | 0 | 0 | - |
| Acute Erythremia and Erythroleukemia | 0 | 0 | - | 0 | 0.1 | - |
| Megakaryocytic Leukemia | 0 | 0 | - | 0 | 0 | - |
| Other/Unspecified Leukemia (besides ANLL, CML, ALL, CLL) | 4 | 2.6 | - | 3 | 7.1 | 0.42 (0.09-1.24) |
| Benign/In situ/Uncertain Behavior/Unspecified Neoplasms | 1 | 4.1 | - | 13 | 11.2 | 1.16 (0.62-1.99) |
| Benign CNS (including Brain) | 0 | 0.1 | - | 0 | 0.3 | - |
| Benign Brain | 0 | 0 | - | 0 | 0.1 | - |
| Uncertain Behavior/Unspecified - Brain/Spinal Cord | 1 | 1.1 | - | 7 | 2.8 | 2.53 (1.02-5.22)* |
| All Diseases of Blood and Blood-Forming Organs | 3 | 2.9 | - | 4 | 7.5 | 0.53 (0.14-1.36) |
| Aplastic Anemia | 1 | 0.3 | - | 0 | 0.9 | - |
| All Other Anemias | 2 | 0.7 | - | 0 | 2.0 | - |
| All Other Diseases of Blood-Forming Organs | 0 | 0.9 | - | 4 | 2.4 | - |
| Other Specified Diseases of Blood/Blood-Form Org (including MDS) | 0 | 1.8 | - | 5 | 5.5 | 0.91 (0.30-2.12) |
| Endocrine/Nutritional/Metabolic Diseases | 22 | 33.0 | 0.67 (0.42-1.01) | 44 | 80.9 | 0.54 (0.40-0.73)** |
| Diabetes Mellitus | 16 | 24.8 | 0.64 (0.37-1.05) | 33 | 61.5 | 0.54 (0.37-0.75)** |
| Mental Disorders | 4 | 16.0 | 0.25 (0.07-0.64)** | 27 | 42.8 | 0.63 (0.42-0.92)* |
| Alcoholism | 0 | 5.8 | - | 4 | 11.2 | 0.36 (0.10-0.91)* |
| Drug Psychosis, Dependence, Poisoning | 6 | 10.0 | 0.60 (0.22-1.31) | 13 | 18.2 | 0.71 (0.38-1.22) |
| Nervous System/Sense Organ Disease | 13 | 22.7 | 0.57 (0.30-0.98)* | 72 | 67.5 | 1.07 (0.83-1.34) |
| Parkinson's Disease | 0 | 4.5 | - | 20 | 15.9 | 1.26 (0.77-1.94) |
| Motor Neuron Disease including Amyotrophic Lateral Sclerosis | 1 | 2.6 | - | 8 | 6.5 | 1.24 (0.54-2.44) |
| Multiple Sclerosis | 0 | 1.2 | - | 5 | 2.4 | 2.05 (0.66-4.78) |
| Circulatory Disease | 252 | 320.4 | 0.79 (0.69-0.89)** | 494 | 864.9 | 0.57 (0.52-0.62)** |
| All Heart Disease | 205 | 263.4 | 0.78 (0.68-0.89)** | 410 | 707.4 | 0.58 (0.52-0.64)** |
| Hypertension with Heart Disease | 5 | 10.8 | 0.46 (0.15-1.08) | 19 | 24.9 | 0.76 (0.46-1.19) |
| Ischemic Heart Disease | 157 | 187.9 | 0.84 (0.71-0.98)* | 292 | 510.1 | 0.57 (0.51-0.64)** |
| Acute Myocardial Infarction | 80 | 86.4 | 0.92 (0.73-1.15) | 119 | 230.9 | 0.52 (0.43-0.62)** |
| Hypertension without Heart Disease | 3 | 4.9 | - | 6 | 12.7 | 0.47 (0.17-1.03) |
| Cerebrovascular Disease | 30 | 38.1 | 0.79 (0.53-1.12) | 56 | 105.5 | 0.53 (0.40-0.69)** |
| Diseases of Arteries/Veins/Other Circulatory | 14 | 14.0 | 1.00 (0.55-1.68) | 22 | 39.3 | 0.56 (0.35-0.85)** |
| Aortic Aneurysm | 11 | 6.7 | 1.65 (0.82-2.95) | 9 | 19.0 | 0.47 (0.22-0.90)* |
| Non-Malignant Respiratory Disease | 55 | 70.4 | 0.78 (0.59-1.02) | 110 | 204.6 | 0.54 (0.44-0.65)** |
| Acute Respiratory Infections except Influenza/Pneumonia | 0 | 0.1 | - | 0 | 0.4 | - |
| Pneumonia | 10 | 16.6 | 0.60 (0.29-1.11) | 29 | 47.5 | 0.61 (0.41-0.88)** |
| Influenza | 0 | 0.3 | - | 2 | 0.7 | - |
| Bronchitis, Emphysema, and Asthma | 7 | 7.9 | 0.88 (0.36-1.82) | 15 | 22.4 | 0.67 (0.38-1.11) |
| Bronchitis | 0 | 0.6 | - | 2 | 1.8 | - |
| Emphysema | 6 | 6.1 | 0.99 (0.36-2.16) | 9 | 17.7 | 0.51 (0.23-0.97)* |
| Asthma | 1 | 1.3 | - | 4 | 2.9 | - |
| Pneumoconiosis and Other Respiratory Diseases | 38 | 45.4 | 0.84 (0.59-1.15) | 64 | 133.6 | 0.48 (0.37-0.61)** |
| Chronic Obstructive Pulmonary Disease | 24 | 32.2 | 0.74 (0.48-1.11) | 41 | 95.6 | 0.43 (0.31-0.58)** |
| Pneumoconiosis/Other Lung Diseases, External Agents | 5 | 4.4 | 1.13 (0.37-2.64) | 7 | 13.8 | 0.51 (0.20-1.05) |
| Asbestosis | 0 | 0.3 | - | 1 | 0.9 | - |
| Silicosis and Anthracosilicosis | 1 | 0.3 | - | 0 | 1.0 | - |
| Digestive Disease | 18 | 42.1 | 0.43 (0.25-0.68)** | 66 | 96.4 | 0.68 (0.53-0.87)** |
| Ulcer of Stomach and Duodenum | 0 | 1.7 | - | 5 | 4.4 | 1.14 (0.37-2.66) |
| Cirrhosis of Liver | 7 | 22.6 | 0.31 (0.12-0.64)** | 35 | 47.2 | 0.74 (0.52-1.03) |
| Genitourinary Disease | 9 | 14.9 | 0.60 (0.28-1.14) | 29 | 41.5 | 0.70 (0.47-1.00) |
| Nephritis and Nephrosis | 8 | 11.6 | 0.69 (0.30-1.36) | 26 | 31.9 | 0.81 (0.53-1.19) |
| Skin/Subcutaneous Tissue Disease | 0 | 1.0 | - | 2 | 2.4 | - |
| Musculoskeletal Disease & Connective Tissue | 2 | 2.6 | - | 4 | 6.8 | 0.59 (0.16-1.51) |
| All External Causes of Death | 101 | 99.8 | 1.01 (0.82-1.23) | 127 | 194.1 | 0.65 (0.54-0.78)** |
| Accidents | 65 | 58.6 | 1.11 (0.86-1.41) | 60 | 116.1 | 0.52 (0.39-0.67)** |
| Transportation Accidents | 34 | 29.2 | 1.17 (0.81-1.63) | 19 | 53.9 | 0.35 (0.21-0.55)** |
| Motor Vehicle Accidents (MVA) | 31 | 23.5 | 1.32 (0.90-1.87) | 15 | 42.4 | 0.35 (0.20-0.58)** |
| All Other Accidents besides MVA | 34 | 34.6 | 0.98 (0.68-1.37) | 45 | 72.6 | 0.62 (0.45-0.83)** |
| Suicides | 24 | 25.9 | 0.93 (0.59-1.38) | 47 | 50.0 | 0.94 (0.69-1.25) |
| Homicides and Legal Intervention | 12 | 12.0 | 1.00 (0.52-1.75) | 13 | 21.7 | 0.60 (0.32-1.03) |
| Congenital Anomalies | 1 | 1.9 | - | 1 | 3.8 | - |

SMR (95% CI), standardized mortality ratio (95% confidence interval).

▪Expected deaths based on U.S. general population mortality rates.

*Statistically significant at *P* <0.05.

**Statistically significant at *P* <0.01.

MDS, Myelodysplastic Syndrome
