# Supplementary material for: Mortality of 196,826 Men and Women Working in U.S.-Based Petrochemical and Refinery Operations: Update 1979 to 2010
Source: J Occup Environ Med. 2021 Oct 20;64(3):250–62. doi: 10.1097/JOM.0000000000002416 (PMC8887844; doi:10.1097/JOM.0000000000002416)
Supplement: Supplemental Digital Content [file joem-64-0250-s009.docx]

Supplemental Digital Content 5, Table Listing Mortality Results of U.S.-based Petroleum Cohort by Non-manufacturing Job Titles (1979-2010) – MEN

| **Cause of Death** | **PROFESSIONALS** | | | **MANAGERS / SUPERVISORS** | | | **OFFICE / CLERICALS** | | |
| --- | --- | --- | --- | --- | --- | --- | --- | --- | --- |
|  | **Observed** | **Expected▪** | **SMR (95% CI)** | **Observed** | **Expected▪** | **SMR (95% CI)** | **Observed** | **Expected▪** | **SMR (95% CI)** |
| All Causes | 6964 | 12143.8 | 0.57 (0.56-0.59)** | 5344 | 8472.0 | 0.63 (0.61-0.65)** | 1153 | 1311.3 | 0.88 (0.83-0.93)** |
| Infectious and Parasitic Diseases | 187 | 376.8 | 0.50 (0.43-0.57)** | 78 | 180.5 | 0.43 (0.34-0.54)** | 99 | 44.8 | 2.21 (1.80-2.69)** |
| Tuberculosis | 0 | 6.8 | - | 2 | 4.2 | - | 2 | 0.9 | - |
| Human Immunodeficiency Virus (HIV) Disease (incl. AIDS) | 74 | 163.6 | 0.45 (0.36-0.57)** | 10 | 43.8 | 0.23 (0.11-0.42)** | 80 | 21.7 | 3.70 (2.93-4.60)** |
| Malignant Neoplasms (MNs) | 2156 | 3218.5 | 0.67 (0.64-0.70)** | 1758 | 2392.6 | 0.74 (0.70-0.77)** | 298 | 335.1 | 0.89 (0.79-1.00)* |
| MN of Buccal Cavity and Pharynx | 39 | 66.4 | 0.59 (0.42-0.80)** | 22 | 46.2 | 0.48 (0.30-0.72)** | 3 | 7.0 | 0.43 (0.09-1.24) |
| MN of Pharynx | 19 | 34.2 | 0.56 (0.34-0.87)** | 8 | 23.4 | 0.34 (0.15-0.68)** | 2 | 3.7 | - |
| MN of Digestive Organs and Peritoneum | 556 | 792.0 | 0.70 (0.64-0.76)** | 422 | 574.1 | 0.74 (0.67-0.81)** | 81 | 82.9 | 0.98 (0.78-1.22) |
| MN of Esophagus | 66 | 108.1 | 0.61 (0.47-0.78)** | 55 | 77.7 | 0.71 (0.53-0.92)** | 11 | 11.0 | 1.00 (0.50-1.79) |
| MN of Stomach | 50 | 80.9 | 0.62 (0.46-0.81)** | 31 | 58.6 | 0.53 (0.36-0.75)** | 10 | 9.0 | 1.11 (0.53-2.04) |
| MN of Large Intestine (Colon) | 178 | 253.5 | 0.70 (0.60-0.81)** | 144 | 189.3 | 0.76 (0.64-0.90)** | 26 | 26.9 | 0.97 (0.63-1.42) |
| MN of Rectum | 39 | 47.8 | 0.82 (0.58-1.12) | 19 | 34.5 | 0.55 (0.33-0.86)** | 10 | 5.0 | 2.01 (0.96-3.69) |
| MN of Biliary Passages (including Gallbladder)/Liver | 64 | 108.2 | 0.59 (0.46-0.76)** | 50 | 72.5 | 0.69 (0.51-0.91)** | 8 | 11.3 | 0.71 (0.31-1.39) |
| MN of Liver (Specified Primary or Unspecified) | 38 | 78.1 | 0.49 (0.34-0.67)** | 31 | 50.4 | 0.62 (0.42-0.87)** | 6 | 8.3 | 0.73 (0.27-1.58) |
| MN of Pancreas | 141 | 169.3 | 0.83 (0.70-0.98)* | 110 | 124.4 | 0.88 (0.73-1.07) | 14 | 17.2 | 0.81 (0.44-1.36) |
| MN of Respiratory System | 571 | 1134.7 | 0.50 (0.46-0.55)** | 552 | 870.2 | 0.63 (0.58-0.69)** | 96 | 117.4 | 0.82 (0.66-1.00)* |
| MN of Nasal Cavity/Mid Ear/Accessory Sinuses | 1 | 3.3 | - | 0 | 2.3 | - | 0 | 0.3 | - |
| MN of Larynx | 12 | 35.8 | 0.34 (0.17-0.59)** | 7 | 26.2 | 0.27 (0.11-0.55)** | 2 | 3.8 | - |
| MN of Bronchus, Trachea, Lung | 558 | 1090.6 | 0.51 (0.47-0.56)** | 541 | 838.2 | 0.64 (0.59-0.70)** | 92 | 112.7 | 0.82 (0.66-1.00) |
| MN of Bone | 2 | 6.4 | 0.31 (0.04-1.12) | 3 | 3.9 | - | 0 | 0.7 | - |
| MN of Connective Tissue | 25 | 20.3 | 1.23 (0.80-1.82) | 15 | 13.1 | 1.14 (0.64-1.89) | 2 | 2.1 | - |
| MN of Skin | 93 | 81.4 | 1.14 (0.92-1.40) | 58 | 55.7 | 1.04 (0.79-1.35) | 14 | 7.7 | 1.83 (1.00-3.07)* |
| Malignant Melanoma | 85 | 62.0 | 1.37 (1.10-1.70)** | 43 | 41.7 | 1.03 (0.75-1.39) | 11 | 5.7 | 1.93 (0.96-3.46) |
| Malignant Mesothelioma | 27 | 24.2 | 1.12 (0.75-1.60) | 36 | 19.2 | 1.88 (1.33-2.57)** | 2 | 2.4 | - |
| MN of Breast | 0 | 4.0 | - | 2 | 2.9 | - | 0 | 0.4 | - |
| MN of Cervix Uteri | 0 | 0 | - | 0 | 0 | - | 0 | 0 | - |
| MN of Body of Uterus (including Corpus Uteri) | 0 | 0 | - | 0 | 0 | - | 0 | 0 | - |
| MN of Ovary | 0 | 0 | - | 0 | 0 | - | 0 | 0 | - |
| MN of Prostate | 219 | 266.8 | 0.82 (0.72-0.94)** | 176 | 211.1 | 0.83 (0.72-0.97)* | 32 | 30.8 | 1.04 (0.71-1.47) |
| MN of Testicular | 1 | 4.4 | - | 0 | 1.5 | - | 0 | 0.4 | - |
| MN of Bladder and Other Urinary | 62 | 90.2 | 0.69 (0.53-0.88)** | 37 | 70.3 | 0.53 (0.37-0.72)** | 5 | 9.3 | 0.54 (0.17-1.25) |
| MN of Bladder (Monson) | 60 | 88.6 | 0.68 (0.52-0.87)** | 37 | 69.1 | 0.54 (0.38-0.74)** | 5 | 9.1 | 0.55 (0.18-1.28) |
| MN of Kidney | 55 | 87.9 | 0.63 (0.47-0.82)** | 44 | 64.0 | 0.69 (0.50-0.92)* | 5 | 8.7 | 0.57 (0.19-1.34) |
| MN of Central Nervous System (CNS) including Brain | 80 | 86.7 | 0.92 (0.73-1.15) | 69 | 56.8 | 1.22 (0.94-1.54) | 7 | 8.3 | 0.84 (0.34-1.74) |
| MN of Brain | 79 | 85.1 | 0.93 (0.74-1.16) | 67 | 55.8 | 1.20 (0.93-1.52) | 7 | 8.1 | 0.86 (0.35-1.78) |
| MN of Other/Ill-Defined Sites/Secondary Neoplasms | 171 | 226.2 | 0.76 (0.65-0.88)** | 116 | 166.6 | 0.70 (0.58-0.84)** | 12 | 23.6 | 0.51 (0.26-0.89)* |
| MN of Lymphatic and Hematopoietic Tissue | 257 | 324.2 | 0.79 (0.70-0.90)** | 213 | 236.2 | 0.90 (0.78-1.03) | 40 | 33.3 | 1.20 (0.86-1.64) |
| Hodgkin Lymphoma | 12 | 10.5 | 1.14 (0.59-2.00) | 4 | 6.0 | 0.67 (0.18-1.71) | 1 | 1.1 | - |
| Non-Hodgkin Lymphoma | 96 | 123.7 | 0.78 (0.63-0.95)* | 72 | 89.8 | 0.80 (0.63-1.01) | 22 | 12.5 | 1.76 (1.10-2.66)* |
| Nodular/Follicular Lymphoma | 1 | 1.4 | - | 1 | 1.0 | - | 1 | 0.1 | - |
| Reticulosarcoma | 10 | 6.8 | 1.48 (0.71-2.71) | 4 | 5.0 | 0.80 (0.22-2.06) | 0 | 0.7 | - |
| T-Cell Lymphoid Variety | 0 | 0.8 | - | 0 | 0.6 | - | 0 | 0.1 | - |
| Lymphosarcoma | 5 | 3.2 | 1.55 (0.50-3.62) | 2 | 2.4 | - | 0 | 0.4 | - |
| Other Lymphomas | 73 | 102.1 | 0.72 (0.56-0.90)** | 58 | 74.3 | 0.78 (0.59-1.01) | 21 | 10.4 | 2.03 (1.26-3.10)** |
| Multiple Myeloma | 46 | 56.8 | 0.81 (0.59-1.08) | 43 | 43.0 | 1.00 (0.72-1.35) | 8 | 6.0 | 1.33 (0.57-2.61) |
| Leukemia & Aleukemia | 99 | 125.6 | 0.79 (0.64-0.96)* | 92 | 91.9 | 1.00 (0.81-1.23) | 8 | 12.9 | 0.62 (0.27-1.22) |
| Acute Lymphocytic Leukemia (ALL) | 5 | 6.4 | 0.78 (0.25-1.81) | 5 | 3.6 | 1.40 (0.46-3.27) | 0 | 0.7 | - |
| Chronic Lymphocytic Leukemia (CLL) | 16 | 26.3 | 0.61 (0.35-0.99)* | 20 | 20.7 | 0.97 (0.59-1.49) | 4 | 2.7 | - |
| Hairy Cell Leukemia | 0 | 1.0 | - | 0 | 0.8 | - | 0 | 0.1 | - |
| Acute Myelocytic Leukemia (AML) | 47 | 43.4 | 1.08 (0.80-1.44) | 32 | 31.5 | 1.02 (0.70-1.44) | 2 | 4.3 | - |
| Chronic Myelocytic Leukemia (CML) | 10 | 11.7 | 0.86 (0.41-1.57) | 13 | 8.0 | 1.63 (0.87-2.78) | 0 | 1.3 | - |
| Acute Monocytic Leukemia | 1 | 0.9 | - | 1 | 0.7 | - | 0 | 0.1 | - |
| Chronic Monocytic Leukemia | 0 | 0.1 | - | 0 | 0.1 | - | 0 | 0 | - |
| Acute Erythremia and Erythroleukemia | 0 | 0.4 | - | 0 | 0.3 | - | 0 | 0 | - |
| Megakaryocytic Leukemia | 0 | 0.1 | - | 0 | 0.1 | - | 0 | 0 | - |
| Acute Non-Lymphocytic Leukemia (ANLL) | 48 | 44.9 | 1.07 (0.79-1.42) | 33 | 32.6 | 1.01 (0.70-1.42) | 2 | 4.5 | - |
| Other/Unspecified Leukemia (besides ANLL, CML, ALL, CLL) | 20 | 36.3 | 0.55 (0.34-0.85)** | 21 | 27.1 | 0.78 (0.48-1.18) | 2 | 3.8 | - |
| Benign/In situ/Uncertain Behavior/Unspecified Neoplasms | 53 | 56.9 | 0.93 (0.70-1.22) | 41 | 42.1 | 0.97 (0.70-1.32) | 10 | 5.9 | 1.71 (0.82-3.14) |
| Benign CNS (including Brain) | 2 | 1.8 | - | 1 | 1.3 | - | 0 | 0.2 | - |
| Benign Brain | 0 | 0.4 | - | 0 | 0.3 | - | 0 | 0 | - |
| Uncertain Behavior/Unspecified - Brain/Spinal Cord | 19 | 14.3 | 1.33 (0.80-2.07) | 11 | 9.7 | 1.14 (0.57-2.04) | 1 | 1.5 | - |
| All Diseases of Blood and Blood-Forming Organs | 32 | 38.9 | 0.82 (0.56-1.16) | 19 | 26.2 | 0.73 (0.44-1.14) | 2 | 4.6 | - |
| Aplastic Anemia | 3 | 4.9 | - | 3 | 3.5 | - | 0 | 0.5 | - |
| All Other Anemias | 5 | 10.1 | 0.50 (0.16-1.16) | 3 | 6.7 | 0.45 (0.09-1.32) | 0 | 1.3 | - |
| All Other Diseases of Blood-Forming Organs | 16 | 12.5 | 1.28 (0.73-2.08) | 8 | 8.3 | 0.96 (0.42-1.90) | 2 | 1.5 | - |
| Other Specified Diseases of Blood/Blood-Form Org (including MDS) | 29 | 27.8 | 1.04 (0.70-1.50) | 25 | 22.2 | 1.12 (0.73-1.66) | 4 | 2.8 | - |
| Endocrine/Nutritional/Metabolic Diseases | 200 | 409.0 | 0.49 (0.42-0.56)** | 118 | 282.8 | 0.42 (0.34-0.50)** | 34 | 43.2 | 0.79 (0.55-1.10) |
| Diabetes Mellitus | 142 | 309.1 | 0.46 (0.39-0.54)** | 79 | 218.6 | 0.36 (0.29-0.45)** | 21 | 32.7 | 0.64 (0.40-0.98)* |
| Mental Disorders | 137 | 223.0 | 0.61 (0.52-0.73)** | 116 | 148.7 | 0.78 (0.64-0.94)** | 22 | 24.5 | 0.90 (0.56-1.36) |
| Alcoholism | 17 | 58.8 | 0.29 (0.17-0.46)** | 3 | 29.5 | 10 (0.02-0.30)** | 2 | 6.2 | 0.32 (0.04-1.17) |
| Drug Psychosis, Dependence, Poisoning | 11 | 106.9 | 0.10 (0.05-0.18)** | 3 | 24.1 | 0.12 (0.03-0.36)** | 8 | 11.9 | 0.67 (0.29-1.33) |
| Nervous System/Sense Organ Disease | 308 | 348.1 | 0.88 (0.79-0.99)* | 243 | 259.0 | 0.94 (0.82-1.06) | 41 | 36.7 | 1.12 (0.80-1.51) |
| Parkinson's Disease | 97 | 81.8 | 1.19 (0.96-1.45) | 74 | 66.9 | 1.11 (0.87-1.39) | 5 | 8.7 | 0.58 (0.19-1.34) |
| Motor Neuron Disease including Amyotrophic Lateral Sclerosis | 37 | 32.8 | 1.13 (0.80-1.56) | 33 | 23.4 | 1.41 (0.97-1.98) | 5 | 3.1 | 1.60 (0.52-3.72) |
| Multiple Sclerosis | 6 | 12.6 | 0.48 (0.18-1.04) | 2 | 7.7 | 0.26 (0.03-0.94)* | 2 | 1.2 | - |
| Circulatory Disease | 2435 | 4447.5 | 0.55 (0.53-0.57)** | 1941 | 3285.9 | 0.59 (0.56-0.62)** | 421 | 486.0 | 0.87 (0.78-0.95)** |
| All Heart Disease | 1978 | 3638.8 | 0.54 (0.52-0.57)** | 1586 | 2687.6 | 0.59 (0.56-0.62)** | 337 | 394.8 | 0.85 (0.77-0.95)** |
| Hypertension with Heart Disease | 64 | 124.8 | 0.51 (0.40-0.66)** | 39 | 79.4 | 0.49 (0.35-0.67)** | 16 | 14.7 | 1.09 (0.62-1.77) |
| Ischemic Heart Disease | 1402 | 2629.6 | 0.53 (0.51-0.56)** | 1158 | 1963.6 | 0.59 (0.56-0.62)** | 236 | 281.4 | 0.84 (0.74-0.95)** |
| Acute Myocardial Infarction | 628 | 1193.3 | 0.53 (0.49-0.57)** | 553 | 897.4 | 0.62 (0.57-0.67)** | 98 | 128.2 | 0.76 (0.62-0.93)** |
| Hypertension without Heart Disease | 30 | 63.6 | 0.47 (0.32-0.67)** | 16 | 44.9 | 0.36 (0.20-0.58)** | 11 | 7.3 | 1.51 (0.75-2.69) |
| Cerebrovascular Disease | 318 | 540.9 | 0.59 (0.52-0.66)** | 233 | 400.2 | 0.58 (0.51-0.66)** | 44 | 61.4 | 0.72 (0.52-0.96)* |
| Diseases of Arteries/Veins/Other Circulatory | 109 | 204.1 | 0.53 (0.44-0.64)** | 106 | 153.1 | 0.69 (0.57-0.84)** | 29 | 22.4 | 1.29 (0.87-1.86) |
| Aortic Aneurysm | 55 | 98.8 | 0.56 (0.42-0.72)** | 49 | 75.1 | 0.65 (0.48-0.86)** | 15 | 10.7 | 1.40 (0.78-2.31) |
| Non-Malignant Respiratory Disease | 511 | 1049.2 | 0.49 (0.45-0.53)** | 411 | 807.9 | 0.51 (0.46-0.56)** | 86 | 113.0 | 0.76 (0.61-0.94)** |
| Acute Respiratory Infections except Influenza/Pneumonia | 2 | 2.0 | - | 0 | 1.3 | - | 0 | 0.2 | - |
| Pneumonia | 126 | 248.3 | 0.51 (0.42-0.60)** | 87 | 182.0 | 0.48 (0.38-0.59)** | 27 | 28.5 | 0.95 (0.62-1.38) |
| Influenza | 3 | 3.9 | - | 1 | 2.6 | - | 2 | 0.4 | - |
| Bronchitis, Emphysema, and Asthma | 66 | 115.5 | 0.57 (0.44-0.73)** | 46 | 88.6 | 0.52 (0.38-0.69)** | 10 | 12.7 | 0.79 (0.38-1.45) |
| Bronchitis | 5 | 9.6 | 0.52 (0.17-1.22) | 2 | 7.4 | 0.27 (0.03-0.98)* | 0 | 1.1 | - |
| Emphysema | 57 | 91.0 | 0.63 (0.48-0.81)** | 39 | 72.3 | 0.54 (0.38-0.74)** | 8 | 9.7 | 0.82 (0.35-1.62) |
| Asthma | 4 | 14.9 | 0.27 (0.07-0.69)** | 5 | 9.0 | 0.56 (0.18-1.30) | 2 | 1.8 | - |
| Pneumoconiosis and Other Respiratory Diseases | 314 | 679.5 | 0.46 (0.41-0.52)** | 277 | 533.3 | 0.52 (0.46-0.58)** | 47 | 71.2 | 0.66 (0.48-0.88)** |
| Chronic Obstructive Pulmonary Disease | 187 | 485.7 | 0.38 (0.33-0.44)** | 166 | 386.4 | 0.43 (0.37-0.50)** | 32 | 50.5 | 0.63 (0.43-0.89)** |
| Pneumoconiosis/Other Lung Diseases, External Agents | 32 | 71.3 | 0.45 (0.31-0.63)** | 32 | 54.6 | 0.59 (0.40-0.83)** | 4 | 7.8 | 0.51 (0.14-1.31) |
| Asbestosis | 2 | 4.5 | - | 7 | 3.7 | 1.88 (0.75-3.86) | 0 | 0.5 | - |
| Silicosis and Anthracosilicosis | 0 | 5.2 | - | 2 | 4.0 | - | 0 | 0.6 | - |
| Digestive Disease | 220 | 499.2 | 0.44 (0.38-0.50)** | 137 | 319.9 | 0.43 (0.36-0.51)** | 27 | 51.8 | 0.52 (0.34-0.76)** |
| Ulcer of Stomach and Duodenum | 6 | 22.8 | 0.26 (0.10-0.57)** | 7 | 16.4 | 0.43 (0.17-0.88)* | 1 | 2.6 | - |
| Cirrhosis of Liver | 94 | 245.9 | 0.38 (0.31-0.47)** | 63 | 145.2 | 0.43 (0.33-0.56)** | 18 | 24.6 | 0.73 (0.43-1.16) |
| Genitourinary Disease | 117 | 210.2 | 0.56 (0.46-0.67)** | 102 | 155.2 | 0.66 (0.54-0.80)** | 20 | 23.7 | 0.84 (0.52-1.30) |
| Nephritis and Nephrosis | 94 | 160.9 | 0.58 (0.47-0.72)** | 87 | 118.2 | 0.74 (0.59-0.91)** | 17 | 17.9 | 0.95 (0.55-1.52) |
| Skin/Subcutaneous Tissue Disease | 6 | 11.9 | 0.51 (0.19-1.10) | 5 | 8.2 | 0.61 (0.20-1.42) | 1 | 1.4 | - |
| Musculoskeletal Disease & Connective Tissue | 15 | 34.7 | 0.43 (0.24-0.71)** | 15 | 24.3 | 0.62 (0.35-1.02) | 1 | 3.7 | - |
| All External Causes of Death | 439 | 1075.0 | 0.41 (0.37-0.45)** | 257 | 454.3 | 0.57 (0.50-0.64)** | 75 | 120.7 | 0.62 (0.49-0.78)** |
| Accidents | 254 | 642.6 | 0.40 (0.35-0.45)** | 176 | 283.4 | 0.62 (0.53-0.72)** | 29 | 70.6 | 0.41 (0.28-0.59)** |
| Transportation Accidents | 112 | 302.2 | 0.37 (0.31-0.45)** | 70 | 120.7 | 0.58 (0.45-0.73)** | 12 | 33.4 | 0.36 (0.19-0.63)** |
| Motor Vehicle Accidents (MVA) | 95 | 238.8 | 0.40 (0.32-0.49)** | 57 | 94.5 | 0.60 (0.46-0.78)** | 12 | 27.2 | 0.44 (0.23-0.77)** |
| All Other Accidents besides MVA | 158 | 397.9 | 0.40 (0.34-0.46)** | 119 | 186.2 | 0.64 (0.53-0.77)** | 17 | 42.9 | 0.40 (0.23-0.64)** |
| Suicides | 146 | 280.5 | 0.52 (0.44-0.61)** | 70 | 122.8 | 0.57 (0.44-0.72)** | 33 | 28.4 | 1.16 (0.80-1.63) |
| Homicides and Legal Intervention | 28 | 116.7 | 0.24 (0.16-0.35)** | 7 | 34.9 | 0.20 (0.08-0.41)** | 9 | 17.7 | 0.51 (0.23-0.96)* |
| Congenital Anomalies | 13 | 20.6 | 0.63 (0.34-1.08) | 3 | 10.6 | 0.28 (0.06-0.83)* | 4 | 2.2 | - |

SMR (95% CI), standardized mortality ratio (95% confidence interval).

▪Expected deaths based on U.S. general population mortality rates.

*Statistically significant at *P* <0.05.

**Statistically significant at *P* <0.01.

MDS, Myelodysplastic Syndrome
