# Supplementary material for: Mortality of 196,826 Men and Women Working in U.S.-Based Petrochemical and Refinery Operations: Update 1979 to 2010
Source: J Occup Environ Med. 2021 Oct 20;64(3):250–62. doi: 10.1097/JOM.0000000000002416 (PMC8887844; doi:10.1097/JOM.0000000000002416)
Supplement: Supplemental Digital Content [file joem-64-0250-s010.docx]

Supplemental Digital Content 6, Table Listing Mortality Results of U.S.-based Petroleum Cohort by Non-manufacturing Job Titles (1979-2010) – WOMEN

| **Cause of Death** | **PROFESSIONALS** | | | **MANAGERS / SUPERVISORS** | | | **OFFICE / CLERICALS** | | |
| --- | --- | --- | --- | --- | --- | --- | --- | --- | --- |
|  | **Observed** | **Expected*** | **SMR (95% CI)** | **Observed** | **Expected*** | **SMR (95% CI)** | **Observed** | **Expected*** | **SMR (95% CI)** |
| All Causes | 859 | 1195.2 | 0.72 (0.67-0.77)** | 124 | 204.5 | 0.61 (0.50-0.72)** | 2704 | 3622.7 | 0.75 (0.72-0.78)** |
| Infectious and Parasitic Diseases | 17 | 36.1 | 0.47 (0.27-0.75)** | 1 | 4.8 | - | 81 | 102.1 | 0.79 (0.63-0.99)* |
| Tuberculosis | 0 | 0.5 | - | 0 | 0.1 | - | 0 | 1.7 | - |
| Human Immunodeficiency Virus (HIV) Disease (incl. AIDS) | 3 | 10.4 | 0.29 (0.06-0.84)* | 0 | 0.6 | - | 15 | 24.6 | 0.61 (0.34-1.01) |
| Malignant Neoplasms (MNs) | 325 | 367.3 | 0.88 (0.79-0.99)* | 58 | 63.5 | 0.91 (0.69-1.18) | 1057 | 1128.3 | 0.94 (0.88-1.00)* |
| MN of Buccal Cavity and Pharynx | 2 | 3.7 | - | 0 | 0.6 | - | 9 | 11.6 | 0.78 (0.36-1.48) |
| MN of Pharynx | 1 | 1.7 | - | 0 | 0.3 | - | 5 | 5.3 | 0.95 (0.31-2.22) |
| MN of Digestive Organs and Peritoneum | 54 | 70.9 | 0.76 (0.57-0.99)* | 12 | 12.7 | 0.94 (0.49-1.65) | 198 | 222.7 | 0.89 (0.77-1.02) |
| MN of Esophagus | 5 | 3.5 | 1.45 (0.47-3.38) | 1 | 0.6 | - | 11 | 11.3 | 0.98 (0.49-1.75) |
| MN of Stomach | 7 | 6.2 | 1.14 (0.46-2.34) | 1 | 1.0 | - | 19 | 18.8 | 1.01 (0.61-1.58) |
| MN of Large Intestine (Colon) | 26 | 26.8 | 0.97 (0.63-1.42) | 5 | 4.8 | 1.04 (0.34-2.42) | 80 | 84.2 | 0.95 (0.75-1.18) |
| MN of Rectum | 5 | 4.5 | 1.11 (0.36-2.58) | 0 | 0.8 | - | 15 | 13.6 | 1.10 (0.62-1.82) |
| MN of Biliary Passages (including Gallbladder)/Liver | 3 | 8.8 | 0.34 (0.07-0.99)* | 2 | 1.6 | - | 22 | 27.7 | 0.79 (0.50-1.20) |
| MN of Liver (Specified Primary or Unspecified) | 0 | 4.2 | - | 0 | 0.7 | - | 11 | 13.4 | 0.82 (0.41-1.47) |
| MN of Pancreas | 6 | 18.0 | 0.33 (0.12-0.72)** | 2 | 3.4 | - | 44 | 57.7 | 0.76 (0.55-1.02) |
| MN of Respiratory System | 83 | 90.5 | 0.92 (0.73-1.14) | 14 | 17.0 | 0.82 (0.45-1.38) | 226 | 290.9 | 0.78 (0.68-0.88)** |
| MN of Nasal Cavity/Mid Ear/Accessory Sinuses | 0 | 0.3 | - | 0 | 0 | - | 2 | 0.9 | - |
| MN of Larynx | 0 | 1.2 | - | 0 | 0.2 | - | 3 | 3.8 | - |
| MN of Bronchus, Trachea, Lung | 82 | 88.6 | 0.92 (0.74-1.15) | 14 | 16.7 | 0.84 (0.46-1.41) | 219 | 285.1 | 0.77 (0.67-0.88)** |
| MN of Bone | 0 | 0.8 | - | 0 | 0.1 | - | 1 | 2.0 | - |
| MN of Connective Tissue | 2 | 3.1 | - | 0 | 0.4 | - | 11 | 8.8 | 1.26 (0.63-2.25) |
| MN of Skin | 6 | 6.1 | 0.98 (0.36-2.14) | 0 | 0.9 | - | 27 | 16.7 | 1.62 (1.07-2.35)* |
| Malignant Melanoma | 6 | 5.3 | 1.12 (0.41-2.45) | 0 | 0.8 | - | 24 | 14.2 | 1.68 (1.08-2.51)* |
| Malignant Mesothelioma | 1 | 0.8 | - | 0 | 0.2 | - | 1 | 2.4 | - |
| MN of Breast | 82 | 73.2 | 1.12 (0.89-1.39) | 10 | 11.4 | 0.87 (0.42-1.61) | 229 | 215.9 | 1.06 (0.93-1.21) |
| MN of Cervix Uteri | 6 | 9.9 | 0.61 (0.22-1.32) | 1 | 1.2 | - | 22 | 26.1 | 0.84 (0.53-1.27) |
| MN of Body of Uterus (including Corpus Uteri) | 6 | 4.3 | 1.41 (0.52-3.06) | 1 | 0.8 | - | 14 | 13.5 | 1.04 (0.57-1.74) |
| MN of Ovary | 13 | 21.6 | 0.60 (0.32-1.03) | 4 | 3.7 | - | 85 | 65.8 | 1.29 (1.03-1.60)* |
| MN of Prostate | 0 | 0 | - | 0 | 0 | - | 0 | 0 | - |
| MN of Testicular | 0 | 0 | - | 0 | 0 | - | 0 | 0 | - |
| MN of Bladder and Other Urinary | 7 | 3.7 | 1.87 (0.75-3.86) | 2 | 0.7 | - | 18 | 12.0 | 1.50 (0.89-2.36) |
| MN of Bladder (Monson) | 7 | 3.6 | 1.95 (0.78-4.02) | 2 | 0.7 | - | 17 | 11.5 | 1.47 (0.86-2.36) |
| MN of Kidney | 6 | 6.0 | 1.00 (0.37-2.17) | 0 | 1.1 | - | 20 | 18.8 | 1.06 (0.65-1.64) |
| MN of Central Nervous System (CNS) including Brain | 14 | 10.2 | 1.38 (0.75-2.31) | 4 | 1.5 | - | 16 | 28.5 | 0.56 (0.32-0.91)* |
| MN of Brain | 14 | 10.0 | 1.41 (0.77-2.36) | 4 | 1.5 | - | 16 | 27.9 | 0.57 (0.33-0.93)* |
| MN of Other/Ill-Defined Sites/Secondary Neoplasms | 14 | 23.5 | 0.60 (0.32-1.00)* | 1 | 4.2 | - | 79 | 73.4 | 1.08 (0.85-1.34) |
| MN of Lymphatic and Hematopoietic Tissue | 23 | 31.2 | 0.74 (0.47-1.11) | 8 | 5.4 | 1.48 (0.64-2.91) | 74 | 95.1 | 0.78 (0.61-0.98)* |
| Hodgkin Lymphoma | 0 | 1.3 | - | 0 | 0.2 | - | 6 | 3.3 | 1.82 (0.67-3.96) |
| Non-Hodgkin Lymphoma | 9 | 11.8 | 0.76 (0.35-1.44) | 2 | 2.1 | - | 23 | 36.7 | 0.63 (0.40-0.94)* |
| Nodular/Follicular Lymphoma | 0 | 0.2 | - | 0 | 0 | - | 1 | 0.5 | - |
| Reticulosarcoma | 0 | 0.7 | - | 0 | 0.1 | - | 0 | 2.0 | - |
| T-Cell Lymphoid Variety | 0 | 0.1 | - | 0 | 0 | - | 1 | 0.2 | - |
| Lymphosarcoma | 1 | 0.3 | - | 0 | 0 | - | 0 | 0.8 | - |
| Other Lymphomas | 7 | 9.9 | 0.71 (0.28-1.46) | 2 | 1.8 | - | 21 | 30.7 | 0.68 (0.42-1.04) |
| Multiple Myeloma | 7 | 5.5 | 1.27 (0.51-2.62) | 1 | 1.1 | - | 14 | 18.2 | 0.77 (0.42-1.29) |
| Leukemia & Aleukemia | 7 | 11.8 | 0.59 (0.24-1.22) | 5 | 1.9 | 2.57 (0.84-6.00) | 28 | 34.8 | 0.81 (0.54-1.16) |
| Acute Lymphocytic Leukemia (ALL) | 0 | 1.0 | - | 0 | 0.1 | - | 2 | 2.4 | - |
| Chronic Lymphocytic Leukemia (CLL) | 1 | 1.5 | - | 1 | 0.3 | - | 4 | 4.9 | - |
| Hairy Cell Leukemia | 0 | 0 | - | 0 | 0 | - | 0 | 0.1 | - |
| Acute Myelocytic Leukemia (AML) | 5 | 4.8 | 1.05 (0.34-2.44) | 2 | 0.8 | - | 13 | 13.8 | 0.94 (0.50-1.61) |
| Chronic Myelocytic Leukemia (CML) | 0 | 1.2 | - | 2 | 0.2 | - | 1 | 3.5 | - |
| Acute Monocytic Leukemia | 0 | 0.1 | - | 0 | 0 | - | 0 | 0.3 | - |
| Chronic Monocytic Leukemia | 0 | 0 | - | 0 | 0 | - | 0 | 0 | - |
| Acute Erythremia and Erythroleukemia | 0 | 0 | - | 0 | 0 | - | 0 | 0.1 | - |
| Megakaryocytic Leukemia | 0 | 0 | - | 0 | 0 | - | 0 | 0 | - |
| Acute Non-Lymphocytic Leukemia (ANLL) | 5 | 4.9 | 1.02 (0.33-2.38) | 2 | 0.8 | - | 13 | 14.2 | 0.92 (0.49-1.57) |
| Other/Unspecified Leukemia (besides ANLL, CML, ALL, CLL) | 1 | 3.2 | - | 0 | 0.6 | - | 8 | 9.8 | 0.82 (0.35-1.61) |
| Benign/In situ/Uncertain Behavior/Unspecified Neoplasms | 9 | 6.2 | 1.46 (0.66-2.76) | 0 | 1.1 | - | 14 | 18.8 | 0.75 (0.41-1.25) |
| Benign CNS (including Brain) | 1 | 0.3 | - | 0 | 0.1 | - | 1 | 1.0 | - |
| Benign Brain | 1 | 0.1 | - | 0 | 0 | - | 0 | 0.2 | - |
| Uncertain Behavior/Unspecified - Brain/Spinal Cord | 4 | 1.8 | - | 0 | 0.3 | - | 6 | 5.4 | 1.12 (0.41-2.44) |
| All Diseases of Blood and Blood-Forming Organs | 2 | 5.8 | 0.34 (0.04-1.24) | 0 | 0.9 | - | 20 | 17.1 | 1.17 (0.71-1.80) |
| Aplastic Anemia | 0 | 0.6 | - | 0 | 0.1 | - | 2 | 1.8 | - |
| All Other Anemias | 1 | 1.8 | - | 0 | 0.3 | - | 5 | 5.4 | 0.93 (0.30-2.17) |
| All Other Diseases of Blood-Forming Organs | 0 | 1.7 | - | 0 | 0.2 | - | 8 | 5.0 | 1.60 (0.69-3.14) |
| Other Specified Diseases of Blood/Blood-Form Org (including MDS) | 1 | 1.9 | - | 0 | 0.4 | - | 4 | 6.2 | 0.64 (0.18-1.65) |
| Endocrine/Nutritional/Metabolic Diseases | 26 | 50.2 | 0.52 (0.34-0.76)** | 2 | 8.7 | 0.23 (0.03-0.83)* | 75 | 155.1 | 0.48 (0.38-0.61)** |
| Diabetes Mellitus | 14 | 36.7 | 0.38 (0.21-0.64)** | 1 | 6.6 | 0.15 (0.01-0.85)* | 49 | 115.9 | 0.42 (0.31-0.56)** |
| Mental Disorders | 25 | 25.8 | 0.97 (0.63-1.43) | 6 | 4.9 | 1.23 (0.45-2.69) | 54 | 80.2 | 0.67 (0.51-0.88)** |
| Alcoholism | 1 | 3.5 | - | 1 | 0.4 | - | 9 | 9.2 | 0.98 (0.45-1.86) |
| Drug Psychosis, Dependence, Poisoning | 9 | 15.5 | 0.58 (0.26-1.10) | 0 | 1.1 | - | 20 | 30.2 | 0.66 (0.41-1.02) |
| Nervous System/Sense Organ Disease | 35 | 44.0 | 0.80 (0.55-1.11) | 7 | 8.2 | 0.85 (0.34-1.76) | 119 | 136.0 | 0.88 (0.72-1.05) |
| Parkinson's Disease | 4 | 4.5 | - | 1 | 1.0 | - | 17 | 14.9 | 1.14 (0.67-1.83) |
| Motor Neuron Disease including Amyotrophic Lateral Sclerosis | 5 | 3.5 | 1.42 (0.46-3.32) | 1 | 0.6 | - | 16 | 11.0 | 1.45 (0.83-2.36) |
| Multiple Sclerosis | 3 | 4.1 | - | 0 | 0.6 | - | 10 | 11.2 | 0.89 (0.43-1.64) |
| Circulatory Disease | 216 | 357.1 | 0.61 (0.53-0.69)** | 24 | 66.6 | 0.36 (0.23-0.54)** | 716 | 1130.5 | 0.63 (0.59-0.68)** |
| All Heart Disease | 165 | 264.7 | 0.62 (0.53-0.73)** | 19 | 49.5 | 0.38 (0.23-0.60)** | 528 | 838.8 | 0.63 (0.58-0.69)** |
| Hypertension with Heart Disease | 8 | 14.1 | 0.57 (0.24-1.12) | 0 | 2.4 | - | 28 | 44.5 | 0.63 (0.42-0.91)* |
| Ischemic Heart Disease | 89 | 167.0 | 0.53 (0.43-0.66)** | 10 | 32.1 | 0.31 (0.15-0.57)** | 348 | 533.8 | 0.65 (0.58-0.72)** |
| Acute Myocardial Infarction | 31 | 72.7 | 0.43 (0.29-0.61)** | 5 | 13.8 | 0.36 (0.12-0.84)* | 139 | 231.9 | 0.60 (0.50-0.71)** |
| Hypertension without Heart Disease | 3 | 8.3 | 0.36 (0.07-1.05) | 0 | 1.6 | - | 14 | 27.1 | 0.52 (0.28-0.87)** |
| Cerebrovascular Disease | 43 | 67.6 | 0.64 (0.46-0.86)** | 3 | 12.5 | 0.24 (0.05-0.70)** | 143 | 213.3 | 0.67 (0.56-0.79)** |
| Diseases of Arteries/Veins/Other Circulatory | 5 | 16.5 | 0.30 (0.10-0.71)** | 2 | 3.0 | - | 31 | 51.4 | 0.60 (0.41-0.86)** |
| Aortic Aneurysm | 0 | 5.1 | - | 2 | 1.0 | - | 11 | 16.3 | 0.68 (0.34-1.21) |
| Non-Malignant Respiratory Disease | 71 | 100.3 | 0.71 (0.55-0.89)** | 12 | 19.3 | 0.62 (0.32-1.08) | 198 | 320.9 | 0.62 (0.53-0.71)** |
| Acute Respiratory Infections except Influenza/Pneumonia | 1 | 0.3 | - | 0 | 0 | - | 0 | 0.8 | - |
| Pneumonia | 13 | 23.1 | 0.56 (0.30-0.96)* | 0 | 4.2 | - | 42 | 71.0 | 0.59 (0.43-0.80)** |
| Influenza | 0 | 0.6 | - | 0 | 0.1 | - | 1 | 1.6 | - |
| Bronchitis, Emphysema, and Asthma | 10 | 12.8 | 0.78 (0.38-1.44) | 1 | 2.3 | - | 35 | 40.0 | 0.88 (0.61-1.22) |
| Bronchitis | 0 | 0.9 | - | 1 | 0.2 | - | 3 | 2.8 | - |
| Emphysema | 9 | 7.5 | 1.19 (0.54-2.26) | 0 | 1.5 | - | 20 | 24.9 | 0.80 (0.49-1.24) |
| Asthma | 1 | 4.3 | - | 0 | 0.6 | - | 12 | 12.3 | 0.98 (0.51-1.71) |
| Pneumoconiosis and Other Respiratory Diseases | 47 | 63.6 | 0.74 (0.54-0.98)* | 11 | 12.8 | 0.86 (0.43-1.54) | 120 | 207.5 | 0.58 (0.48-0.69)** |
| Chronic Obstructive Pulmonary Disease | 36 | 46.2 | 0.78 (0.55-1.08) | 7 | 9.5 | 0.73 (0.30-1.51) | 86 | 152.9 | 0.56 (0.45-0.70)** |
| Pneumoconiosis/Other Lung Diseases, External Agents | 4 | 4.6 | - | 0 | 0.9 | - | 9 | 14.4 | 0.62 (0.29-1.18) |
| Asbestosis | 0 | 0 | - | 0 | 0 | - | 0 | 0.1 | - |
| Silicosis and Anthracosilicosis | 0 | 0 | - | 0 | 0 | - | 0 | 0 | - |
| Digestive Disease | 28 | 50.8 | 0.55 (0.37-0.80)** | 4 | 8.1 | 0.49 (0.13-1.26) | 91 | 150.2 | 0.61 (0.49-0.74)** |
| Ulcer of Stomach and Duodenum | 0 | 2.0 | - | 0 | 0.4 | - | 4 | 6.2 | 0.65 (0.18-1.65) |
| Cirrhosis of Liver | 12 | 20.3 | 0.59 (0.31-1.03) | 1 | 2.9 | - | 37 | 57.4 | 0.64 (0.45-0.89)** |
| Genitourinary Disease | 17 | 23.7 | 0.72 (0.42-1.15) | 2 | 4.4 | - | 49 | 75.8 | 0.65 (0.48-0.86)** |
| Nephritis and Nephrosis | 13 | 16.8 | 0.78 (0.41-1.33) | 1 | 3.1 | - | 35 | 53.7 | 0.65 (0.45-0.91)** |
| Skin/Subcutaneous Tissue Disease | 1 | 1.9 | - | 0 | 0.3 | - | 7 | 5.8 | 1.22 (0.49-2.51) |
| Musculoskeletal Disease & Connective Tissue | 4 | 10.0 | 0.40 (0.11-1.02) | 0 | 1.6 | - | 19 | 29.7 | 0.64 (0.38-1.00)* |
| All External Causes of Death | 66 | 95.4 | 0.69 (0.54-0.88)** | 7 | 9.4 | 0.74 (0.30-1.53) | 152 | 218.6 | 0.70 (0.59-0.82)** |
| Accidents | 42 | 60.4 | 0.70 (0.50-0.94)* | 4 | 6.4 | 0.63 (0.17-1.61) | 81 | 141.3 | 0.57 (0.46-0.71)** |
| Transportation Accidents | 22 | 29.0 | 0.76 (0.48-1.15) | 2 | 2.7 | - | 38 | 65.1 | 0.58 (0.41-0.80)** |
| Motor Vehicle Accidents (MVA) | 15 | 23.2 | 0.65 (0.36-1.06) | 2 | 2.2 | - | 31 | 53.4 | 0.58 (0.40-0.82)** |
| All Other Accidents besides MVA | 27 | 36.7 | 0.74 (0.48-1.07) | 2 | 4.1 | - | 50 | 86.8 | 0.58 (0.43-0.76)** |
| Suicides | 14 | 19.0 | 0.74 (0.40-1.24) | 2 | 1.8 | - | 39 | 41.1 | 0.95 (0.67-1.30) |
| Homicides and Legal Intervention | 7 | 11.1 | 0.63 (0.25-1.30) | 1 | 0.8 | - | 23 | 25.1 | 0.92 (0.58-1.38) |
| Congenital Anomalies | 3 | 3.9 | - | 0 | 0.4 | - | 5 | 9.4 | 0.53 (0.17-1.24) |

SMR (95% CI), standardized mortality ratio (95% confidence interval).

▪Expected deaths based on U.S. general population mortality rates.

*Statistically significant at *P* <0.05.

**Statistically significant at *P* <0.01.

MDS, Myelodysplastic Syndrome
