# Supplementary material for: Mortality of 196,826 Men and Women Working in U.S.-Based Petrochemical and Refinery Operations: Update 1979 to 2010
Source: J Occup Environ Med. 2021 Oct 20;64(3):250–62. doi: 10.1097/JOM.0000000000002416 (PMC8887844; doi:10.1097/JOM.0000000000002416)
Supplement: Supplemental Digital Content [file joem-64-0250-s011.docx]

Supplemental Digital Content 7, Table Listing Mortality Results of U.S.-based Petroleum Cohort by Other Operating Segments (1979-2010) – WOMEN

| **Cause of Death** | **COAL AND MINERALS** | | | **CORPORATE GLOBAL SERVICES** | | |
| --- | --- | --- | --- | --- | --- | --- |
|  | **Observed** | **Expected▪** | **SMR (95% CI)** | **Observed** | **Expected▪** | **SMR (95% CI)** |
| All Causes | 97 | 100.3 | 0.97 (0.78-1.18) | 764 | 943.1 | 0.81 (0.75-0.87)** |
| Infectious and Parasitic diseases | 0 | 2.9 | - | 22 | 26.1 | 0.84 (0.53-1.28) |
| Tuberculosis | 0 | 0 | - | 0 | 0.4 | - |
| Human Immunodeficiency Virus (HIV) Disease (incl. AIDS) | 0 | 0.7 | - | 6 | 6.1 | 0.98 (0.36-2.14) |
| Malignant Neoplasms (MNs) | 40 | 34.3 | 1.16 (0.83-1.59) | 303 | 286.6 | 1.06 (0.94-1.18) |
| MN of Buccal Cavity and Pharynx | 0 | 0.3 | - | 1 | 3.0 | - |
| MN of Pharynx | 0 | 0.2 | - | 0 | 1.4 | - |
| MN of Digestive Organs and Peritoneum | 6 | 6.4 | 0.94 (0.35-2.06) | 59 | 57.7 | 1.02 (0.78-1.32) |
| MN of Esophagus | 0 | 0.3 | - | 6 | 3.0 | 2.03 (0.74-4.42) |
| MN of Stomach | 0 | 0.5 | - | 7 | 4.9 | 1.42 (0.57-2.92) |
| MN of Large Intestine (Colon) | 4 | 2.3 | - | 21 | 21.9 | 0.96 (0.59-1.46) |
| MN of Rectum | 0 | 0.4 | - | 5 | 3.5 | 1.43 (0.46-3.34) |
| MN of Biliary Passages (including Gallbladder)/Liver | 0 | 0.8 | - | 7 | 7.1 | 0.98 (0.39-2.02) |
| MN of Liver (Specified Primary or Unspecified) | 0 | 0.4 | - | 4 | 3.5 | - |
| MN of Pancreas | 2 | 1.7 | - | 11 | 14.9 | 0.74 (0.37-1.32) |
| MN of Respiratory System | 6 | 8.8 | 0.68 (0.25-1.49) | 69 | 73.5 | 0.94 (0.73-1.19) |
| MN of Nasal Cavity/Mid Ear/Accessory Sinuses | 0 | 0 | - | 0 | 0.2 | - |
| MN of Larynx | 0 | 0.1 | - | 1 | 1.0 | - |
| MN of Bronchus, Trachea, Lung | 6 | 8.6 | 0.70 (0.26-1.52) | 67 | 72 | 0.93 (0.72-1.18) |
| MN of Bone | 0 | 0.1 | - | 1 | 0.5 | - |
| MN of Connective Tissue | 0 | 0.3 | - | 1 | 2.2 | - |
| MN of Skin | 1 | 0.6 | - | 4 | 4.1 | - |
| Malignant Melanoma | 1 | 0.5 | - | 4 | 3.5 | - |
| Malignant Mesothelioma | 0 | 0 | - | 0 | 0.6 | - |
| MN of Breast | 8 | 7.0 | 1.14 (0.49-2.25) | 71 | 54.1 | 1.31 (1.03-1.66)* |
| MN of Cervix Uteri | 1 | 0.9 | - | 5 | 6.5 | 0.77 (0.25-1.79) |
| MN of Body of Uterus (including Corpus Uteri) | 0 | 0.4 | - | 3 | 3.5 | - |
| MN of Ovary | 2 | 2.1 | - | 22 | 16.5 | 1.33 (0.84-2.02) |
| MN of Prostate | 0 | 0 | - | 0 | 0 | - |
| MN of Testicular | 0 | 0 | - | 0 | 0 | - |
| MN of Bladder and Other Urinary | 1 | 0.3 | - | 10 | 3.2 | 3.17 (1.52-5.82)** |
| MN of Bladder (Monson) | 1 | 0.3 | - | 9 | 3.0 | 2.97 (1.36-5.64)** |
| MN of Kidney | 1 | 0.6 | - | 2 | 4.8 | - |
| MN of Central Nervous System (CNS) including Brain | 2 | 1.0 | - | 6 | 7.0 | 0.85 (0.31-1.86) |
| MN of Brain | 2 | 0.9 | - | 6 | 6.9 | 0.87 (0.32-1.89) |
| MN of Other/Ill-Defined Sites/Secondary Neoplasms | 4 | 2.1 | - | 14 | 18.8 | 0.74 (0.41-1.25) |
| MN of Lymphatic and Hematopoietic Tissue | 8 | 2.8 | 2.89 (1.25-5.69)* | 25 | 24.4 | 1.03 (0.66-1.52) |
| Hodgkin Lymphoma | 0 | 0.1 | - | 2 | 0.8 | - |
| Non-Hodgkin Lymphoma | 3 | 1.0 | - | 8 | 9.4 | 0.85 (0.37-1.68) |
| Nodular/Follicular Lymphoma | 0 | 0 | - | 0 | 0.1 | - |
| Reticulosarcoma | 0 | 0.1 | - | 1 | 0.5 | - |
| T-Cell Lymphoid Variety | 0 | 0 | - | 1 | 0.1 | - |
| Lymphosarcoma | 0 | 0 | - | 0 | 0.2 | - |
| Other Lymphomas | 3 | 0.9 | - | 7 | 7.9 | 0.89 (0.36-1.83) |
| Multiple Myeloma | 4 | 0.5 | - | 4 | 4.8 | - |
| Leukemia & Aleukemia | 1 | 1.0 | - | 9 | 8.9 | 1.02 (0.46-1.93) |
| Acute Lymphocytic Leukemia (ALL) | 0 | 0.1 | - | 1 | 0.6 | - |
| Chronic Lymphocytic Leukemia (CLL) | 1 | 0.1 | - | 2 | 1.3 | - |
| Hairy Cell Leukemia | 0 | 0 | - | 0 | 0 | - |
| Acute Non-Lymphocytic Leukemia (ANLL) | 0 | 0.4 | - | 5 | 3.6 | 1.41 (0.46-3.28) |
| Acute Myelocytic Leukemia (AML) | 0 | 0.4 | - | 5 | 3.5 | 1.44 (0.47-3.37) |
| Chronic Myelocytic Leukemia (CML) | 0 | 0.1 | - | 0 | 0.9 | - |
| Acute Monocytic Leukemia | 0 | 0 | - | 0 | 0.1 | - |
| Chronic Monocytic Leukemia | 0 | 0 | - | 0 | 0 | - |
| Acute Erythremia and Erythroleukemia | 0 | 0 | - | 0 | 0 | - |
| Megakaryocytic Leukemia | 0 | 0 | - | 0 | 0 | - |
| Other/Unspecified Leukemia (besides ANLL, CML, ALL, CLL) | 0 | 0.3 | - | 1 | 2.5 | - |
| Benign/In situ/Uncertain Behavior/Unspecified Neoplasms | 0 | 0.5 | - | 5 | 4.9 | 1.03 (0.33-2.40) |
| Benign CNS (including Brain) | 0 | 0 | - | 1 | 0.3 | - |
| Benign Brain | 0 | 0 | - | 0 | 0.1 | - |
| Uncertain Behavior/Unspecified - Brain/Spinal Cord | 0 | 0.2 | - | 2 | 1.4 | - |
| All Diseases of Blood and Blood-Forming Organs | 1 | 0.5 | - | 1 | 4.5 | - |
| Aplastic Anemia | 0 | 0.1 | - | 0 | 0.5 | - |
| All Other Anemias | 1 | 0.1 | - | 0 | 1.4 | - |
| All Other Diseases of Blood-Forming Organs | 0 | 0.1 | - | 0 | 1.3 | - |
| Other Specified Diseases of Blood/Blood-Form Org (including MDS) | 0 | 0.2 | - | 1 | 1.6 | - |
| Endocrine/Nutritional/Metabolic Diseases | 3 | 4.4 | - | 13 | 40.7 | 0.32 (0.17-0.55)** |
| Diabetes Mellitus | 2 | 3.2 | - | 10 | 30.5 | 0.33 (0.16-0.60)** |
| Mental Disorders | 2 | 1.8 | - | 15 | 21.6 | 0.70 (0.39-1.15) |
| Alcoholism | 1 | 0.3 | - | 4 | 2.3 | - |
| Drug Psychosis, Dependence, Poisoning | 0 | 1.1 | - | 6 | 7.5 | 0.80 (0.29-1.73) |
| Nervous System/Sense Organ Disease | 7 | 3.4 | 2.08 (0.84-4.28) | 26 | 35.9 | 0.72 (0.47-1.06) |
| Parkinson's Disease | 0 | 0.3 | - | 4 | 4.0 | - |
| Motor Neuron Disease including Amyotrophic Lateral Sclerosis | 1 | 0.3 | - | 2 | 2.8 | - |
| Multiple Sclerosis | 0 | 0.4 | - | 2 | 2.7 | - |
| Circulatory Disease | 20 | 27.5 | 0.73 (0.44-1.12) | 201 | 303.5 | 0.66 (0.57-0.76)** |
| All Heart Disease | 16 | 20.4 | 0.78 (0.45-1.27) | 154 | 225.1 | 0.68 (0.58-0.80)** |
| Hypertension with Heart Disease | 1 | 1.1 | - | 8 | 12.1 | 0.66 (0.29-1.31) |
| Ischemic Heart Disease | 12 | 12.8 | 0.94 (0.48-1.64) | 102 | 143.4 | 0.71 (0.58-0.86)** |
| Acute Myocardial Infarction | 8 | 5.7 | 1.40 (0.60-2.76) | 34 | 62.2 | 0.55 (0.38-0.76)** |
| Hypertension without Heart Disease | 0 | 0.6 | - | 3 | 7.4 | 0.41 (0.08-1.19) |
| Cerebrovascular Disease | 4 | 5.2 | 0.77 (0.21-1.98) | 40 | 57.3 | 0.70 (0.50-0.95)* |
| Diseases of Arteries/Veins/Other Circulatory | 0 | 1.3 | - | 4 | 13.7 | 0.29 (0.08-0.75)** |
| Aortic Aneurysm | 0 | 0.4 | - | 2 | 4.3 | - |
| Non-Malignant Respiratory Disease | 6 | 8.3 | 0.72 (0.26-1.57) | 67 | 83.6 | 0.80 (0.62-1.02) |
| Acute Respiratory Infections except Influenza/Pneumonia | 0 | 0 | - | 0 | 0.2 | - |
| Pneumonia | 0 | 1.7 | - | 14 | 19.0 | 0.74 (0.40-1.23) |
| Influenza | 0 | 0 | - | 0 | 0.4 | - |
| Bronchitis, Emphysema, and Asthma | 2 | 1.1 | - | 11 | 10.3 | 1.07 (0.53-1.91) |
| Bronchitis | 0 | 0.1 | - | 0 | 0.7 | - |
| Emphysema | 2 | 0.7 | - | 7 | 6.4 | 1.09 (0.44-2.24) |
| Asthma | 0 | 0.4 | - | 4 | 3.2 | - |
| Pneumoconiosis and Other Respiratory Diseases | 4 | 5.4 | 0.73 (0.20-1.88) | 42 | 53.6 | 0.78 (0.56-1.06) |
| Chronic Obstructive Pulmonary Disease | 3 | 4.0 | - | 33 | 39.3 | 0.84 (0.58-1.18) |
| Pneumoconiosis/Other Lung Diseases, External Agents | 0 | 0.3 | - | 3 | 3.9 | - |
| Asbestosis | 0 | 0 | - | 0 | 0 | - |
| Silicosis and Anthracosilicosis | 0 | 0 | - | 0 | 0 | - |
| Digestive Disease | 2 | 4.5 | - | 29 | 38.5 | 0.75 (0.51-1.08) |
| Ulcer of Stomach and Duodenum | 0 | 0.2 | - | 2 | 1.6 | - |
| Cirrhosis of Liver | 2 | 1.9 | - | 13 | 14.3 | 0.91 (0.48-1.56) |
| Genitourinary Disease | 2 | 1.9 | - | 16 | 20.3 | 0.79 (0.45-1.28) |
| Nephritis and Nephrosis | 2 | 1.4 | - | 13 | 14.3 | 0.91 (0.48-1.55) |
| Skin/Subcutaneous Tissue Disease | 0 | 0.2 | - | 1 | 1.5 | - |
| Musculoskeletal Disease & Connective Tissue | 0 | 0.8 | - | 6 | 7.6 | 0.78 (0.29-1.71) |
| All External Causes of Death | 14 | 7.8 | 1.81 (0.99-3.03) | 45 | 54.1 | 0.83 (0.61-1.11) |
| Accidents | 7 | 4.8 | 1.45 (0.58-2.98) | 25 | 35.3 | 0.71 (0.46-1.04) |
| Transportation Accidents | 5 | 2.4 | 2.07 (0.67-4.84) | 16 | 15.9 | 1.01 (0.58-1.64) |
| Motor Vehicle Accidents (MVA) | 5 | 2.0 | 2.52 (0.82-5.87) | 15 | 12.8 | 1.17 (0.65-1.93) |
| All Other Accidents besides MVA | 2 | 2.8 | - | 10 | 22.2 | 0.45 (0.22-0.83)** |
| Suicides | 3 | 1.7 | - | 12 | 9.9 | 1.21 (0.62-2.11) |
| Homicides and Legal Intervention | 3 | 0.8 | - | 7 | 6.1 | 1.15 (0.46-2.36) |
| Congenital Anomalies | 0 | 0.3 | - | 0 | 2.3 | - |

SMR (95% CI), standardized mortality ratio (95% confidence interval).

▪Expected deaths based on U.S. general population mortality rates.

*Statistically significant at *P* <0.05.

**Statistically significant at *P* <0.01.

MDS, Myelodysplastic Syndrome
