# Supplementary material for: Mortality of 196,826 Men and Women Working in U.S.-Based Petrochemical and Refinery Operations: Update 1979 to 2010
Source: J Occup Environ Med. 2021 Oct 20;64(3):250–62. doi: 10.1097/JOM.0000000000002416 (PMC8887844; doi:10.1097/JOM.0000000000002416)
Supplement: Supplemental Digital Content [file joem-64-0250-s012.docx]

Supplemental Digital Content 8, Table List Mortality Results of U.S.-based Petroleum Cohort by Main Operating Segments (1979-2010) – WOMEN

| **Cause of Death** | **DOWNSTREAM** | | | **UPSTREAM** | | | **CHEMICALS** | | |
| --- | --- | --- | --- | --- | --- | --- | --- | --- | --- |
|  | **Observed** | **Expected▪** | **SMR (95% CI)** | **Observed** | **Expected▪** | **SMR (95% CI)** | **Observed** | **Expected▪** | **SMR (95% CI)** |
| All Causes | 1284 | 1618.1 | 0.79 (0.75-0.84)** | 662 | 923.5 | 0.72 (0.66-0.77)** | 934 | 1038.9 | 0.90 (0.84-0.96)** |
| Infectious and Parasitic Diseases | 42 | 46.6 | 0.90 (0.65-1.22) | 11 | 27.1 | 0.41 (0.20-0.73)** | 24 | 32.0 | 0.75 (0.48-1.12) |
| Tuberculosis | 0 | 0.7 | - | 0 | 0.4 | - | 0 | 0.5 | - |
| Human Immunodeficiency Virus (HIV) Disease (incl. AIDS) | 7 | 12.2 | 0.58 (0.23-1.18) | 0 | 7.0 | - | 5 | 9.2 | 0.54 (0.18-1.27) |
| Malignant Neoplasms (MNs) | 475 | 491.5 | 0.97 (0.88-1.06) | 247 | 293.1 | 0.84 (0.74-0.96)** | 324 | 335.4 | 0.97 (0.86-1.08) |
| MN of Buccal Cavity and Pharynx | 2 | 5.0 | 0.40 (0.05-1.43) | 2 | 3.0 | - | 6 | 3.4 | 1.77 (0.65-3.86) |
| MN of Pharynx | 1 | 2.3 | - | 1 | 1.3 | - | 3 | 1.6 | - |
| MN of Digestive Organs and Peritoneum | 98 | 97.4 | 1.01 (0.82-1.23) | 47 | 56.6 | 0.83 (0.61-1.10) | 53 | 64.3 | 0.83 (0.62-1.08) |
| MN of Esophagus | 6 | 4.9 | 1.23 (0.45-2.68) | 2 | 2.8 | - | 5 | 3.2 | 1.54 (0.50-3.60) |
| MN of Stomach | 12 | 8.3 | 1.44 (0.74-2.52) | 4 | 4.8 | - | 1 | 5.6 | 0.18 (0.01-1.00) |
| MN of Large Intestine (Colon) | 39 | 37.0 | 1.05 (0.75-1.44) | 21 | 21.2 | 0.99 (0.61-1.51) | 21 | 24.0 | 0.87 (0.54-1.34) |
| MN of Rectum | 7 | 6.0 | 1.17 (0.47-2.41) | 4 | 3.5 | - | 4 | 4.0 | - |
| MN of Biliary Passages (including Gallbladder)/Liver | 9 | 12.1 | 0.75 (0.34-1.42) | 3 | 7.1 | 0.42 (0.09-1.23) | 7 | 8.1 | 0.87 (0.35-1.79) |
| MN of Liver (Specified Primary or Unspecified) | 4 | 5.8 | 0.68 (0.19-1.75) | 1 | 3.4 | - | 5 | 3.9 | 1.28 (0.42-2.98) |
| MN of Pancreas | 18 | 25.0 | 0.72 (0.43-1.14) | 12 | 14.6 | 0.82 (0.42-1.44) | 13 | 16.5 | 0.79 (0.42-1.34) |
| MN of Respiratory System | 119 | 124.8 | 0.95 (0.79-1.14) | 50 | 74.2 | 0.67 (0.50-0.89)** | 73 | 85.1 | 0.86 (0.67-1.08) |
| MN of Nasal Cavity/Mid Ear/Accessory Sinuses | 0 | 0.4 | - | 2 | 0.2 | - | 0 | 0.3 | - |
| MN of Larynx | 1 | 1.7 | - | 0 | 1.0 | - | 0 | 1.1 | - |
| MN of Bronchus, Trachea, Lung | 117 | 122.2 | 0.96 (0.79-1.15) | 48 | 72.7 | 0.66 (0.49-0.88)** | 73 | 83.3 | 0.88 (0.69-1.10) |
| MN of Bone | 0 | 0.9 | - | 0 | 0.6 | - | 0 | 0.6 | - |
| MN of Connective Tissue | 5 | 3.9 | 1.28 (0.42-3.00) | 0 | 2.4 | - | 5 | 2.8 | 1.79 (0.58-4.18) |
| MN of Skin | 8 | 7.4 | 1.08 (0.47-2.14) | 7 | 4.6 | 1.51 (0.61-3.11) | 7 | 5.3 | 1.32 (0.53-2.73) |
| Malignant Melanoma | 7 | 6.3 | 1.11 (0.45-2.29) | 5 | 4.0 | 1.24 (0.40-2.90) | 7 | 4.6 | 1.52 (0.61-3.14) |
| Malignant Mesothelioma | 2 | 1.1 | - | 1 | 0.6 | - | 0 | 0.7 | - |
| MN of Breast | 101 | 94.3 | 1.07 (0.87-1.30) | 50 | 57.8 | 0.86 (0.64-1.14) | 72 | 66.8 | 1.08 (0.84-1.36) |
| MN of Cervix Uteri | 7 | 11.7 | 0.60 (0.24-1.23) | 10 | 7.4 | 1.35 (0.65-2.48) | 8 | 8.8 | 0.91 (0.39-1.80) |
| MN of Body of Uterus (including Corpus Uteri) | 7 | 5.9 | 1.20 (0.48-2.46) | 6 | 3.4 | 1.74 (0.64-3.79) | 1 | 3.9 | - |
| MN of Ovary | 29 | 28.5 | 1.02 (0.68-1.46) | 20 | 17.3 | 1.16 (0.71-1.79) | 20 | 19.8 | 1.01 (0.62-1.56) |
| MN of Prostate | 0 | 0 | - | 0 | 0 | - | 0 | 0 | - |
| MN of Testicular | 0 | 0 | - | 0 | 0 | - | 0 | 0 | - |
| MN of Bladder and Other Urinary | 8 | 5.3 | 1.51 (0.65-2.97) | 0 | 3.0 | - | 6 | 3.3 | 1.82 (0.67-3.95) |
| MN of Bladder (Monson) | 8 | 5.1 | 1.57 (0.68-3.10) | 0 | 2.8 | - | 6 | 3.2 | 1.90 (0.70-4.13) |
| MN of Kidney | 8 | 8.2 | 0.98 (0.42-1.93) | 7 | 4.8 | 1.45 (0.58-2.99) | 9 | 5.5 | 1.64 (0.75-3.11) |
| MN of Central Nervous System (CNS) including Brain | 9 | 12.5 | 0.72 (0.33-1.36) | 8 | 7.8 | 1.02 (0.44-2.02) | 8 | 9.0 | 0.89 (0.38-1.75) |
| MN of Brain | 9 | 12.3 | 0.73 (0.34-1.39) | 8 | 7.7 | 1.04 (0.45-2.06) | 7 | 8.8 | 0.79 (0.32-1.63) |
| MN of Other/Ill-Defined Sites/Secondary Neoplasms | 30 | 32.1 | 0.94 (0.63-1.34) | 20 | 18.8 | 1.07 (0.65-1.65) | 28 | 21.3 | 1.31 (0.87-1.90) |
| MN of Lymphatic and Hematopoietic Tissue | 31 | 42.0 | 0.74 (0.50-1.05) | 16 | 24.4 | 0.66 (0.38-1.06) | 24 | 27.7 | 0.87 (0.56-1.29) |
| Hodgkin Lymphoma | 2 | 1.5 | - | 2 | 0.9 | - | 1 | 1.1 | - |
| Non-Hodgkin Lymphoma | 13 | 16.2 | 0.80 (0.43-1.37) | 3 | 9.3 | 0.32 (0.07-0.94)* | 11 | 10.5 | 1.05 (0.52-1.88) |
| Nodular/Follicular Lymphoma | 1 | 0.2 | - | 0 | 0.1 | - | 0 | 0.1 | - |
| Reticulosarcoma | 0 | 0.9 | - | 0 | 0.5 | - | 0 | 0.6 | - |
| T-Cell Lymphoid Variety | 0 | 0.1 | - | 0 | 0.1 | - | 0 | 0.1 | - |
| Lymphosarcoma | 0 | 0.4 | - | 0 | 0.2 | - | 1 | 0.2 | - |
| Other Lymphomas | 11 | 13.5 | 0.81 (0.41-1.45) | 2 | 7.8 | 0.26 (0.03-0.93)* | 9 | 8.8 | 1.03 (0.47-1.95) |
| Multiple Myeloma | 3 | 7.9 | 0.38 (0.08-1.11) | 4 | 4.5 | - | 2 | 5.1 | 0.39 (0.05-1.40) |
| Leukemia & Aleukemia | 12 | 15.5 | 0.78 (0.40-1.36) | 7 | 9.1 | 0.77 (0.31-1.59) | 10 | 10.3 | 0.97 (0.46-1.78) |
| Acute Lymphocytic Leukemia (ALL) | 0 | 1.1 | - | 1 | 0.7 | - | 2 | 0.8 | - |
| Chronic Lymphocytic Leukemia (CLL) | 2 | 2.2 | - | 0 | 1.2 | - | 2 | 1.3 | - |
| Hairy Cell Leukemia | 0 | 0 | - | 0 | 0 | - | 0 | 0 | - |
| Acute Myelocytic Leukemia (AML) | 5 | 6.1 | 0.82 (0.27-1.91) | 3 | 3.7 | - | 3 | 4.2 | - |
| Chronic Myelocytic Leukemia (CML) | 2 | 1.6 | - | 1 | 0.9 | - | 1 | 1.1 | - |
| Acute Monocytic Leukemia | 0 | 0.1 | - | 0 | 0.1 | - | 0 | 0.1 | - |
| Chronic Monocytic Leukemia | 0 | 0 | - | 0 | 0 | - | 0 | 0 | - |
| Acute Erythremia and Erythroleukemia | 0 | 0 | - | 0 | 0 | - | 0 | 0 | - |
| Megakaryocytic Leukemia | 0 | 0 | - | 0 | 0 | - | 0 | 0 | - |
| Acute Non-Lymphocytic Leukemia (ANLL) | 5 | 6.3 | 0.80 (0.26-1.86) | 3 | 3.8 | - | 3 | 4.3 | - |
| Other/Unspecified Leukemia (besides ANLL, CML, ALL, CLL) | 3 | 4.4 | - | 2 | 2.5 | - | 2 | 2.8 | - |
| Benign/In situ/Uncertain Behavior/Unspecified Neoplasms | 8 | 8.4 | 0.96 (0.41-1.88) | 2 | 4.8 | - | 3 | 5.4 | 0.56 (0.12-1.64) |
| Benign CNS (including Brain) | 1 | 0.4 | - | 1 | 0.2 | - | 0 | 0.3 | - |
| Benign Brain | 0 | 0.1 | - | 1 | 0.1 | - | 0 | 0.1 | - |
| Uncertain Behavior/Unspecified - Brain/Spinal Cord | 4 | 2.4 | - | 0 | 1.4 | - | 2 | 1.6 | - |
| All Diseases of Blood and Blood-Forming Organs | 9 | 7.8 | 1.16 (0.53-2.19) | 8 | 4.4 | 1.81 (0.78-3.58) | 5 | 5.1 | 0.98 (0.32-2.28) |
| Aplastic Anemia | 1 | 0.8 | - | 1 | 0.5 | - | 0 | 0.5 | - |
| All Other Anemias | 1 | 2.5 | - | 3 | 1.3 | - | 3 | 1.5 | - |
| All Other Diseases of Blood-Forming Organs | 4 | 2.3 | - | 2 | 1.3 | - | 2 | 1.6 | - |
| Other Specified Diseases of Blood/Blood-Form Org (including MDS) | 2 | 2.8 | - | 0 | 1.5 | - | 1 | 1.6 | - |
| Endocrine/Nutritional/Metabolic Diseases | 37 | 68.6 | 0.54 (0.38-0.74)** | 19 | 39.6 | 0.48 (0.29-0.75)** | 28 | 45.0 | 0.62 (0.41-0.90)** |
| Diabetes Mellitus | 21 | 50.9 | 0.41 (0.26-0.63)** | 12 | 29.3 | 0.41 (0.21-0.72)** | 25 | 33.4 | 0.75 (0.48-1.10) |
| Mental Disorders | 24 | 37.2 | 0.64 (0.41-0.96)* | 16 | 19.5 | 0.82 (0.47-1.33) | 15 | 20.2 | 0.74 (0.42-1.22) |
| Alcoholism | 4 | 4.1 | - | 4 | 2.7 | - | 1 | 3.1 | - |
| Drug Psychosis, Dependence, Poisoning | 9 | 15.2 | 0.59 (0.27-1.12) | 12 | 9.8 | 1.22 (0.63-2.13) | 10 | 12.1 | 0.83 (0.40-1.52) |
| Nervous System/Sense Organ Disease | 51 | 61.8 | 0.82 (0.61-1.08) | 32 | 33.7 | 0.95 (0.65-1.34) | 30 | 36.1 | 0.83 (0.56-1.19) |
| Parkinson's Disease | 7 | 6.8 | 1.04 (0.42-2.13) | 4 | 3.5 | - | 5 | 3.6 | 1.39 (0.45-3.25) |
| Motor Neuron Disease including Amyotrophic Lateral Sclerosis | 11 | 4.7 | 2.32 (1.16-4.16)* | 2 | 2.9 | - | 6 | 3.3 | 1.84 (0.68-4.01) |
| Multiple Sclerosis | 3 | 4.9 | - | 5 | 3.2 | 1.56 (0.51-3.64) | 1 | 3.7 | - |
| Circulatory Disease | 343 | 508.0 | 0.68 (0.61-0.75)** | 159 | 275.6 | 0.58 (0.49-0.67)** | 248 | 303.2 | 0.82 (0.72-0.93)** |
| All Heart Disease | 262 | 376.5 | 0.70 (0.61-0.78)** | 118 | 204.3 | 0.58 (0.48-0.69)** | 168 | 224.9 | 0.75 (0.64-0.87)** |
| Hypertension with Heart Disease | 9 | 20.0 | 0.45 (0.21-0.86)* | 4 | 11.1 | 0.36 (0.10-0.92)* | 11 | 12.6 | 0.87 (0.44-1.56) |
| Ischemic Heart Disease | 168 | 239.2 | 0.70 (0.60-0.82)** | 69 | 129.0 | 0.54 (0.42-0.68)** | 100 | 141.3 | 0.71 (0.58-0.86)** |
| Acute Myocardial Infarction | 71 | 103.4 | 0.69 (0.54-0.87)** | 25 | 56.3 | 0.44 (0.29-0.66)** | 38 | 62.3 | 0.61 (0.43-0.84)** |
| Hypertension without Heart Disease | 9 | 12.2 | 0.74 (0.34-1.40) | 0 | 6.6 | - | 8 | 7.2 | 1.10 (0.48-2.18) |
| Cerebrovascular Disease | 60 | 96.1 | 0.62 (0.48-0.80)** | 33 | 52.0 | 0.63 (0.44-0.89)** | 56 | 57.1 | 0.98 (0.74-1.27) |
| Diseases of Arteries/Veins/Other Circulatory | 12 | 23.1 | 0.52 (0.27-0.91)* | 8 | 12.6 | 0.64 (0.27-1.25) | 16 | 13.9 | 1.15 (0.66-1.87) |
| Aortic Aneurysm | 5 | 7.3 | 0.69 (0.22-1.61) | 1 | 4.0 | - | 5 | 4.4 | 1.14 (0.37-2.67) |
| Non-Malignant Respiratory Disease | 90 | 142.2 | 0.63 (0.51-0.78)** | 48 | 79 | 0.61 (0.45-0.81)** | 87 | 87.2 | 1.00 (0.80-1.23) |
| Acute Respiratory Infections except Influenza/Pneumonia | 1 | 0.4 | - | 0 | 0.2 | - | 0 | 0.2 | - |
| Pneumonia | 11 | 32.3 | 0.34 (0.17-0.61)** | 8 | 17.4 | 0.46 (0.20-0.91)* | 15 | 18.9 | 0.79 (0.44-1.31) |
| Influenza | 1 | 0.7 | - | 0 | 0.4 | - | 0 | 0.5 | - |
| Bronchitis, Emphysema, and Asthma | 18 | 17.7 | 1.02 (0.60-1.61) | 9 | 10.0 | 0.90 (0.41-1.71) | 16 | 11.4 | 1.41 (0.81-2.29) |
| Bronchitis | 1 | 1.2 | - | 1 | 0.7 | - | 1 | 0.7 | - |
| Emphysema | 11 | 10.9 | 1.01 (0.50-1.81) | 6 | 6.0 | 0.99 (0.36-2.16) | 14 | 6.8 | 2.07 (1.13-3.48)* |
| Asthma | 6 | 5.5 | 1.08 (0.40-2.36) | 2 | 3.3 | - | 1 | 3.9 | - |
| Pneumoconiosis and Other Respiratory Diseases | 59 | 91.2 | 0.65 (0.49-0.84)** | 31 | 51.0 | 0.61 (0.41-0.86)** | 56 | 56.3 | 1.00 (0.75-1.29) |
| Chronic Obstructive Pulmonary Disease | 40 | 66.8 | 0.60 (0.43-0.82)** | 24 | 37.3 | 0.64 (0.41-0.96)* | 41 | 41.2 | 1.00 (0.71-1.35) |
| Pneumoconiosis/Other Lung Diseases, External Agents | 4 | 6.6 | 0.61 (0.17-1.56) | 3 | 3.5 | - | 6 | 3.8 | 1.60 (0.59-3.48) |
| Asbestosis | 0 | 0 | - | 0 | 0 | - | 0 | 0 | - |
| Silicosis and Anthracosilicosis | 0 | 0 | - | 0 | 0 | - | 0 | 0 | - |
| Digestive Disease | 48 | 66.8 | 0.72 (0.53-0.95)* | 30 | 39.4 | 0.76 (0.51-1.09) | 39 | 44.6 | 0.87 (0.62-1.20) |
| Ulcer of Stomach and Duodenum | 1 | 2.8 | - | 2 | 1.5 | - | 1 | 1.7 | - |
| Cirrhosis of Liver | 21 | 25.3 | 0.83 (0.51-1.27) | 14 | 15.8 | 0.88 (0.48-1.48) | 15 | 18.3 | 0.82 (0.46-1.35) |
| Genitourinary Disease | 26 | 33.9 | 0.77 (0.50-1.12) | 12 | 18.7 | 0.64 (0.33-1.12) | 22 | 20.7 | 1.06 (0.66-1.61) |
| Nephritis and Nephrosis | 19 | 23.9 | 0.80 (0.48-1.24) | 7 | 13.3 | 0.52 (0.21-1.08) | 17 | 14.9 | 1.14 (0.66-1.82) |
| Skin/Subcutaneous Tissue Disease | 2 | 2.6 | - | 1 | 1.5 | - | 2 | 1.7 | - |
| Musculoskeletal Disease & Connective Tissue | 9 | 13.3 | 0.68 (0.31-1.28) | 5 | 7.7 | 0.65 (0.21-1.52) | 3 | 8.8 | 0.34 (0.07-1.00)* |
| All External Causes of Death | 98 | 104.5 | 0.94 (0.76-1.14) | 53 | 65.0 | 0.82 (0.61-1.07) | 80 | 76.8 | 1.04 (0.83-1.30) |
| Accidents | 54 | 67.3 | 0.80 (0.60-1.05) | 34 | 41.3 | 0.82 (0.57-1.15) | 47 | 48.6 | 0.97 (0.71-1.29) |
| Transportation Accidents | 30 | 31.2 | 0.96 (0.65-1.37) | 11 | 19.6 | 0.56 (0.28-1.01) | 25 | 23.3 | 1.07 (0.69-1.58) |
| Motor Vehicle Accidents (MVA) | 23 | 25.4 | 0.90 (0.57-1.36) | 9 | 15.9 | 0.57 (0.26-1.07) | 21 | 18.8 | 1.12 (0.69-1.70) |
| All Other Accidents besides MVA | 31 | 41.3 | 0.75 (0.51-1.06) | 25 | 25.1 | 1.00 (0.64-1.47) | 26 | 29.4 | 0.88 (0.58-1.30) |
| Suicides | 26 | 19.5 | 1.33 (0.87-1.95) | 13 | 12.9 | 1.01 (0.54-1.72) | 16 | 15.2 | 1.05 (0.60-1.71) |
| Homicides and Legal Intervention | 13 | 12.3 | 1.05 (0.56-1.80) | 4 | 7.4 | 0.54 (0.15-1.38) | 16 | 9.0 | 1.78 (1.02-2.90)* |
| Congenital Anomalies | 3 | 4.4 | - | 4 | 2.7 | - | 0 | 3.2 | - |

SMR (95% CI), standardized mortality ratio (95% confidence interval).

▪Expected deaths based on U.S. general population mortality rates.

*Statistically significant at *P* <0.05.

**Statistically significant at *P* <0.01.

MDS, Myelodysplastic Syndrome
