# Supplementary material for: Mortality of 196,826 Men and Women Working in U.S.-Based Petrochemical and Refinery Operations: Update 1979 to 2010
Source: J Occup Environ Med. 2021 Oct 20;64(3):250–62. doi: 10.1097/JOM.0000000000002416 (PMC8887844; doi:10.1097/JOM.0000000000002416)
Supplement: Supplemental Digital Content [file joem-64-0250-s013.docx]

Supplemental Digital Content 9, Table Listing Mortality Results of U.S.-based Petroleum Cohort by Manufacturing Job Titles (1979-2010) – WOMEN

| **Cause of Death** | **SKILLED CRAFTSMEN** | | | **OPERATORS** | | | **LABORERS** | | |
| --- | --- | --- | --- | --- | --- | --- | --- | --- | --- |
|  | **Observed** | **Expected*** | **SMR (95% CI)** | **Observed** | **Expected*** | **SMR (95% CI)** | **Observed** | **Expected*** | **SMR (95% CI)** |
| All Causes | 86 | 79.7 | 1.08 (0.86-1.33) | 269 | 242.4 | 1.11 (0.98-1.25) | 410 | 335.7 | 1.22 (1.11-1.34)** |
| Infectious and Parasitic Diseases | 2 | 2.7 | - | 6 | 8.8 | 0.68 (0.25-1.48) | 11 | 11.8 | 0.93 (0.46-1.66) |
| Tuberculosis | 0 | 0 | - | 0 | 0.1 | - | 0 | 0.2 | - |
| Human Immunodeficiency Virus (HIV) Disease (incl. AIDS) | 0 | 0.8 | - | 1 | 3.0 | - | 4 | 4.1 | - |
| Malignant Neoplasms (MNs) | 27 | 27.3 | 0.99 (0.65-1.44) | 87 | 83.3 | 1.04 (0.84-1.29) | 115 | 108.1 | 1.06 (0.88-1.28) |
| MN of Buccal Cavity and Pharynx | 0 | 0.3 | - | 2 | 0.8 | - | 1 | 1.1 | - |
| MN of Pharynx | 0 | 0.1 | - | 2 | 0.4 | - | 0 | 0.5 | - |
| MN of Digestive Organs and Peritoneum | 6 | 5.1 | 1.18 (0.43-2.57) | 16 | 15.3 | 1.05 (0.60-1.70) | 18 | 20.7 | 0.87 (0.52-1.38) |
| MN of Esophagus | 0 | 0.3 | - | 0 | 0.8 | - | 3 | 1.1 | - |
| MN of Stomach | 0 | 0.5 | - | 0 | 1.4 | - | 0 | 1.9 | - |
| MN of Large Intestine (Colon) | 3 | 1.9 | - | 5 | 5.6 | 0.90 (0.29-2.09) | 8 | 7.7 | 1.04 (0.45-2.05) |
| MN of Rectum | 0 | 0.3 | - | 2 | 1.0 | - | 0 | 1.3 | - |
| MN of Biliary Passages (including Gallbladder)/Liver | 0 | 0.7 | - | 1 | 2.0 | - | 3 | 2.6 | - |
| MN of Liver (Specified Primary or Unspecified) | 0 | 0.3 | - | 1 | 1.0 | - | 2 | 1.3 | - |
| MN of Pancreas | 2 | 1.3 | - | 7 | 3.9 | 1.81 (0.73-3.73) | 3 | 5.2 | 0.57 (0.12-1.68) |
| MN of Respiratory System | 4 | 6.7 | 0.60 (0.16-1.53) | 30 | 20.2 | 1.48 (1.00-2.12)* | 29 | 26.7 | 1.09 (0.73-1.56) |
| MN of Nasal Cavity/Mid Ear/Accessory Sinuses | 0 | 0 | 0.61 (0.17-1.56) | 0 | 0.1 | - | 0 | 0.1 | - |
| MN of Larynx | 0 | 0.1 |  | 0 | 0.3 | - | 0 | 0.4 | - |
| MN of Bronchus, Trachea, Lung | 4 | 6.6 |  | 30 | 19.8 | 1.52 (1.02-2.16)* | 29 | 26.1 | 1.11 (0.74-1.60) |
| MN of Bone | 0 | 0.1 | - | 0 | 0.2 | - | 0 | 0.2 | - |
| MN of Connective Tissue | 1 | 0.2 | - | 0 | 0.8 | - | 2 | 1.0 | - |
| MN of Skin | 1 | 0.4 | - | 0 | 1.4 | - | 1 | 1.7 | - |
| Malignant Melanoma | 1 | 0.4 | - | 0 | 1.2 | - | 1 | 1.5 | - |
| Malignant Mesothelioma | 0 | 0 | - | 1 | 0.2 | - | 0 | 0.2 | - |
| MN of Breast | 4 | 5.8 | 0.69 (0.19-1.77) | 20 | 18.1 | 1.11 (0.68-1.71) | 24 | 22.2 | 1.08 (0.69-1.61) |
| MN of Cervix Uteri | 1 | 0.8 | - | 1 | 2.6 | - | 4 | 3.2 | - |
| MN of Body of Uterus (including Corpus Uteri) | 0 | 0.3 | - | 1 | 0.9 | - | 0 | 1.3 | - |
| MN of Ovary | 2 | 1.6 | - | 5 | 5.0 | 1.01 (0.33-2.36) | 6 | 6.3 | 0.96 (0.35-2.09) |
| MN of Prostate | 0 | 0 | - | 0 | 0 | - | 0 | 0 | - |
| MN of Testicular | 0 | 0 | - | 0 | 0 | - | 0 | 0 | - |
| MN of Bladder and Other Urinary | 0 | 0.2 | - | 2 | 0.7 | - | 0 | 1.0 | - |
| MN of Bladder (Monson) | 0 | 0.2 | - | 2 | 0.7 | - | 0 | 1.0 | - |
| MN of Kidney | 1 | 0.4 | - | 0 | 1.3 | - | 3 | 1.7 | - |
| MN of Central Nervous System (CNS) including Brain | 0 | 0.8 | - | 0 | 2.4 | - | 3 | 2.9 | - |
| MN of Brain | 0 | 0.7 | - | 0 | 2.3 | - | 2 | 2.9 | - |
| MN of Other/Ill-Defined Sites/Secondary Neoplasms | 3 | 1.7 | - | 4 | 5.1 | 0.78 (0.21-2.00) | 11 | 6.8 | 1.61 (0.80-2.88) |
| MN of Lymphatic and Hematopoietic Tissue | 2 | 2.2 | - | 5 | 6.5 | 0.77 (0.25-1.79) | 13 | 8.9 | 1.47 (0.78-2.51) |
| Hodgkin Lymphoma | 0 | 0.1 | - | 1 | 0.3 | - | 0 | 0.4 | - |
| Non-Hodgkin Lymphoma | 1 | 0.8 | - | 3 | 2.4 | - | 5 | 3.3 | 1.53 (0.50-3.57) |
| Nodular/Follicular Lymphoma | 0 | 0 | - | 0 | 0 | - | 0 | 0 | - |
| Reticulosarcoma | 0 | 0 | - | 1 | 0.1 | - | 0 | 0.2 | - |
| T-Cell Lymphoid Variety | 0 | 0 | - | 0 | 0 | - | 0 | 0 | - |
| Lymphosarcoma | 0 | 0 | - | 0 | 0 | - | 0 | 0.1 | - |
| Other Lymphomas | 1 | 0.6 | - | 2 | 2.0 | - | 4 | 2.7 | - |
| Multiple Myeloma | 1 | 0.4 | - | 0 | 1.2 | - | 2 | 1.7 | - |
| Leukemia & Aleukemia | 0 | 0.8 | - | 1 | 2.5 | - | 6 | 3.3 | 1.80 (0.66-3.91) |
| Acute Lymphocytic Leukemia (ALL) | 0 | 0.1 | - | 0 | 0.2 | - | 2 | 0.3 | - |
| Chronic Lymphocytic Leukemia (CLL) | 0 | 0.1 | - | 0 | 0.2 | - | 1 | 0.4 | - |
| Hairy Cell Leukemia | 0 | 0 | - | 0 | 0 | - | 0 | 0 | - |
| Acute Myelocytic Leukemia (AML) | 0 | 0.3 | - | 0 | 1.1 | - | 2 | 1.4 | - |
| Chronic Myelocytic Leukemia (CML) | 0 | 0.1 | - | 0 | 0.3 | - | 1 | 0.4 | - |
| Acute Monocytic Leukemia | 0 | 0 | - | 0 | 0 | - | 0 | 0 | - |
| Chronic Monocytic Leukemia | 0 | 0 | - | 0 | 0 | - | 0 | 0 | - |
| Acute Erythremia and Erythroleukemia | 0 | 0 | - | 0 | 0 | - | 0 | 0 | - |
| Megakaryocytic Leukemia | 0 | 0 | - | 0 | 0 | - | 0 | 0 | - |
| Acute Non-Lymphocytic Leukemia (ANLL) | 0 | 0.4 | - | 0 | 1.1 | - | 2 | 1.4 | - |
| Other/Unspecified Leukemia (besides ANLL, CML, ALL, CLL) | 0 | 0.2 | - | 1 | 0.6 | - | 0 | 0.9 | - |
| Benign/In situ/Uncertain Behavior/Unspecified Neoplasms | 1 | 0.4 | - | 1 | 1.2 | - | 1 | 1.7 | - |
| Benign CNS (including Brain) | 1 | 0 | - | 0 | 0.1 | - | 0 | 0.1 | - |
| Benign Brain | 0 | 0 | - | 0 | 0 | - | 0 | 0 | - |
| Uncertain Behavior/Unspecified - Brain/Spinal Cord | 0 | 0.1 | - | 0 | 0.4 | - | 1 | 0.5 | - |
| All Diseases of Blood and Blood-Forming Organs | 1 | 0.4 | - | 2 | 1.3 | - | 0 | 1.8 | - |
| Aplastic Anemia | 0 | 0 | - | 1 | 0.1 | - | 0 | 0.2 | - |
| All Other Anemias | 1 | 0.1 | - | 1 | 0.4 | - | 0 | 0.6 | - |
| All Other Diseases of Blood-Forming Organs | 0 | 0.1 | - | 0 | 0.4 | - | 0 | 0.6 | - |
| Other Specified Diseases of Blood/Blood-Form Org (including MDS) | 0 | 0.1 | - | 0 | 0.3 | - | 0 | 0.5 | - |
| Endocrine/Nutritional/Metabolic Diseases | 3 | 3.7 | - | 13 | 11.0 | 1.19 (0.63-2.03) | 12 | 14.9 | 0.80 (0.42-1.40) |
| Diabetes Mellitus | 3 | 2.7 | - | 12 | 8.1 | 1.48 (0.76-2.58) | 9 | 11.1 | 0.81 (0.37-1.54) |
| Mental Disorders | 0 | 1.2 | - | 4 | 3.7 | - | 4 | 6.0 | 0.67 (0.18-1.70) |
| Alcoholism | 0 | 0.3 | - | 3 | 1.0 | - | 0 | 1.1 | - |
| Drug Psychosis, Dependence, Poisoning | 1 | 1.1 | - | 3 | 3.8 | - | 8 | 4.7 | 1.71 (0.74-3.36) |
| Nervous System/Sense Organ Disease | 4 | 2.4 | - | 4 | 7.2 | 0.56 (0.15-1.42) | 16 | 10.9 | 1.47 (0.84-2.39) |
| Parkinson's Disease | 0 | 0.2 | - | 1 | 0.5 | - | 2 | 1.0 | - |
| Motor Neuron Disease including Amyotrophic Lateral Sclerosis | 1 | 0.3 | - | 0 | 0.8 | - | 5 | 1.0 | 4.97 (1.61-11.59)** |
| Multiple Sclerosis | 0 | 0.3 | - | 1 | 1.1 | - | 1 | 1.2 | - |
| Circulatory Disease | 21 | 21.4 | 0.98 (0.61-1.50) | 68 | 62.8 | 1.08 (0.84-1.37) | 117 | 95.0 | 1.23 (1.02-1.48)* |
| All Heart Disease | 17 | 15.8 | 1.08 (0.63-1.73) | 55 | 46.4 | 1.19 (0.89-1.54) | 80 | 70.3 | 1.14 (0.90-1.42) |
| Hypertension with Heart Disease | 1 | 1 | - | 2 | 3.1 | - | 9 | 4.4 | 2.05 (0.94-3.90) |
| Ischemic Heart Disease | 13 | 9.6 | 1.35 (0.72-2.31) | 34 | 28.0 | 1.21 (0.84-1.70) | 46 | 43.2 | 1.06 (0.78-1.42) |
| Acute Myocardial Infarction | 1 | 4.3 | - | 10 | 12.6 | 0.79 (0.38-1.46) | 20 | 19.2 | 1.04 (0.64-1.61) |
| Hypertension without Heart Disease | 0 | 0.5 | - | 1 | 1.6 | - | 4 | 2.4 | - |
| Cerebrovascular Disease | 2 | 4.1 | - | 10 | 12.0 | 0.84 (0.40-1.54) | 24 | 18.0 | 1.33 (0.85-1.98) |
| Diseases of Arteries/Veins/Other Circulatory | 2 | 1.0 | - | 2 | 2.9 | - | 9 | 4.3 | 2.07 (0.95-3.94) |
| Aortic Aneurysm | 1 | 0.3 | - | 0 | 0.9 | - | 3 | 1.4 | - |
| Non-Malignant Respiratory Disease | 5 | 6.1 | 0.83 (0.27-1.93) | 22 | 17.9 | 1.23 (0.77-1.86) | 41 | 26.5 | 1.55 (1.11-2.10)* |
| Acute Respiratory Infections except Influenza/Pneumonia | 0 | 0 | - | 0 | 0.1 | - | 0 | 0.1 | - |
| Pneumonia | 1 | 1.3 | - | 2 | 3.8 | - | 4 | 5.8 | 0.68 (0.19-1.75) |
| Influenza | 0 | 0 | - | 0 | 0.1 | - | 0 | 0.1 | - |
| Bronchitis, Emphysema, and Asthma | 0 | 0.8 | - | 5 | 2.5 | 1.97 (0.64-4.60) | 7 | 3.6 | 1.93 (0.78-3.97) |
| Bronchitis | 0 | 0 | - | 0 | 0.1 | - | 1 | 0.2 | - |
| Emphysema | 0 | 0.4 | - | 5 | 1.3 | 3.81 (1.24-8.89)* | 5 | 2.0 | 2.50 (0.81-5.84) |
| Asthma | 0 | 0.3 | - | 0 | 1.1 | - | 1 | 1.4 | - |
| Pneumoconiosis and Other Respiratory Diseases | 4 | 3.9 | - | 15 | 11.4 | 1.32 (0.74-2.18) | 30 | 16.8 | 1.79 (1.21-2.55)** |
| Chronic Obstructive Pulmonary Disease | 4 | 2.8 | - | 11 | 8.1 | 1.36 (0.68-2.43) | 23 | 12.1 | 1.91 (1.21-2.86)** |
| Pneumoconiosis/Other Lung Diseases, External Agents | 0 | 0.2 | - | 1 | 0.7 | - | 3 | 1.1 | - |
| Asbestosis | 0 | 0 | - | 0 | 0 | - | 0 | 0 | - |
| Silicosis and Anthracosilicosis | 0 | 0 | - | 0 | 0 | - | 0 | 0 | - |
| Digestive Disease | 4 | 3.6 | - | 15 | 11.2 | 1.34 (0.75-2.20) | 16 | 14.7 | 1.09 (0.62-1.77) |
| Ulcer of Stomach and Duodenum | 0 | 0.1 | - | 1 | 0.4 | - | 0 | 0.5 | - |
| Cirrhosis of Liver | 2 | 1.6 | - | 9 | 5.2 | 1.74 (0.79-3.30) | 6 | 6.3 | 0.96 (0.35-2.09) |
| Genitourinary Disease | 2 | 1.6 | - | 5 | 4.6 | 1.09 (0.35-2.54) | 12 | 6.7 | 1.79 (0.92-3.12) |
| Nephritis and Nephrosis | 2 | 1.2 | - | 5 | 3.4 | 1.46 (0.48-3.42) | 11 | 4.9 | 2.25 (1.12-4.02)* |
| Skin/Subcutaneous Tissue Disease | 0 | 0.1 | - | 0 | 0.4 | - | 0 | 0.6 | - |
| Musculoskeletal Disease & Connective Tissue | 0 | 0.7 | - | 1 | 2.2 | - | 3 | 3.0 | - |
| All External Causes of Death | 14 | 6.8 | 2.06 (1.13-3.46)* | 34 | 22.4 | 1.52 (1.05-2.12)* | 53 | 28.3 | 1.87 (1.40-2.45)** |
| Accidents | 8 | 4.2 | 1.91 (0.83-3.77) | 20 | 13.7 | 1.46 (0.89-2.25) | 33 | 17.7 | 1.87 (1.29-2.62)** |
| Transportation Accidents | 6 | 2.1 | 2.90 (1.06-6.31)* | 12 | 6.9 | 1.74 (0.90-3.03) | 17 | 8.7 | 1.94 (1.13-3.11)* |
| Motor Vehicle Accidents (MVA) | 5 | 1.7 | 3.02 (0.98-7.04) | 12 | 5.6 | 2.13 (1.10-3.73)* | 17 | 7.1 | 2.39 (1.39-3.83)** |
| All Other Accidents besides MVA | 3 | 2.5 | - | 8 | 8.0 | 1.00 (0.43-1.97) | 16 | 10.4 | 1.54 (0.88-2.50) |
| Suicides | 3 | 1.4 | - | 6 | 4.6 | 1.29 (0.47-2.81) | 11 | 5.4 | 2.02 (1.01-3.62)* |
| Homicides and Legal Intervention | 3 | 0.8 | - | 7 | 2.9 | 2.44 (0.98-5.04) | 9 | 3.7 | 2.42 (1.11-4.60)* |
| Congenital Anomalies | 0 | 0.3 | - | 0 | 0.9 | - | 0 | 1.2 | - |

SMR (95% CI), standardized mortality ratio (95% confidence interval).

▪Expected deaths based on U.S. general population mortality rates.

*Statistically significant at *P* <0.05.

**Statistically significant at *P* <0.01.

MDS, Myelodysplastic Syndrome
